# Exploring the feasibility of using the ICER Evidence Rating Matrix for Comparative Clinical Effectiveness in assessing treatment benefit and certainty in the clinical evidence on orphan therapies for paediatric indications

**DOI:** 10.1186/s13023-023-02701-w

**Published:** 2023-07-20

**Authors:** Jaro Wex, Monika Szkultecka-Debek, Mariola Drozd, Sarah King, Natasa Zibelnik

**Affiliations:** 1 Global Market Access & HEOR, EUSA Pharma Ltd, Third Floor, Breakspear Park, Breakspear Way, Hemel Hempstead, HP2 4TZ UK; 2 Qualitas Vitae Institute Foundation, Warsaw, Poland; 3 Medical University of Lublin, Lublin, Poland; 4 Brighton, UK; 5 Manchester, UK

**Keywords:** Orphan drugs, Rare diseases, Quality of evidence, Paediatric population, Health benefit, Effect size, Uncertainty of estimate, Assessment framework

## Abstract

**Background:**

The evaluation of clinical evidence takes account of health benefit (efficacy and safety) and the degree of certainty in the estimate of benefit. In orphan indications practical and ethical challenges in conducting clinical trials, particularly in paediatric patients, often limit the available evidence, rendering structured evaluation challenging. While acknowledging the paucity of evidence, regulators and reimbursement authorities compare the efficacy and safety of alternative treatments for a given indication, often in the context of the benefits of other treatments for similar or different conditions. This study explores the feasibility of using the Institute for Clinical and Economic Review (ICER) Evidence Rating Matrix for Comparative Clinical Effectiveness in structured assessment of both the magnitude of clinical benefit (net health benefit, NHB) and the certainty of the effect estimate in a sample of orphan therapies for paediatric indications.

**Results:**

Eleven systemic therapies with European Medicines Agency (EMA) orphan medicinal product designation, licensed for 16 paediatric indications between January 2017 and March 2020 were identified using OrphaNet and EMA databases and were selected for evaluation with the ICER Evidence Rating Matrix: burosumab; cannabidiol; cerliponase alfa; chenodeoxycholic acid (CDCA); dinutuximab beta; glibenclamide; metreleptin; nusinersen; tisagenlecleucel; velmanase alfa; and vestronidase alfa. EMA European Public Assessment Reports, PubMed, EMBASE, the Cochrane Library, Clinical Key, and conference presentations from January 2016 to April 2021 were searched for evidence on efficacy and safety. Two of the identified therapies were graded as “substantial” NHB: dinutuximab beta (neuroblastoma maintenance) and nusinersen (Type I SMA), and one as “comparable” NHB (CDCA). The NHB grade of the remaining therapies fell between “comparable” and “substantial”. No therapies were graded as having negative NHB. The certainty of the estimate ranged from “high” (dinutuximab beta in neuroblastoma maintenance) to “low” (CDCA, metreleptin and vestronidase alfa). The certainty of the other therapies was graded between “low” and “high”. The ICER Evidence Rating Matrix overall rating “A” (the highest) was given to two therapies, “B+” to 6 therapies, “C+” to five therapies, and “I” (the lowest) to three therapies. The scores varied between rating authors with mean agreement over all indications of 71.9% for NHB, 56.3% for certainty and 68.8% for the overall rating.

**Conclusions:**

Using the ICER Matrix to grade orphan therapies according to their treatment benefit and certainty is feasible. However, the assessment involves subjective judgements based on heterogenous evidence. Tools such as the ICER Matrix might aid decision makers to evaluate treatment benefit and its certainty when comparing therapies across indications.

**Supplementary Information:**

The online version contains supplementary material available at 10.1186/s13023-023-02701-w.

## Background

Clinical evidence established in clinical trials and real-world studies is essential for the marketing authorisation, reimbursement, and ultimately, patient access to new medical technologies [1]. Several evidence grading tools have been developed to facilitate the assessment of such evidence and inform decision making. These include Joanna Briggs Institute tools, GRADE, the ICER Evidence Rating Matrix for Comparative Clinical Effectiveness and the ESMO MCBS [2–7]. Evidence can be viewed along two dimensions: (1) the estimate of treatment (health) benefit considering efficacy and safety, and (2) confidence in the estimate, i.e., the certainty or the likelihood of the estimate of the treatment effect being *true* [8]*.* Evaluation of health benefit involves assessing the clinical relevance of reported outcome measures in the context of a specific disease, as well as estimating the magnitude of the effect versus comparator treatments. The relevance of outcome measures, for example surrogate endpoints, depends on their mechanism of action and link to other endpoints, such as symptom scores, quality of life or survival. The clinical relevance of the treatment effect size (magnitude) also needs to be considered and can be judged, for example, against the minimal clinically important difference (MCID) [9]. MCID is the smallest difference in score in the domain of interest that patients or clinicians perceive as beneficial and which would support, in the absence of troublesome side effects and excessive cost, a change in the patient’s management. The level of certainty in the estimate of the health benefit is assessed by considering the quality of the body of evidence supporting the technology.

Achieving high certainty that a technology is effective and safe may be particularly difficult in the case of orphan therapies for rare diseases, due to challenges associated with the populations being studied and the study designs that may need to be adopted [10–16].

The lack of validated instruments to assess efficacy and certainty in the context of rare diseases compounds the challenges involved in evaluating each individual treatment and is even more of an issue when comparing the value of treatments for different diseases. Regulatory and reimbursement authorities conducting health technology assessments (HTA) have considered the barriers to evidence generation and high unmet medical need of patients affected by rare diseases, allowing for more flexibility in the assessment of orphan therapies [15, 17]. Agencies often consider the size of the incident or prevalent population and adapt their requirements accordingly. Adaptation may involve accepting lower levels of evidence and validated surrogate endpoints and using registry data or historical controls to establish long-term safety and efficacy evidence or to obtain information on the duration of treatment [15, 18, 19]. Authorities may also offer conditional approval or reimbursement of orphan drugs if the manufacturer commits to providing further evidence in the form of post-marketing research or generation of additional data. Such commitments are often necessary to ensure that funding is available for treatments with a likelihood of benefit to address the high unmet need in patients with rare diseases. Crucially, the treatment of rare diseases is typically associated with a high cost per patient. Despite small eligible populations and limited budget impact, an increasing number of new high-cost technologies might prompt explicit or implicit comparisons across indications to inform resource allocation [20, 21]. In this study, we aimed to explore the feasibility of using the Institute for Clinical and Economic Review Evidence Rating Matrix for Comparative Clinical Effectiveness (ICER Evidence Rating Matrix) as an evaluation framework to compare across indications, considering the two dimensions of health benefit and certainty in the treatment of paediatric rare diseases.

## Methods

### Aims

The aim of the study was to test the feasibility of using the ICER Evidence Rating Matrix, (referred to below as the ICER Matrix) to guide the comparative grading of the benefit-risk and degree of uncertainty associated with each therapy [4].

### Features of the ICER matrix

**Fig. 1:**
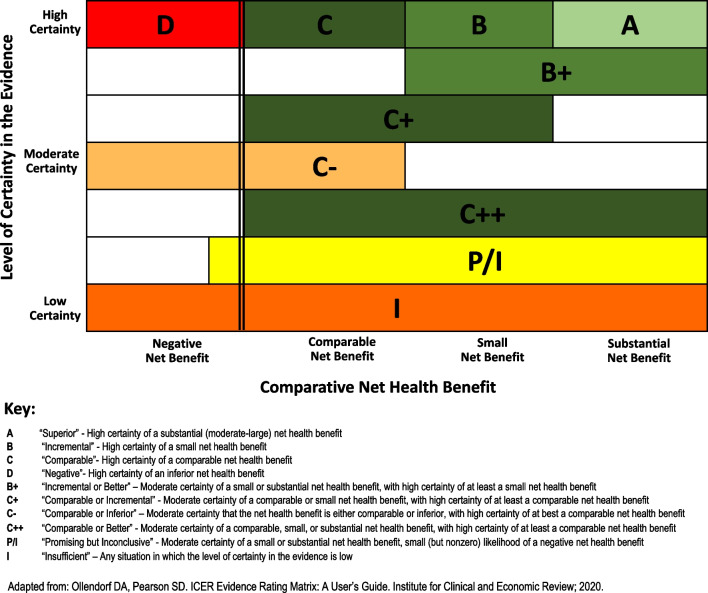
ICER Evidence Rating Matrix (Adapted from Ollendorf and Pearson, 2020)

We selected the ICER Matrix, based on an evaluation of a range of tools, as in our opinion it is the only tool explicitly intended for assessing certainty alongside establishing the magnitude of added benefit [22]. The ICER Matrix captures the magnitude of the difference between a therapy and its comparator in terms of comparative net health benefit (NHB). NHB is the balance between clinical benefits and risks or adverse effects. The ICER Matrix expresses magnitude as “negative”, “comparable”, “small”, or “substantial”. The level of certainty in the estimate of the comparative NHB is defined in the ICER Matrix as “low”, “moderate”, or “high” (Fig. 1) [4].

### Identification and selection of therapies

Given the complexity of issues surrounding the assessment of evidence for orphan therapies, the aim of this study was to test the feasibility of using the ICER Matrix to compare therapies for different rare diseases in a sample of paediatric indications sufficient to explore the factors contributing to the assessment.

All non-topical systemic therapies with European Medicines Agency (EMA) orphan medicinal product designation licensed for paediatric (< 18 years of age) indications between January 2017 and March 2020 were identified using the OrphaNet Report and the EMA databases [23, 24]. The OrphaNet Report and the EMA databases were accessed in April 2021.

### Identification of evidence on the efficacy and safety of the therapies

Evidence on the efficacy and safety of each designated orphan therapy was identified from the EMA European Public Assessment Reports (EPARs), published literature and conference presentations in searches undertaken in May 2021. We searched PubMed, Embase, the Cochrane Library and Clinical Key databases systematically using search strings to capture two concepts: Therapy AND Indication (see Additional file 1: Appendix 1 for the detailed strategies). Leading international, European, and American congresses on rare diseases were identified and searched manually for conference presentations in the specific therapy areas under consideration from 2015 onwards (see Additional file 1: Appendix 1).

Studies reporting evidence on the efficacy and safety of each designated orphan therapy had to meet the following criteria to be eligible for inclusion in this study:

- Population: Participants aged under 18 years (where the approved indication was not restricted to children, clinical trials including participants aged under 18 years were considered eligible).
- Therapies: Systemic therapies approved for paediatric indications by the EMA between January 2017 and March 2020 identified from searches of OrphaNet Report and the EMA databases. Therapies of any duration and at any dosing frequency were eligible.
- Outcomes:
  - Efficacy and safety.
  - Quality of life.
- Study designs: Phase II, III or IV clinical trials (including subgroup analyses specific to the indications), retrospective analyses, real-world studies, or meta-analyses were eligible. Studies reporting any length of follow-up were eligible. Phase I data, preclinical research, and case reports were not eligible.
- Publication dates: Studies published between January 2015 and April 2021 were eligible. Evidence published prior to 2015 was not searched, as it was assumed that the EMA would have included relevant evidence published two or more years prior to its decision in the EPAR. The search, however, did include evidence, such as longer-term follow-up results, published after EMA decisions.

One reviewer screened the search results against the inclusion and exclusion criteria to identify eligible studies, and a second reviewer checked all the decisions. The reviewers discussed any discrepancies, to reach consensus.

### Data extraction

The following data were extracted for each therapy:

- Population age.
- Intervention details.
- Comparator details, where available.
- Outcomes: measures and results for all outcomes as reported in the trials (primary efficacy outcomes, clinically relevant secondary efficacy outcomes, and safety). Survival and quality of life outcomes were also extracted where reported.
- Study design details for phase II, III or IV clinical trials (including subgroup analyses specific to indications), retrospective analyses, real-world studies, or meta-analyses.

### Evidence assessment

The assessment of the efficacy benefit of each of the therapies was based on comparison of reported effect size to clinically meaningful/relevant change, i.e., to the minimal clinically important difference (MCID) [9] that had been established for each outcome, with reference to the specific or similar condition where possible. MCID may help to determine whether a therapy is likely to provide worthwhile changes in a patient’s health status and researchers use it to judge the effectiveness of therapies from the patient’s point of view. The magnitude of the efficacy benefit was considered along with safety: a 30% cut-off frequency of Grade Three and Four adverse events was used for all therapies, following the ESMO Magnitude of Clinical Benefit Scale (MCBS) adjustment rule for orphan diseases (Form 3) [5, 25]. Even though, in principle, ESMO MCBS Form 3 could be suitable for the assessment of health benefit of all orphan therapies with single-arm evidence, we only used this tool to assess the magnitude of NHB of anti-cancer therapies, separately for likely curative and likely non-curative therapies [5].

Grading of certainty was based on the strength of the evidence, taking account of risk of bias, the generalizability of the trial population to the population within the licensed indication, the precision of the estimates of outcomes, consistency between studies, the directness of the comparison and the type of efficacy outcomes (hard, e.g. survival or surrogate, e.g. biomarker). To further explore the certainty associated with each therapy, the duration of each therapy was estimated based on the product’s EMA Summary of Product Characteristics and dosing reported in clinical trials. Therapies were categorised as having either a defined or an undefined duration.

Four authors individually rated the evidence by assigning grades to the dimensions of comparative NHB and level of certainty in the evidence in the ICER Evidence Rating Matrix (Fig. 1). Four grades of NHB were used: “negative”, “comparable”, “small” and “substantial”. For certainty, the ICER Matrix allows five grades, to offer intermediate scores of “low-to-moderate” and “high-to-moderate”. In cases where scores were not unanimous, the rating was reported accordingly, e.g. small/substantial for NHB or high-to-moderate/high for certainty. The two dimensions were then combined and the ICER Matrix methodology was used to assign the overall rating. For example, an “A” rating indicates “high certainty of a substantial (moderate-large) NHB” and a “C” rating indicates “high certainty of a comparable NHB” [4]. An “I” rating indicates “insufficient” certainty when the overall level of certainty in the evidence is low. Where authors disagreed on the overall rating, they discussed the discrepancies and came to a consensus agreement on a summary rating.

Quantitative evidence syntheses or statistical analyses were not conducted, however the individual evaluations took account of the statistical significance reported in the studies.

The authors considered five aspects of evidence (factors) that could contribute to the assessment of the magnitude of a therapy’s NHB:

- Clinical relevance of the effect size.
- Clinical relevance of efficacy endpoints.
- Impact on health-related quality of life.
- Clinical relevance of adverse events.
- Potential curative effect.

The authors considered five aspects of evidence, reflecting the eligibility criteria, when considering certainty:

- Population (number of patients studied, eligibility criteria).
- Intervention (concomitant treatments, duration of treatment).
- Comparator (comparator consistency, comparator relevance).
- Outcomes (variation of outcomes between studies, statistical significance of outcomes).
- Study design (types of clinical studies, duration of studies).

Each aspect received a weight from one to three to reflect its contribution to the overall assessment, specifically its impact on the assessment grade (as opposed to the magnitude or certainty of the actual benefit of the therapy). The weights were converted to percentages so that the results could be compared across therapies. Additional file 2: Appendix 2 shows an example of the rating table used for assessments.

The authors’ degree of agreement was measured to assess the levels of subjectivity in their assessments. If all the authors scored differently, agreement was quantified as zero. If three of the four scores were different, the agreement was quantified as one. If two authors proposed one score and two authors proposed a second score, resulting in two different scores overall, agreement was two. If three authors agreed and there was one dissenter, agreement was three. When all four authors agreed, the agreement was four. This produced a five-point scale from zero (maximum divergence in scoring) to four (full agreement), which was converted to a percentage.

## Results

### Eligible therapies

**Fig. 2:**
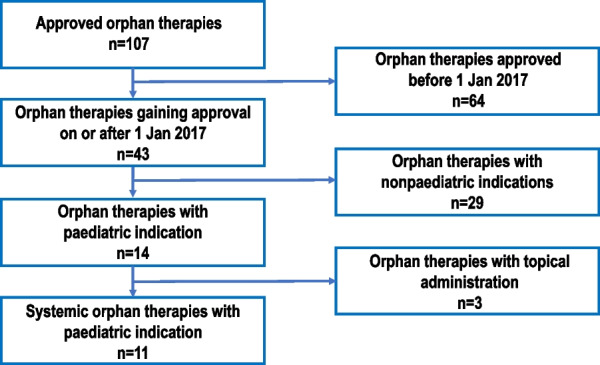
Selection of orphan therapies for paediatric rare diseases approved by the European Medicines Agency between January 2017 and March 2020

Eleven systemic therapies, for 16 indications, approved by the EMA as orphan medicinal products for use in paediatric rare diseases were identified: burosumab (X-linked hypophosphatemia), cannabidiol (Dravet syndrome and Lennox–Gastaut syndrome), cerliponase alfa (neuronal ceroid lipofuscinosis 2 (CLN2) disease), CDCA (inborn errors of primary bile acid synthesis), dinutuximab beta (high-risk maintenance and relapsed/refractory neuroblastoma), glibenclamide (neonatal diabetes mellitus), metreleptin (generalised and partial lipodystrophy), nusinersen (Type I, II/III and presymptomatic spinal muscular atrophy (SMA)), tisagenlecleucel (relapsed/refractory B-cell acute lymphoblastic leukaemia (ALL)), velmanase alfa (mild to moderate alpha-mannosidosis), and vestronidase alfa (mucopolysaccharidosis VII). Figure 2 shows the selection process.

### Evidence identification and extraction

The literature search for evidence on efficacy and safety of the 11 orphan therapies identified a total of 3,132 citations. Following deduplication and removal of irrelevant publications, 1,353 publications and conference presentations were screened for relevance. 115 publications reporting 68 studies (Additional file 3: Appendix 3) were considered eligible and were included in the analysis. The extracted evidence is presented in Additional file 3: Appendix 3. Based on the extracted evidence, narrative summaries for all therapies were compiled to facilitate the assessment process (Additional file 4: Appendix 4).

### Assessment of net health benefit for the therapies

The highest NHB grade (substantial) was given to nusinersen in Type I SMA and dinutuximab beta in a neuroblastoma maintenance population. The NHB of CDCA for inborn errors of primary bile acid synthesis was graded “comparable”. The remaining therapies were deemed to have intermediate NHB between “comparable” and “substantial”, i.e. none of the therapies were graded as having “negative” NHB. Table 1 shows the details of the grading for each therapy and Table 2 lists the factors considered in the assessment along with their contribution to the grade across all therapies. Using ESMO-MCBS [26], the NHB of dinutuximab beta was rated A (substantial, using Form 1) for the treatment of high-risk maintenance neuroblastoma and A/4 (substantial, using two separate forms: 2A and 1) for the treatment of relapsed refractory neuroblastoma, with maintenance treatment considered to be potentially curative. The NHB of tisagenlecleucel for the treatment of ALL was rated three (small/substantial) using ESMO-MCBS Form 3. These NHB ratings were concordant with evaluations based on MCID, except in the case of dinutuximab beta in relapsed/refractory neuroblastoma, which was rated more conservatively as small/substantial using MCID.

**Table 1:** Net health benefit, certainty and ICER Matrix rating for 11 therapies

Treatment	Net health benefit [ESMO-MCBS*]	Potentially curative?	Grade 3–4 adverse effects < 30%	Certainty	ICER matrix rating
Burosumab (X-linked hypophosphatemia)	Comparable/small	−	+	Moderate/high-to-moderate	B+
Cannabidiol (Dravet syndrome)	Small/substantial	−	+	Moderate/high-to-moderate	B+
Cannabidiol (Lennox–Gastaut syndrome)	Small/substantial	−	+	Moderate/high-to-moderate	B+
Cerliponase alfa (neuronal ceroid lipofuscinosis 2 (CLN2) disease)	Comparable/small	−	− (Control NR)	Moderate	C+
Chenodeoxycholic acid (CDCA) (inborn errors of primary bile acid synthesis)	*Comparable*	−	NR	Low/low-to-moderate	*I*
**Dinutuximab beta (high-risk maintenance neuroblastoma)**	**Substantial [ESMO MCBS Form 1: A]**	+	−	**High**	**A**
Dinutuximab beta (relapsed/refractory neuroblastoma)	Small/substantial [ESMO Form 2A: 4; Form 1: A]	+	−	Moderate/high-to-moderate	B+
Glibenclamide (neonatal diabetes mellitus)	Comparable/small	−	+	Moderate/high-to-moderate	C+
Metreleptin (generalised lipodystrophy)	Comparable/small	−	+	Moderate/high-to-moderate	C+
Metreleptin (partial lipodystrophy)	Comparable/small	−	+	Low/low-to-moderate	*I*
**Nusinersen (Type I spinal muscular atrophy (SMA))**	**Substantial**	−	+	High-to-moderate/high	**A**
Nusinersen (Type II/III spinal muscular atrophy (SMA))	Small/substantial	−	+	Moderate/high-to-moderate	B+
Nusinersen (presymptomatic spinal muscular atrophy (SMA))	Comparable/small	−	+	Low-to-moderate/moderate	C+
Tisagenlecleucel (relapsed/refractory B-cell acute lymphoblastic leukaemia (ALL))	Small/Substantial [ESMO MCBS Form 3: 3]	+	−	Moderate/high-to-moderate	B+
Velmanase alfa (mild to moderate alpha-mannosidosis)	Comparable/small	−	+	Low-to-moderate/moderate	C+
Vestronidase alfa (mucopolysaccharidosis VII)	Comparable/small	−	+	*Low*	*I*

ESMO-MCBS, Magnitude of Clinical Benefit Scale; NR, Not reported

ICER Matrix ratings: A (superior), B + (incremental or better), C + (comparable or incremental), I (insufficient)

Treatments with the highest scores are in bold and treatments with the lowest scores are in italics

*ESMO MCBS was only considered for NHB of anti-cancer therapies, but ESMO MCBS Form 3 AE 30% cut-off was used for all therapies

**Table 2:** Factors contributing to the ratings of net health benefit and certainty

Contributing factor	Relative contribution to net health benefit assessment (%)
Clinical relevance of effect size	23.8*
Clinical relevance of efficacy endpoints	23.2
Impact on quality of life	16.7
Clinical relevance of adverse events	17.6
Potential curative effect	18.7

Contributing factor	Relative contribution to certainty assessment (%)
Population	20.7
Intervention	21.0
Comparator	21.0
Outcomes	18.4
Study design	18.9

*The percentage contribution is a mean over 16 indications assessed by four authors

Burosumab, cannabidiol, glibenclamide, metreleptin, nusinersen, velmanase alfa and vestronidase alfa had less than 30% frequency of Grade Three and Four adverse events, which was accounted for in the NHB rating.

### Assessment of certainty of the effect of the therapies

The certainty of the effect estimate was influenced by all aspects of the eligibility criteria (PICOS framework): the treated population, intervention, comparator, outcomes, and study design. Table 1 shows the details of the certainty grading for each therapy and Table 2 shows the contribution of the PICOS factors to the grade. Table 3 lists the key factors identified in the assessment specific to each therapy. Estimates of the benefit of two therapies were assigned high or high-to-moderate certainty (dinutuximab beta in a neuroblastoma maintenance population and nusinersen for Type I SMA), three were assigned low or low-to-moderate certainty (CDCA, metreleptin in partial lipodystrophy and vestronidase alfa) and the remaining six were graded as intermediate between “low” and “high” certainty.

**Table 3:** Key factors considered in the assessment of certainty in relation to 11 therapies

Treatment	Key factors considered in certainty assessment
Burosumab	> 100 paediatric patients studied Multicentre, randomised, open-label studies; multicentre single-arm study Conventional therapy comparator (oral phosphates and active Vitamin D analogues) Limited evidence in adolescents and patients with milder severity Rickets severity, growth, functional ability, pain outcomes Follow up data up to 64 weeks Different dosing across studies, undefined treatment duration, potentially lifelong treatment
Cannabidiol	> 1,000 patients studied, including adults Multicentre, randomised, double-blind, placebo-controlled controlled studies; open label extensions Variation in conventional clinical management Unknown relationship between reduced seizure frequency and overall survival Quality of life outcomes, including patient and carer-reported Follow-up data up to 3 years Dosing based on individual clinical response with undefined treatment duration, potentially lifelong
CDCA (chenodeoxycholic acid)	> 150 patients studied, including adults Multicentre and single centre retrospective studies Comparative data from literature Patient populations with different symptoms/disability, disease duration and treatment duration Metabolic outcomes, clinical symptoms, quality of life, disability scores Median follow-up > 8 years Dosing adjusted individually with undefined treatment duration, potentially lifelong (replacement therapy)
Cerliponase alfa	> 20 patients studied 1 multicentre, single-arm study, natural history historical control study Motor-language score, quality of life outcomes Differences in definitions of symptom severity scores in treated patients and historical controls Follow-up data up to 2 years, 1 year for historical controls Undefined treatment duration, potentially lifelong (replacement therapy)
Dinutuximab beta	> 1000 paediatric patients studied Multicentre, open-label prospective study with historical control from non-concurrent randomisation of the same trial; multicentre single-arm prospective studies with historical controls Conventional therapy comparator (non-immunotherapy) Different populations in maintenance and relapsed/refractory Overall survival, event-free and progression-free survival endpoints Follow-up data up to 7 years Defined dose and treatment duration (limited to 5 cycles); 5-day or 10-day infusion regimens
Glibenclamide	> 150 paediatric patients studied Multicentre single-arm and single-centre single-arm prospective studies Lack of comparative effectiveness data; established evidence base in other types of diabetes (different formulations) Withdrawal of insulin therapy, glycaemic control, neuro-psychomotor outcomes, acceptability of the oral suspension formulation Median follow-up > 10 years Undefined treatment duration, potentially lifelong
Metreleptin	> 200 patients studied, including adults Multicentre, single-arm and single-centre single arm prospective studies; multicentre retrospective study Heterogeneous population comprising various types of lipodystrophy Glycaemic control, metabolic outcomes Follow-up over 14 years Dose adjustment based on response to treatment, undefined treatment duration, potentially lifelong
Nusinersen	> 500 paediatric patients studied Multicentre, randomised, double-blind, sham-controlled studies; multicentre, single-arm open label study Differences in SMA subtype populations Motor function, event-free survival, overall survival outcomes Follow-up over 6 years Undefined treatment duration, potentially lifelong, different dosing across trials
Tisagenlecleucel	> 250 patients studied, including adults Multicentre and single-centre single-arm studies Unadjusted/naïve comparisons to comparator therapies Response rates, event-free survival, overall survival, quality of life outcomes Follow-up over 3 years One-time treatment; different numbers of infusions in trials; lag time to prepare engineered cells potentially affecting eligibility
Velmanase alfa	> 50 patients studied, including adults Multicentre, double-blind, placebo-controlled study; “integrated database” including several small single-arm cohort studies Heterogeneity in severity of the disease of included patients Metabolic, functional and quality of life outcomes Follow-up up to 48 months Undefined treatment duration, potentially lifelong
Vestronidase alfa	> 25 patients studied Multicentre, blind-start, single crossover, placebo-controlled study; multicentre, single-arm study Metabolic, functional and quality of life outcomes Heterogeneity in severity of the disease of included patients Follow-up up to 48 weeks (extension ongoing) Unclear optimal treatment duration

### ICER matrix overall rating

The Matrix rating (combining NHB and certainty) was A (superior) for two therapies, B + for 6 therapies, C + for five therapies, and I (insufficient) for three therapies (see Table 1).

The mean author agreement score across all therapies was 71.9% (range: 50–100%) for NHB, 56.3% (25–100%) for certainty and 68.8% (50–100%) for the overall rating (prior to consensus decision) over the two dimensions. Additional file 4: Appendix 4 provides additional details on the rating of NHB and certainty by the four authors.

## Discussion

This study explored the feasibility of using the ICER Evidence Rating Matrix to compare the magnitude of net health benefit and certainty around the estimate of benefit across a sample of orphan therapies, focusing on paediatric indications where evidence generation is particularly challenging. We found a comparison to be feasible, allowing for therapies to be graded separately in the two dimensions.

The ICER Matrix addresses the level of uncertainty or confidence specifically in the outcomes, even when the orphan indication has imposed limitations on the study design, population eligibility criteria, and comparator choice, each of which can affect an interpretation of the magnitude and certainty of outcomes. The ICER Matrix only combines NHB and certainty, allowing for flexibility and pragmatism when estimating the two dimensions [27].

Using the ICER Matrix facilitated the exploration of aspects of the evidence which contributed to the grading. Although this study has highlighted subjectivity inherent in the method, the level of inter-rater agreement in the rating of NHB (71.9%) can be considered high, particularly given that in all ratings at least two authors proposed the same score and there were no instances of fully divergent scoring. It is noteworthy, that all five factors considered were found to have contributed markedly to the assessment of NHB, with the clinical relevance of the efficacy endpoints and effect size, as well as the potential curative effect of a therapy assigned the greatest weight [11, 12, 28, 29].

Regulatory evaluation considers individual therapies for specific conditions in the context of relevant comparators, however comparisons across therapies for different diseases are likely to influence the evaluation process, explicitly or implicitly, as both the magnitude of the treatment effect and its associated certainty vary widely and provide context and reference points. Such comparisons between therapies are more explicit in health technology assessment, where the added benefit of therapies is typically assessed in the context of clinical practice and is often categorised or quantified using universal metrics, such as Life Years (LYs), Quality-Adjusted Life Years (QALYs) or Quality-adjusted Time Without Symptoms of disease or Toxicity (QTWIST) [30]. The metrics allow for judgements about which therapies offer greater net benefit to patients, with the strength of recommendations reflecting the certainty of the evidence. Although it is feasible to use universal metrics of health benefit to compare therapies across indications, such an approach is not well-aligned with the unique considerations of orphan therapies [31], omitting patient-relevant issues such as the value of hope and real option value where no other therapy is available [32, 33]. Even so, the ICER Matrix combined evidence ratings were found to be correlated with incremental QALYs [34].

Our study explored the various aspects of certainty around the NHB estimates using the eligibility criteria (PICOS) as a framework. Annemans and Makady distinguished four types of uncertainties in the assessment of health technologies in their TRUST4RD (Tool for Reducing Uncertainties in the evidence generation for Specialised Treatments for Rare Diseases) tool [35]:

- Uncertainties related to the size and characteristics of the population.
- Uncertainties related to the natural history of the disease and its current management.
- Uncertainties related to the new treatment.
- Uncertainties related to the health ecosystem.

In addition, the TRUST (TRansparent Uncertainty ASsessmenT) framework [36] for evaluating uncertainty in health technologies includes:

- The context or scope, including the population, intervention, comparator, and outcomes.
- The selection of evidence.
- Relative effectiveness estimates.
- Adverse events.
- Aspects related to health economic evaluation, such as costs, utilities, time horizon and perspective.

The TRUST tool also addresses several aspects of uncertainty including transparency issues, methodological issues, imprecision, indirectness, and unavailability. The TRUST authors abandoned numerical scoring of uncertainties for their impact due to difficulties with the scoring process and substantial inter-rater variability experienced when applying the tool [36]. They argued the latter issues could create a false impression of quantified precision, while the scores only reflected subjective perception. Instead, they considered a simple assessment of impact more appropriate, suggesting assessments of “likely high”, “likely low”, or “likely no impact”. Our assessment of certainty largely overlapped with TRUST and TRUST4RD, however aspects related to disease management, wider health system and health economics, were not considered. We found that all five PICOS criteria contributed to the assessment, with weights of at least 20% assigned to the population, intervention, and comparator. Inter-rater agreement for certainty (56.3%) was lower than that for NHB, which is to be expected given the more subjective nature of this dimension of the ICER Matrix.

Several systems and tools have been developed to assess certainty and risk of bias to grade levels of evidence, but few are specific to rare diseases. Evaluation of the quality or certainty of evidence is subjective, as it reflects our confidence in evidence, which is an inherently subjective concept [37]. An assessment of the strength of evidence, according to the widely used GRADE approach, is based on the design of clinical studies, which is often an issue in the case of orphan therapies [3]. The developers of GRADE recognize that “the assessment of evidence quality is a subjective process, and GRADE should not be seen as obviating the need for or minimizing the importance of judgment or as suggesting that quality can be objectively determined” [38]. Indeed, GRADE’s rules for downgrading and upgrading of rating of evidence are particularly subjective, with arbitrary increments of 1 or 2 applied to reflect changes in certainty [39]. GRADE also does not allow for downgrading RCTs that are unbalanced or poorly designed and penalizes potentially less biased large observational studies, such as those that enrol all patients with a given rare condition. It should be noted that a modified GRADE approach has been developed by the American College of Chest Physicians (ACCP), whereby “exceptionally strong” observational studies may be graded 1A, while evidence from observational studies, case series or RCTs with serious flaws or indirect evidence can be graded 1C, if the benefits clearly outweigh the risk and if the recommendation can apply to most patients in many circumstances [40].

According to GRADE, observational evidence is considered lower quality than RCT evidence, but both RCTs without important limitations and overwhelming evidence from observational studies are ranked as “high quality.” The system then allows upgrading or downgrading based on other factors, such as the size of the effect, consistency, precision, potential confounding, and bias [2, 3]. Crucially, GRADE emphasises the quality of individual studies and does not facilitate rating of a body of evidence comprising multiple study designs, i.e., RCTs and observational studies. Even if individual studies have a low level of certainty, the accumulation of evidence from early phase trials, observational studies and real-world experience can increase confidence in a therapy if they all show consistent findings, while long-term follow-up can confirm the magnitude of effect of therapy. In addition, rationale from basic science and mechanism of action is not considered in GRADE, as it is not deemed to be part of the evidence base. However, in orphan therapies the evidence is often complex, its interpretation relies on the mechanistic plausibility of surrogate outcomes and is often informed by clinical experience from similar conditions. One suggested solution to these challenges is the explicit inclusion of all aspects of evidence in an overall evaluation of certainty within a Bayesian framework using explicit priors and likelihoods for transparency [4, 37].

Small numbers of studies and their limited size is a major factor affecting certainty. Large sample sizes and RCTs can be difficult to achieve in the context of rare diseases, as discussed earlier. If RCTs are not feasible, options include limiting the number of patients randomised to placebo, with implications for the study power and statistical analyses, or using single-active-arm trial designs, supported by within-patient or historical controls. Using ratings based on the ICER Matrix, we found that for health-related net benefit, nusinersen for Type I SMA and dinutuximab beta in maintenance therapy appear to have the highest overall rating (A), followed by burosumab, cannabidiol, dinutuximab beta for RRNB, nusinersen for Type II/III SMA and presymptomatic SMA, and tisagenlecleucel (all graded B+). Most of these therapies have been studied in large numbers of patients (dinutuximab beta has been studied in more than 1000 patients, cannabidiol in more than 700 patients and nusinersen in more than 500) and in RCTs, which showed statistically significant and clinically meaningful changes in endpoints following treatment [41–43]. Importantly, dinutuximab beta, nusinersen and tisagenlecleucel all have results for overall survival [41, 43–45].

We attempted to capture curative intent of treatment in the assessment of NHB. Even though there is lack of consensus on what constitutes a cure, curative treatments are evaluated differently [26] and the regulatory process considers curative intent, even if claim of “cure” is not included in the label. The Institute for Clinical and Economic Review has also proposed a concept of high impact “single and short-term therapies” (SSTs). SSTs are therapies involving a single intervention or a course of treatment of less than one year that are either potential cures that can eradicate a disease or condition or high-impact therapies that can produce sustained major health gains or halt the progression of significant illnesses [46]. Although cure would be the most desirable goal for all therapy areas, many of the currently approved orphan therapies can only provide symptom relief or replace dysfunctional biological processes. An improvement in overall survival is, therefore, one of the most robust endpoints for assessing treatment benefit. Tisagenlecleucel and dinutuximab beta appear to offer the best chance of sustained response to finite treatment, but, in the case of tisagenlecleucel, longer-term data are needed to confirm the curative effect, and our study might not have captured such data. Nusinersen is the only other therapy that reported overall survival. However, for many orphan therapies, outcomes such as overall survival may not be achievable in clinical trials, given the challenges of the patient population and trial design described above. Instead, investigators may look for surrogate outcomes with proven or potential clinical relevance, such as biological changes (for example, tumour response or laboratory measures) or the achievement of functional milestones [47]. In some cases, in the absence of quantitatively measurable endpoints that can be collected in a feasible timeframe, some trials include qualitative endpoints, such as physicians’ or caregivers’ assessment of improvement. However, these outcomes are necessarily subjective and open to influence by external factors, resulting in limited certainty about the findings. Even so, surrogate or subjective outcomes can be used as part of a staged assessment of a therapy’s benefit, contributing to cumulative knowledge and increasing certainty over time [45].

In relation to the above, it is appropriate and important to consider granting conditional marketing authorisation or authorisation under exceptional circumstances for orphan therapies once they show some evidence of benefit, while data continue to be accumulated from ongoing clinical experience to increase confidence in the effects and to potentially provide more accurate guidance on the best use of the therapy [11, 12]. From a payer’s point of view, real world data collection can also help estimate the health economic impact of an orphan therapy where the duration of treatment is unknown. Therapies such as dinutuximab beta and tisagenlecleucel with fixed dosing schedules are associated with greater certainty around the health economic impact than orphan therapies with unlimited treatment duration included in this analysis.

## Limitations of this study

Our study has several limitations. We selected the ICER Matrix to structure our assessment following consideration of a range of tools, but a systematic review of available tools was not undertaken. The Institute for Clinical and Economic Review recently proposed a different approach to assessing the comparative clinical effectiveness of treatments of ultra‐rare diseases [48]. However, following extensive public consultations the ICER Matrix was deemed appropriate for these conditions as well, albeit with an emphasis on the broader aspects of value of ultra‐rare diseases that reflect society’s broader goals [48]. We acknowledge this compromise and that there may be further tools, that we did not identify, which might also be useful in this context.

Implementing the ICER Matrix was often challenging as we did not have a systematic way of assessing all aspects of evidence for a therapy based on clear criteria. The ICER Matrix is a heuristic that allowed us to combine various aspects of treatment benefit and certainty, but even when trying to be as objective as possible, our assessment remains very subjective. We could not even universally apply the hierarchy of evidence stating that RCTs have greater strength than observational studies in principle, recognising that small RCTs can be considerably biased, while large observational studies or single arm studies with historical controls can be more robust. Ultimately, all assessments are subjective, but we have tried to make our assessments as transparent as possible by providing the detailed data extraction (Additional file 3: Appendix 3) and the narrative summaries (Additional file 4: Appendix 4). We also used four reviewers to minimise bias in our assessments, captured the inter-rater agreement across the reviewers, and reached consensus for overall rating.

Our rating focused on clinical benefit, but we acknowledge that value assessments of orphan therapies may also take into consideration other factors that we did not include. These other factors might include patient needs, the impact on families and carers, ethical aspects, the lack of alternative therapies, a broader societal perspective, and specific benefits in terms of costs to health and social care [18, 19]. Also, accounting for broader aspects of uncertainty, as attempted in TRUST and TRUST4RD, might be more informative than tools focusing on certainty of evidence alone [35, 36].

In selecting our sample of orphan therapies, we made every effort to be comprehensive and representative by choosing a major regulatory agency (the EMA) and selecting systematically all the eligible therapies approved within a specific period. We acknowledge that it is possible that other therapies, such as gene therapies, assessed by other agencies within the same period, could have posed different challenges. We have only considered positive decisions made by the EMA. We did not consider the evidence for therapies that the EMA rejected. Nor have we been able to compare European decisions to those made by other regulatory bodies, which may have used different criteria to inform their decisions and may have reached different conclusions.

In terms of evidence identification, we are confident that we have conducted a sensitive search to identify the available key evidence on the eleven therapies, however there is always the chance that we may have missed relevant studies. We have assumed that the EMA would have included relevant evidence published two or more years prior to an EMA decision in the EPAR. Also, our search included evidence published at least one year after EMA decisions, allowing the inclusion of updated results. We acknowledge that this allowed for the inclusion of more evidence (with longer follow up) for older treatments. We also note that new evidence typically emerges after a treatment has been approved (particularly if agency approval is conditional and an agency has requested more evidence) and this evidence might not have contributed to the current ratings.

Some of our evidence was derived from conference abstracts which are sometimes problematic sources of evidence, because they may contain errors, lack detail, provide interim and potentially unchecked data and have not been peer reviewed. We have considered data from conference abstracts as part of the totality of evidence, particularly when evaluated alongside published data. Where data were only available from conference abstracts, we downgraded the certainty of reported treatment effect.

We note the lack of standardisation that can occur in study design terminology. For example, sometimes authors describe a case series in a publication as an observational study. This made deciding on the eligibility of some studies for this analysis challenging.

## Conclusions

We have established that it is feasible to use the ICER Matrix to compare diverse orphan therapies across diseases. The ICER Matrix can facilitate exploration of various aspects of the magnitude of health benefit (NHB) and certainty around the effect estimate. Both NHB and certainty varied considerably across the therapies we evaluated as did the overall rating. Our findings could inform requests from regulatory and HTA bodies for evidence generation (in conjunction with conditional approvals) by helping to identify evidence gaps and uncertainties. As shown by several of the paediatric orphan therapies that we reviewed, consistent, cumulative evidence from carefully designed trials, combined with real-world experience and a good understanding of the natural history of the disease, can provide a level of certainty sufficient to support the use of these treatments in routine clinical practice.

## Supplementary Information

**Additional file 1: Appendix 1.** Search strategies.

**Additional file 2: Appendix 2.** Example rating assessment template.

**Additional file 3: Appendix 3.** Data extraction tables.

**Additional file 4: Appendix 4.** Narrative summaries of the evidence.

## Data Availability

The searches undertaken are in Additional file 1: Appendix 1. The data extraction forms are in Additional file 3: Appendix 3 and narrative summaries of the assessment process are in Additional file 4: Appendix 4.

## Abbreviations

ACCP: American College of Chest Physicians
ALL: Acute lymphoblastic leukaemia
BSA: Body surface area
CAR-T: Chimeric antigen receptor T-cell
CLN2: Type 2 neuronal ceroidolipofuscinosis
DS: Dravet syndrome
EMA: European Medicines Agency
EPAR: European Public Assessment Report
ERT: Enzyme replacement therapy
ESMO: European Society of Medical Oncology
GAG: Glycosaminoglycan
GRADE: Grading of Recommendations, Assessment, Development and Evaluations
HbA1c: Glycosylated haemoglobin
HRNB: High-risk neuroblastoma
HTA: Health technology assessment
ICER: Institute for Clinical and Economic Review
LD: Lipodystrophy
LGS: Lennox–Gastaut syndrome
LY: Life years
MCBS: Magnitude of Clinical Benefit Scale
MCID: Minimal clinically important difference
NHB: Net health benefit
PICOS: Population, intervention, comparator, outcome, study design
QALY: Quality-adjusted life years
QTWIST: Quality-adjusted Time Without Symptoms of disease or Toxicity
RCT: Randomised controlled trial
RRNB: Relapsed/refractory neuroblastoma
SMA: Spinal muscular atrophy
