# Supplementary material for: Exploring the feasibility of using the ICER Evidence Rating Matrix for Comparative Clinical Effectiveness in assessing treatment benefit and certainty in the clinical evidence on orphan therapies for paediatric indications
Source: Orphanet J Rare Dis. 2023 Jul 20;18:193. doi: 10.1186/s13023-023-02701-w (PMC10360248; doi:10.1186/s13023-023-02701-w)
Supplement: Supplementary file 1 — Additional file 1: Appendix 1. Search strategies. [file 13023_2023_2701_MOESM1_ESM.docx]

Appendix 1: Search terms

The following search terms were used to search Embase.com for each of the therapies.

Burosumab

(**'crysvita'**/exp OR **crysvita** OR **'burosumab'**/exp OR **burosumab**) AND (**hypophosphatemi*** OR **hypophosphataemi*** OR **'rickets'**/exp OR **rickets** OR **'osteomalacia'**/exp OR **osteomalacia**) AND (**'pediatric'**/exp OR **pediatric** OR **'paediatric'**/exp OR **paediatric** OR **'child'**/exp OR **child** OR **'childhood'**/exp OR **childhood** OR **'children'**/exp OR **children**) AND **'x linked'** AND [**1-1-2015**]/sd NOT [**1-5-2021**]/sd

Cerliponase alfa

(**'brineura'**/exp OR **brineura** OR **cerliponase** OR **'tripeptidyl peptidase i'**/exp OR **'tripeptidyl peptidase i'**) AND (**'neuronal ceroid lipofuscinosis'**/exp OR **'neuronal ceroid lipofuscinosis'** OR (**neuronal** AND (**'ceroid'**/exp OR **ceroid**) AND (**'lipofuscinosis'**/exp OR **lipofuscinosis**)) OR **cln2** OR **'tripeptidyl peptidase'**/exp OR **'tripeptidyl peptidase'** OR (**tripeptidyl** AND (**'peptidase'**/exp OR **peptidase**)) OR **'juvenile neuronal ceroid lipofuscinosis'**/exp OR **'juvenile neuronal ceroid lipofuscinosis'**) AND (**'pediatric'**/exp OR **pediatric** OR **'paediatric'**/exp OR **paediatric** OR **'child'**/exp OR **child** OR **'childhood'**/exp OR **childhood** OR **'children'**/exp OR **children**) AND [**1-1-2015**]/sd NOT [**1-5-2021**]/sd

CDCA

(**'chenodeoxycholic acid'**/exp OR **'chenodeoxycholic acid'**) AND (**'sterol 27 hydroxylase deficiency'** OR ((**'sterol'**/exp OR **sterol**) AND **27** AND (**'hydroxylase'**/exp OR **hydroxylase**) AND (**'deficiency'**/exp OR **deficiency**)) OR **'cerebrotendinous xanthomatosis'**/exp OR **'cerebrotendinous xanthomatosis'**) AND (**'pediatric'**/exp OR **pediatric** OR **'paediatric'**/exp OR **paediatric** OR **'child'**/exp OR **child** OR **'childhood'**/exp OR **childhood** OR **'children'**/exp OR **children**) AND [**1-1-2015**]/sd NOT [**1-5-2021**]/sd

Dinutuximab beta

**('qarziba'**/exp OR **qarziba** OR **'dinutuximab beta'**/exp OR **'dinutuximab beta'** OR ((**'dinutuximab'**/exp OR **dinutuximab**) AND **beta**)) AND (**'neuroblastoma'**/exp OR **neuroblastoma**) AND (**'pediatric'**/exp OR **pediatric** OR **'paediatric'**/exp OR **paediatric** OR **'child'**/exp OR **child** OR **'childhood'**/exp OR **childhood** OR **'children'**/exp OR **children**) AND [**1-1-2015**]/sd NOT [**1-5-2021**]/sd

Epidiolex

(**'epidiolex'**/exp OR **epidiolex** OR **'cannabidiol'**/exp OR **cannabidiol**) AND (**'lennox gastaut'**/exp OR **'lennox gastaut'** OR **'severe myoclonic epilepsy in infancy'**/exp OR **'severe myoclonic epilepsy in infancy'**) AND (**'pediatric'**/exp OR **pediatric** OR **'paediatric'**/exp OR **paediatric** OR **'child'**/exp OR **child** OR **'childhood'**/exp OR **childhood** OR **'children'**/exp OR **children**) AND [**1-1-2015**]/sd NOT [**1-5-2021**]/sd

Glibenclamide

(**'amglidia'**/exp OR **amglidia** OR **'glibenclamide'**/exp OR **glibenclamide**) AND (**'neonatal diabetes'**/exp OR **'neonatal diabetes'** OR (**neonatal** AND (**'diabetes'**/exp OR **diabetes**))) AND (**'pediatric'**/exp OR **pediatric** OR **'paediatric'**/exp OR **paediatric** OR **'child'**/exp OR **child** OR **'childhood'**/exp OR **childhood** OR **'children'**/exp OR **children**) AND [**1-1-2015**]/sd NOT [**1-5-2021**]/sd

Metreleptin

(**'myalepta'**/exp OR **myalepta** OR **'myalept'**/exp OR **myalept** OR **'metreleptin'**/exp OR **metreleptin**) AND (**'leptin** **deficiency'**/exp OR **'leptin** **deficiency'** OR ((**'leptin'**/exp OR **leptin**) AND (**'deficiency'**/exp OR **deficiency**)) OR **'lipodystrophy'**/exp OR **lipodystrophy** OR **'berardinelli-seip** **syndrome'**/exp OR **'berardinelli-seip syndrome'** OR (**'berardinelli seip'** AND (**'syndrome'**/exp OR **syndrome**)) OR **'lawrence syndrome'** OR (**lawrence** AND (**'syndrome'**/exp OR **syndrome**))) AND (**'pediatric'**/exp OR **pediatric** OR **'paediatric'**/exp OR **paediatric** OR **'child'**/exp OR **child** OR **'childhood'**/exp OR **childhood** OR **'children'**/exp OR **children**) AND [**1-1-2015**]/sd NOT [**1-5-2021**]/sd

Nusinersen

(**'spinraza'**/exp OR **spinraza** OR **'nusinersen'**/exp OR **nusinersen**) AND (**'spinal muscular atrophy'**/exp OR **'spinal muscular atrophy'** OR (**spinal** AND **muscular** AND (**'atrophy'**/exp OR **atrophy**)) OR **sma**) AND (**'pediatric'**/exp OR **pediatric** OR **'paediatric'**/exp OR **paediatric** OR **'child'**/exp OR **child** OR **'childhood'**/exp OR **childhood** OR **'children'**/exp OR **children**) AND [**1-1-2015**]/sd NOT [**1-5-2021**]/sd

Tisagenlecleucel

(**'kymriah'**/exp OR **kymriah** OR **'tisagenlecleucel'**/exp OR **tisagenlecleucel**) AND ((**'b-cell lymphoblastic'** OR ((**'b cell'**/exp OR **'b cell'**) AND **lymphoblastic**)) AND (**'leukemia'**/exp OR **leukemia**) OR **'leukaemia'**/exp OR **leukaemia**) AND (**'pediatric'**/exp OR **pediatric** OR **'paediatric'**/exp OR **paediatric** OR **'child'**/exp OR **child** OR **'childhood'**/exp OR **childhood** OR **'children'**/exp OR **children**) AND [**1-1-2015**]/sd NOT [**1-5-2021**]/sd

Velmanase alfa

**('lamzede'**/exp OR **lamzede** OR **'velmanase alfa'**/exp OR **'velmanase alfa'**) AND (**'mannosidosis'**/exp OR **mannosidosis**) AND (**'pediatric'**/exp OR **pediatric** OR **'paediatric'**/exp OR **paediatric** OR **'child'**/exp OR **child** OR **'childhood'**/exp OR **childhood** OR **'children'**/exp OR **children**) AND [**1-1-2015**]/sd NOT [**1-5-2021**]/sd

Vestronidase alfa

(**'mepsevii'**/exp OR **mepsevii** OR **vestronidase** OR **'vestronidase alfa'**/exp OR **'vestronidase alfa'**) AND (**'mucopolysaccharidosis vii'**/exp OR **'mucopolysaccharidosis vii'** OR ((**'mucopolysaccharidosis'**/exp OR **mucopolysaccharidosis**) AND **vii**) OR **'mucopolysaccharidosis type 7'**/exp OR **'mucopolysaccharidosis type 7'**) AND (**'pediatric'**/exp OR **pediatric** OR **'paediatric'**/exp OR **paediatric** OR **'child'**/exp OR **child** OR **'childhood'**/exp OR **childhood** OR **'children'**/exp OR **children**) AND [**1-1-2015**]/sd NOT [**1-5-2021**]/sd

The following conferences were searched for presentations relating to the therapies for the period 2015 onwards:

- Advances in Neuroblastoma Research (ANR)
- International Society of Paediatric Oncology (SIOP)
- Society for Immunotherapy of Cancer (SITC)
- The European Society for Blood and Marrow Transplantation (EBMT)
- American Society of Clinical Oncology (ASCO)
- European Society of Medical Oncology (ESMO)
- American Association for Cancer Research (AACR)
- American Society of Hematology (ASH)
- European Hematology Association (EHA)
- Americal Diabetes Association (ADA)
- European Association for the Study of Diabetes (EASD)
- International Society for Pediatric and Adolescent Diabetes (ISPAD)
- International Diabetes Federation (IDF)
- Endocrine Society (ENDO)
- European Society for Paediatric Endocrinology (ESPE)
- European Congress of Endocrinology (ECE)
- International Congress of Inborn Errors of Metabolism (ICIEM)
- Society for the Study of Inborn Errors of Metabolism (SSIEM)
- International Society for Mannosidosis & Related Diseases (ISMRD)
- World Congress (WORLD)
- American Academy of Neurology (AAN)
- American Neurological Association (ANA)
- World Congress of Nephrology (WCN)
- European Academy of Neurology (EAN)
- European Paediatric Neurology Society (EPNS)
- Child Neurological Society (CNS)
- International Congress on Neuromuscular Diseases (ICNMD)
- World Muscle Society (WMS)
- International Parkinson and Movement Disorder Society (IPMDS)
- International Pediatric Nephrology Association (IPNA)
- International Conference on Children's Bone Health (ICCBH)
- World Congress on Osteoporosis, Osteoarthritis and Musculoskeletal Diseases (WCO-IOF-ESCEO)
- American Society for Bone and Mineral Research (ASBMR)
- European Congress of Endocrinology (ECE)
- European Calcified Tissue Society (ECTS)
- International Epilepsy Congress (IEC)
- European Congress of Endocrinology (ECE)
- American Epilepsy Society (AES)
- International Society for Pharmacoeconomics and Outcomes Research (ISPOR)
- European Conference on Rare Disease (ECRD)
