# Supplementary material for: Exploring the feasibility of using the ICER Evidence Rating Matrix for Comparative Clinical Effectiveness in assessing treatment benefit and certainty in the clinical evidence on orphan therapies for paediatric indications
Source: Orphanet J Rare Dis. 2023 Jul 20;18:193. doi: 10.1186/s13023-023-02701-w (PMC10360248; doi:10.1186/s13023-023-02701-w)
Supplement: Supplementary file 2 — Additional file 2: Appendix 2. Example rating assessment template. [file 13023_2023_2701_MOESM2_ESM.docx]

**Appendix 2: Example rating assessment table**

| **Therapy** | **BU** | **CA** |  | **CD** | **CE** | **DB** |  | **GL** | **ME** |  | **NU** |  |  | **TI** | **VL** | **VS** |  |
| --- | --- | --- | --- | --- | --- | --- | --- | --- | --- | --- | --- | --- | --- | --- | --- | --- | --- |
| **Indication** |  | **DS** | **LGS** |  |  | **MT** | **RR** |  | **GN** | **PR** | **T1** | **T2/3** | **PS** |  |  |  |  |
| **Net Health Benefit** | | | | | | | | | | | | | | | | | |
| **Clinical relevance of effect size** | **2** | **3** | **3** | **2** | **2** | **3** | **3** | **2** | **3** | **2** | **3** | **3** | **2** | **3** | **2** | **1** | **39** |
| **Clinical relevance of efficacy endpoints** | **2** | **2** | **2** | **1** | **3** | **3** | **3** | **3** | **2** | **2** | **3** | **2** | **2** | **3** | **2** | **2** | **37** |
| **Impact on QoL** | **2** | **2** | **2** | **2** | **2** | **2** | **2** | **1** | **1** | **1** | **2** | **2** | **1** | **2** | **2** | **2** | **28** |
| **Clinical relevance of adverse events** | **2** | **2** | **2** | **1** | **2** | **1** | **1** | **3** | **2** | **1** | **2** | **2** | **1** | **1** | **2** | **2** | **27** |
| **Potential curative effect** | **2** | **2** | **2** | **1** | **1** | **3** | **2** | **1** | **1** | **1** | **3** | **3** | **2** | **2** | **1** | **1** | **28** |
| **Final assessment*** | **S** | **SU** | **SU** | **N** | **S** | **SU** | **S** | **S** | **S** | **C** | **SU** | **S** | **C** | **SU** | **C** | **N** | **159** |
| **Coded** | **2** | **3** | **3** | **2** | **2** | **4** | **4** | **2** | **2** | **3** | **4** | **4** | **3** | **3** | **3** | **3** |  |
|  | | | | | | | | | | | | | | | | | |
| **Certainty** | | | | | | | | | | | | | | | | | |
| **POPULATION** | | | | | | | | | | | | | | | | | |
| **Number of patients studied** | **2** | **2** | **2** | **2** | **2** | **2** | **2** | **2** | **2** | **1** | **2** | **2** | **2** | **2** | **1** | **1** | **29** |
| **Eligibility criteria** |  |  |  |  |  |  |  |  |  |  |  |  |  |  |  |  |  |
| **INTERVENTION** | | | | | | | | | | | | | | | | | |
| **Concomitant treatments** | **2** | **2** | **1** | **1** | **2** | **2** | **3** | **3** | **1** | **1** | **2** | **2** | **2** | **2** | **1** | **1** | **28** |
| **Duration of treatment** |  |  |  |  |  |  |  |  |  |  |  |  |  |  |  |  |  |
| **COMPARATOR** | | | | | | | | | | | | | | | | | |
| **Comparator consistency** | **3** | **3** | **3** | **1** | **2** | **3** | **3** | **3** | **1** | **1** | **2** | **1** | **1** | **2** | **1** | **1** | **31** |
| **Comparator relevance** |  |  |  |  |  |  |  |  |  |  |  |  |  |  |  |  |  |
| **OUTCOMES** | | | | | | | | | | | | | | | | | |
| **Variation of outcomes between studies** | **2** | **2** | **2** | **2** | **2** | **3** | **3** | **2** | **2** | **1** | **2** | **2** | **2** | **2** | **3** | **3** | **35** |
| **Statistical significance of outcomes** |  |  |  |  |  |  |  |  |  |  |  |  |  |  |  |  |  |
| **STUDY DESIGN** | | | | | | | | | | | | | | | | | |
| **Types of clinical studies** | **2** | **2** | **2** | **2** | **2** | **2** | **2** | **2** | **2** | **2** | **2** | **2** | **2** | **2** | **2** | **2** | **32** |
| **Duration of studies** |  |  |  |  |  |  |  |  |  |  |  |  |  |  |  |  |  |
| **Final assessment**** | **HM** | **HM** | **HM** | **LM** | **HM** | **H** | **H** | **HM** | **M** | **L** | **HM** | **M** | **LM** | **HM** | **L** | **L** | **155** |
| **Coded** | **3** | **3** | **3** | **1** | **3** | **5** | **3** | **3** | **4** | **2** | **5** | **4** | **4** | **3** | **3** | **1** |  |

**Key to therapies and indications**

| **Abbreviation** | **Therapy** | **Indications (sub-populations)** |
| --- | --- | --- |
| BU | burosumab | X-linked hypophosphatemia |
| CA | cannabidiol | 1. Dravet syndrome (DS) 2. Lennox–Gastaut syndrome (LGS) |
| CD | chenodeoxycholic acid | inborn errors of primary bile acid synthesis |
| CE | cerliponase alfa | neuronal ceroid lipofuscinosis 2 disease |
| DB | dinutuximab beta | 1. high-risk maintenance neuroblastoma (MT) 2. relapsed/refractory neuroblastoma (RR) |
| GL | glibenclamide | neonatal diabetes mellitus |
| ME | metreleptin | 1. generalised lipodystrophy (GN) 2. partial lipodystrophy (PR) |
| NU | nusinersen | 1. Type I spinal muscular atrophy (T1) 2. Type II/III spinal muscular atrophy (T2/3) 3. presymptomatic spinal muscular atrophy (PS) |
| TI | tisagenlecleucel | relapsed/refractory B-cell acute lymphoblastic leukaemia |
| VL | velmanase alfa | mild to moderate alpha-mannosidosis |
| VS | vestronidase alfa | mucopolysaccharidosis VII |

Weights from 1 to 3 were assigned to each factor to reflect its contribution to the overall assessment, specifically its impact on the assessment grade (not the magnitude or certainty of actual benefit of treatment). The weights were converted to percentages so that the results could be compared.
