# Supplementary material for: Exploring the feasibility of using the ICER Evidence Rating Matrix for Comparative Clinical Effectiveness in assessing treatment benefit and certainty in the clinical evidence on orphan therapies for paediatric indications
Source: Orphanet J Rare Dis. 2023 Jul 20;18:193. doi: 10.1186/s13023-023-02701-w (PMC10360248; doi:10.1186/s13023-023-02701-w)
Supplement: Supplementary file 3 — Additional file 3: Appendix 3. Data extraction tables. [file 13023_2023_2701_MOESM3_ESM.docx]

**Appendix 3 – data extraction tables**

**Contents**

[Burosumab](#Burosumab)

[Cannabidiol](#Cannabidiol)

[CDCA](#CDCA)

[Cerliponase alfa](#Cerliponase)

[Dinutuximab beta](#Dinutuximab)

[Glibenclamide](#Glibenclamide)

[Metreleptin](#Metreleptin)

[Nusinersen](#Nusinersen)

[Tisagenlecleucel](#Tisagenlecleucel)

[Velmanase alfa](#Velmanase)

[Vestronidase alfa](#Vestronidase)

[References](#References)

**Evidence rating criteria**

Drugs are rated according to the level of benefit that they offer in a specific indication.

A = substantial benefit vs. risk

B = small benefit vs. risk

C = similar benefit vs. risk

I = inconclusive findings

+ = relative certainty in the results

**Abbreviations used.**

3MSCT, 3-minute stair climb test

6MWT, 6-minute walk test

AE, adverse event

ALL, acute lymphoblastic leukaemia

BID, twice daily

BSA, body surface area

CAR-T, chimeric antigen receptor T-cell

CBD, cannabidiol

CGICSD, Caregiver Global Impression of Change in Seizure Duration

CHAQ, Childhood Health Assessment Questionnaire

CI, confidence interval

CR, complete remission

CRi, complete remission with incomplete blood count recovery

DS, Dravet syndrome

EFS, event-free survival

EQ-5D, EuroQol 5-dimension questionnaire

FVC, forced vital capacity

GAG, glycosaminoglycan

HbA1c, glycated haemoglobin

HFMSE, Hammersmith Functional Motor Scale–Expanded

HINE, Hammersmith Infant Neurological Examination

HR, hazard ratio

HRNB, high-risk neuroblastoma

HSCT, haematopoietic stem-cell transplantation

IL-2, interleukin-2

IQR, interquartile range

IV, intravenous

LD, lipodystrophy

LGS, Lennox-Gastaut syndrome

LSM, least-squares mean

MCID, minimal clinically important difference

MDRI, multi-domain responder index

NA, not available

NS, not significant

OR, odds ratio

OS, overall survival

PedsQL, Pediatric Quality of Life Inventory

POSNA-PODCI, Paediatric Orthopaedic Society of North America – Paediatric Outcomes Data Collection Instrument

QoL, quality of life

r/r, relapsed/refractory

RRNB, relapse or refractory neuroblastoma

SAE, serious adverse event

SC, subcutaneous

SCT, stem-cell therapy

SMA, spinal muscular atrophy

TEAE, treatment-emergent adverse event

TGs, triglycerides

VAS, visual analogue scale

XLH, X-linked hypophosphataemia

| Treatment | Burosumab | | | |
| --- | --- | --- | --- | --- |
| Evidence rating | B+ | | | |
| Population | ***Indication***  Adult and paediatric (1 year and older) patients with X-linked hypophosphatemia | | ***Incidence/eligible population***  Incidence: 0.5/100,000 newborns [1] | |
| Intervention | **Dose**  0.8 mg/kg body weight, rounded to nearest 10 mg  Maximum dose: 90 mg | **Frequency**  Every 2 weeks | **Duration**  Long-term (in some markets until end of growth phase) | **Stopping criteria**  Adverse events, lack of efficacy, in some markets end of growth phase |
| Comparator | Oral phosphates and active vitamin D analogues (‘conventional therapy’) | | | |
| Outcomes | **Study CL301[2-4]**   \| Primary outcome—Rickets severity \| *LSM Radiographic Global Impression of Change score at week 40:*  Burosumab: +1.9  Conventional therapy: +0.8  Difference: 1.1 (95% CI: 0.8, 1.5), p<0.0001  *LSM Radiographic Global Impression of Change score at week 64 (post hoc analysis based on oral phosphate and active vitamin D (Pi/D) dose categories)*:  The LS mean RGI‐C score with burosumab was greater (2.1 ± 0.1) vs. on‐study treatment with phosphate >40 mg/kg (HPi) (1.0 ± 0.2), phosphate <=40 mg/kg (LPi) (1.0 ± 0.2), alfacalcidol >60 ng/kg or calcitriol >30 ng/kg (HD) (1.5 ± 0.2), or alfacalcidol >60 ng/kg or calcitriol >30 ng/kg (LD) (0.7 ± 0.2). The LS mean improvement in lower limb deformity RGI‐C with burosumab (+1.3 ± 0.2) was greater vs. HPi (+0.3 ± 0.2), LPi (+0.3 ± 0.1), HD (+0.4 ± 0.2), or LD (+0.2 ± 0.1). \| \| --- \| --- \| \| Key secondary outcome—Growth \| Change from baseline in recumbent length and standing height Z score: 0.14 (95% CI: 0, 0.29) \| \| Key secondary outcome—Motor function \| Change from baseline in mean percent predicted 6-minute walk test: 7% (95% CI: 0.01, 14.52) \| \| Key secondary outcome – Fasting serum phosphorus concentration \| Burosumab versus conventional therapy:  At Week 40: LS mean change ± SE: 0·29 ± 0·03  versus 0·06 ± 0·02 mmol/L [0·92 ± 0·08 versus 0·20 ± 0·06 mg/dL]; p<0·0001.  At Week 64: 0·29 ± 0·03 versus 0·07 ± 0·02 mmol/L [0·91 ± 0·08 versus 0·21 ± 0·06 mg/dL]; p<0·0001. \| \| Outcome of interest—OS \| NR \| \| Outcome of interest—QoL \| Results only show a clinically meaningful significant between group difference for one of the outcomes: PROMIS Pain Interference score (but only at week 40) There was a significant within group difference for SF-10 physical health scores (i.e. change from baseline in the burosumab group).  *PROMIS Pain Interference score at weeks 40 and 64:*  Burosumab: least-squares mean [SE] change: -5.31 (1.705)* at week 40 and -3.55 (1.873)* at week 64  Conventional therapy: -0.29 (1.539) at week 40 and -1.29 (1.267) at week 64  Difference: -5.02 (95% CI: -9.29, -0.75), p=0.0212 at week 40 and -2.26 (95% CI: -6.61, 2.09), p=0.3091 at week 64  *PROMIS Physical Function Mobility at weeks 40 and 64:*  Burosumab: LS mean (SE): + 2.78 (1.336) at week 40 and + 2.82 (1.648) at week 64  Conventional therapy: + 0.10 (0.966) at week 40 and + 0.92 (0.962) at week 64  Difference: + 2.68 (95% CI: -0.52, 5.89), p=0.1009 at week 40 and + 1.90 (95% CI: -1.80, 5.59), p=0.3145 at week 64  *PROMIS Fatigue at weeks 40 and 64;*  Burosumab: LS mean (SE) -4.29 (1.709)* at week 40 and -3.65 (2.119)* at week 64  Conventional therapy: LS mean (SE) -1.05 (1.754) at week 40 and  -2.57 (1.547) at week 64  Difference: -3.25 (95% CI: -7.86, 1.37), p=0.1676 at week 40 and  -1.08 (95% CI: − 6.21, 4.06), p=0.6810 at week 64  *SF-10 Health Survey for Children at weeks 40 and 64 (physical health scores):*  Burosumab LS mean (SE) scores from baseline were + 5.98 (1.79), p=0.0008 at week 40 and + 5.93 (1.88), p=0.0016 at week 64  Conventional therapy: + 1.65 (2.17) at week 40 and + 0.44 (2.22) at week 64  Difference: + 4.33 (2.82) at week 40 and + 5.49 (2.91) at week 64 (p values not significant [not reported])  *Clinically meaningful difference \| \| Safety \| Severe (grade 3/4) AEs: <30% for both groups (burosumab 13.8%, conventional therapy 9.4%) \|   **Study CL201[1, 5]**   \| Primary outcome—Rickets severity \| *Change in Thacher rickets severity total score at week 40:*  Burosumab 2 weekly: -1.1, p<0.01  Burosumab 4 weekly: -0.7, p<0.01 \| \| --- \| --- \| \| Outcome of interest—OS \| NR \| \| Outcome of interest—QoL \| *POSNA-PODCI questionnaire:*  Improved functional ability and decreased pain at Week 64 \| \| Safety \| Severe (grade 3/4) AEs:<30% (4%) \|   **Study CL205[2, 6]**   \| Co-primary outcome—Safety \| Severe (grade 3/4) AEs:<30% (8%)  No new safety concerns emerged by 160 weeks \| \| --- \| --- \| \| Co-primary outcome—Fasting serum phosphorus concentration \| *LSM change from baseline to week 40:* 0.31 mmol/L, p<0.001  *Absolute concentration:*  Baseline:2.5 (SD 0.3)  Week 64: 3.4 (SD 0.5)  Week 160: 3.4 (SD 0.5) (p value not reported) \| \| Key secondary outcome—Rickets severity \| *Total Thacher rickets severity score, change from baseline to week 64:*  LSM decrease: –2.0  *Absolute scores:*  Baseline: 2.9 (SD 1.4)  Week 64: 0.9 (SD 0.5)  Week 160: 1.0 SD (0.6) (p value not reported) \| \| Outcome of interest – Radiographic Global Impression of Change \| *Radiographic Global Impression of Change at week 64:*  LSM: +2.2 (improvement), and +2,2 at week 160 \| \| Outcome of interest—OS \| NR \| \| Outcome of interest—QoL \| NR \|   **Israeli before-and-after study [7]**   \| Rickets severity (primary and secondary outcomes not stated) \| *Change in Thacher rickets severity total score:*  Baseline: 2.29 (SD 1.07)  12 months: 0.43 (SD 0.73), p=0.005 \| \| --- \| --- \| \| Growth \| *Change from baseline in length (cm):*  Baseline: 121.3 (SD 16.3)  6 months: 125.4 (SD 16.3), p<0.001  12 months: 128.3 (SD 5.4), p<0.001 \| \| Outcome of interest—OS \| NR \| \| Outcome of interest—QoL \| NR \| \| Safety \| NR \| | | | |
| Study designs | \| Trial \| Patients \| Design \| Follow-up \| \| --- \| --- \| --- \| --- \| \| Study CL301 (NCT02915705) \| N=61 \| Multicentre, phase III randomised, open-label \| 64 weeks \| \| Study CL201 (NCT02163577) \| N=52 \| Multicentre, phase II randomised, open-label, dose-finding \| 64 weeks (plus long-term extension) \| \| Study CL205 (NCT02750618) \| N=13 (n=12 at 160 weeks) \| Multicentre, phase II single-arm, open-label \| 64 and 160 weeks \| \| Israel before-and-after study \| N=7 \| Single centre before- and-after \| 12 months \| | | | |

| Treatment | Cannabidiol | | | |
| --- | --- | --- | --- | --- |
| Evidence rating | B+ (Lennox-Gastaut Syndrome (LGS)), B+ (Dravet Syndrome (DS)) | | | |
| Population | ***Indication***  Patients ≥2 years with seizures associated with LGS or DS, in conjunction with clobazam. | | ***Incidence/eligible population***  Incidence: 2/100,000 children (LGS) [8]  2–6/100,000 births (DS) [8-10]  Prevalence: 15/100,000 total population (LGS),[8]  2–3/100,000 total population (DS) [10, 11] | |
| Intervention | **Dose**  Starting dosage: 2.5 mg/kg BID for 1 week  After 1 week, 5 mg/kg BID maintenance dose (CBD10)  Further increases individualised for clinical response/tolerability: weekly increments of 2.5 mg/kg BID up to maximum 10 mg/kg BID (CBD20)  Dose should be down-titrated before stopping | **Frequency**  Twice daily | **Duration**  Ongoing | **Stopping criteria**  Lack of efficacy, adverse events (e.g. liver dysfunction), loss of seizure control |
| Comparator | Combination therapy based on clobazam, stiripentol, sodium valproate, topiramate and/or levetiracetam | | | |
| Outcomes | **Dravet Syndrome**  **GWPCARE1 [12] [11]**   \| Primary outcome—Convulsive seizure frequency^[[1]](#footnote-1)^ \| *Median change from baseline*  Cannabidiol −38.9% (IQR −69.5 to −4.8)  Placebo −13.3% (IQR −52.5 to 20.2)  Adjusted median difference: −22.8 percentage points (95% CI: −41.1, −5.4), p=0.01.  43% of patients had at least a 50% reduction in convulsive seizure frequency with cannabidiol and 27% with placebo (odds ratio, 2.00; 95% CI, 0.93 to 4.30; p=0.08). \| \| --- \| --- \| \| Key secondary outcome—Caregiver Global Impression of Change \| The patient’s overall condition improved by at least one category in 62% of the cannabidiol group compared with 34% of the placebo group (p=0.02). Median difference vs. placebo in change from baseline: −1.0 (95% CI: −1.0, 0.0), p=0.02. \| \| Outcome of interest—OS \| NR \| \| Outcome of interest—QoL \| *Sleep disruption score (NRS scale) change from baseline:*  −0.4 (95% CI: −1.5, 0.7), p=0.45  *Epworth Sleepiness Scale score change from baseline:*  1.5 (95% CI: −0.2, 3.2), p=0.08  *Quality of Life in Childhood Epilepsy score change from baseline:*  1.5 (95% CI: −3.8, 6.8), p=0.58  *Vineland-II score change from baseline:*  −2.6 (95% CI: −6.8, 1.6), p=0.21 \| \| Safety \| Severe (grade 3/4) AEs: <30% (cannabidiol 16%, placebo 5%) \|   **GWPCARE2 [13]**   \| Primary outcome—Convulsive seizure frequency \| The percentage reduction from baseline in convulsive seizure  frequency was 48.7% for CBD10 group and 45.7% for the CBD20 group vs. 26.9% for the placebo group.  The percentage reduction from placebo was 29.8% (95%CI, 8.4%-46.2%; p=0.01) for CBD10 group and 25.7% (95%CI, 2.9%-43.2%; p =0.03) for the CBD20 group.  The proportion of patients achieving at least 50% reduction from baseline during the treatment period was 43.9% (n = 29) for the CBD10 group (P=0.03), 49.3% (n = 33) for the CBD20 group (P=0.007), and 26.2% (n = 17) for the placebo group. \| \| --- \| --- \| \| Key secondary outcome—Caregiver Global Impression of Change \| CBD10 vs. placebo: OR=2.93 (95% CI: 1.56, 5.53), p=0.0009  CBD20 vs. placebo: OR=2.02 (95% CI: 1.08, 3.78), p=0.0279  Very much or much improved:  CBD10: 21/66 (32%)  CBD20: 24/66 (36%)  Placebo: 9/65 (14%) \| \| Outcome of interest—OS \| NR \| \| Outcome of interest—QoL \| *Sleep disruption score (NRS scale) change from baseline:*  CBD10 0 (95% CI: –0.9, 0.8)  CBD20 –0.1 (95% CI: –0.9, 0.8)  *Epworth Sleepiness Scale score change from baseline:*  CBD10 –0.55 (95% CI: –1.86, 0.75)  CBD20 0.74 (95% CI: –0.57, 2.05)  *Quality of Life in Childhood Epilepsy score change from baseline:*  CBD10 3.8 (95% CI: –0.1, 7.8)  CBD20 1.8 (95% CI: –2.2, 5.8)  *Vineland-II score change from baseline:*  CBD10 –0.4 (95% CI: –2.5,1.7)  CBD20 0.0 (95% CI: –2.2, 2.2) \| \| Safety \| Severe (grade 3/4) AEs: <1% \|   **GWPCARE5 [14]**   \| Primary outcome—Safety \| Serious AEs: 41% (severe AEs NR) \| \| --- \| --- \| \| Key secondary outcomes—Seizure frequency \| *Change from baseline in 12-week windows over 156 weeks:*  Convulsive seizures: 45–73%  Total seizures: 49–80% \| \| Outcome of interest—OS \| NR \| \| Outcome of interest—QoL \| NR \|   **Lennox-Gastaut syndrome**  **GWPCARE3 [15]**   \| Primary outcome—Drop seizure frequency \| The median reduction from baseline per 28 days during  the treatment period was 41.9% in the CBD20 group, 37.2% in the CBD10 group, and 17.2% in the placebo group.  *Change from baseline, median difference CBD20 vs. placebo:*  21.6% (95% CI: 6.7, 34.8%), p=0.005  *CBD10 vs. placebo:* 19.2% (95% CI 7.7, 31.2%), p=0.002.  39% patients in the CBD20 group, 36% in the CBD10 group,  and 14% in the placebo group had  at least a 50% reduction from their baseline in  drop-seizure frequency (OR for the CBD20 group vs. the placebo: 3.85; 95% CI, 1.75 to 8.47; p<0.001; OR for CBD10 group vs. the placebo: 3.27; 95% CI, 1.47 to 7.26; p=0.003). \| \| --- \| --- \| \| Key secondary outcome—Caregiver Global Impression of Change \| *Odds ratio, cannabidiol vs. placebo:*  CBD10: 2.57 (95% CI: 1.41, 4.66), p=0.002  CBD20: 1.83 (95% CI: 1.02, 3.30), p=0.04  Very much or much improved:  CBD20: 21/75 (28%)  CBD10: 23/73 (31.5%)  Placebo: 9/75 (12%) \| \| Outcome of interest—OS \| NR \| \| Outcome of interest—QoL \| *Change from baseline in sleep disruption 0–10 numerical rating scale score:*  CBD10 vs. placebo: −0.8 (−1.7 to 0.1)  CBD20 vs. placebo: −0.3 (−1.2 to 0.6)  *Change from baseline in Epworth Sleepiness Scale score*:  CBD10 vs. placebo: 0.09 (−1.38 to 1.56)  CBD20 vs. placebo: 0.01 (−1.46 to 1.47)  *Change from baseline in Quality of Life in Childhood Epilepsy overall quality of life score*  CBD10 vs. placebo: 1.6 (−4.5 to 7.8)  CBD20 vs. placebo: −5.1 (−11.4 to 1.2)  *Change from baseline in Vineland Adaptive Behavior Scales (Second Edition) Adaptive Behavior Composite Standard Score:*  CBD10 vs. placebo: 0.5 (−1.3 to 2.3)  CBD20 vs. placebo: 0.1 (−1.4 to 1.6) \| \| Safety \| Severe (grade 3/4) AEs: <30% (11% including placebo, CBD10 and CBD20 groups) \|   **GWPCARE4 [16]**   \| Primary outcome—Drop seizure frequency \| In the CBD20 group, the monthly frequency of drop  seizures decreased by a median of 43·9% (IQR −69·6 to −1·9)  from baseline over the 14-week treatment period. In the  placebo group, drop seizures decreased by a median of 21·8% (IQR −45·7 to 1·7).  *Change from baseline, median difference between groups:* −17.21 (95% CI: −30.32, −4.09), p=0.0135.  44% of patients in the cannabidiol group had a reduction in drop seizure frequency of 50% or more from baseline during the treatment period compared with 24% in the placebo group (OR 2·57, 95% CI 1·33–4·97) p=0·0043. \| \| --- \| --- \| \| Key secondary outcome— Caregiver Global Impression of Change \| OR 2.54 (95% CI: 1.5, 4.5), p=0.0012  Very much or much improved:  CBD20: 35%  Placebo: 17% \| \| Outcome of interest—OS \| NR \| \| Outcome of interest—QoL \| NR \| \| Safety \| Severe (grade 3/4) AEs: NR \|   **GWPCARE5 [17]**   \| Primary outcome—Safety \| Serious AEs: 42% (severe AEs NR) \| \| --- \| --- \| \| Key secondary outcome—Seizure frequency \| *Median change from baseline, difference vs. placebo at 12-week windows over 156 weeks:*  Convulsive seizure frequency: 48–71%  Total seizure frequency: 48–68% \| \| Outcome of interest—OS \| NR \| \| Outcome of interest—QoL \| NR \|   **Pooled data from GWPCARE 3-4 (post hoc analysis) [18]**   \| Time to onset of CBD treatment effect (seizure reduction and adverse events [AEs]) \| *Reduction in drop seizures:*  Differences between placebo and CBD [10 + 20mg.kg/day] emerged during the titration period and was significant by day 6 (p=0.008).  *Onset of the first reported AE:*  Onset occurred during the titration period in 45% of patients (CBD10, 46%; CBD20, 52%; placebo, 38%). Resolution occurred within 4 weeks of onset in 53% of placebo and 39% of CBD patients; and by end of study, resolution occurred in 63% of placebo and 61% of CBD patients. \| \| --- \| --- \| \| Outcome of interest—OS \| NR \| \| Outcome of interest—QoL \| NR \| \| Safety \| Severe (grade 3/4) AEs: NR \|   **DS and LGS**  **Expanded Access Program [19]**   \| Primary outcome—Safety \| Serious AEs: 41% (severe AEs NR) \| \| --- \| --- \| \| Other outcome—reduction in seizure frequency \| *Proportion of patients with ≥50%, ≥75% and 100% reductions*  Major motor seizures: 53%, 23% and 6%  Total seizures: 46%, 26% and 5% \| \| Outcome of interest—OS \| NR \| \| Outcome of interest—QoL \| NR \|   **Meta-analysis of GWPCARE1-4 [20]**   \| Primary outcome—reduction in seizure frequency \| *Reduction in seizure frequency in patients with DS and LGS* (CBD [10 + 20mg.kg/day] vs. placebo):  Treatment ratio: 0.59 (95% CI: 0.52, 0.68, p<0.0001) with concomitant clobazam (CLB) and 0.85 (95% CI: 0.73, 0.98), p=0.0226) without CLB (both in favour of treatment)  *50% responder rate in patients with DS and LGS* (CBD [10 + 20mg.kg/day] vs. placebo):  Odds ratio: 2.51 (95% CI: 1.69, 3.71, p<0.0001) with CLB and 2.40 (95% CI: 1.38, 4.16, p=0.0020) without CLB (both in favour of treatment) \| \| --- \| --- \| \| Key secondary outcome - proportion of patients with ≥ 50% reduction in seizure frequency (50% responder rate) \| CBD dose vs. placebo: OR 2.51 (95% CI: 1.69, 3.71, p<0.0001) with CLB and 2.40 (95% CI: 1.38, 4.16, p=0.0020) without CLB (both in favour of treatment) \| \| Outcome of interest—OS \| NR \| \| Outcome of interest—QoL \| NR \| \| Safety \| Severe (grade 3/4) AEs: NR (no meta-analysis was conducted for this outcome) \|   **UK retrospective review with before-and-after data [21]**   \| Reduction in seizure frequency (SF) (primary and secondary outcomes not stated) \| Overall: 6/16 patients (37.5%) had a >30% to <75% reduction in SF.  5 patients (31.0%) had a >75% reduction in SF.  In children with DS, >75% SF reduction including drop seizures was seen in 20% of patients, >30% to <75% reduction in 40% and no improvement in 40%. In children with LGS, >75% reduction in SF including convulsive seizures was seen in 33.33% of patients, >30% to <75% reduction seen in 44.4% and no improvement in 22.2%. \| \| --- \| --- \| \| Other outcome—Safety \| Severe (grade 3/4) AEs: NR \| \| Outcome of interest—OS \| NR \| \| Outcome of interest—QoL \| NR \|   **Korean retrospective review with before-and-after data [22]**   \| Frequency of motor seizures experienced (primary and secondary outcomes not stated) \| DS:  At 3 months, 1/10 patients (10.0%) were seizure-free, 2 (20.0%) had a reduction in seizure frequency >50%, and 60.0% of the enrolled patients experienced no effect.  At 6 months, none of the patients were “seizure-free” and 2 patients (20.0%) showed a reduction in seizure frequency of 50% or more.  LGS:  At 3 months, 8/34 patients (23.5%) were seizure-free, 3 (8.8%) had a reduction in seizure frequency >50%, and 7 (20.6%) showed a reduction in seizure frequency <50% (=52.9% reduction in seizure frequency).  At 6 months, 4 (11.8%) were seizure-free, 3 (8.8%) had a reduction in seizure frequency >50%, and 3 (8.8%) showed a reduction in seizure frequency <50% (=29.4% reduction in seizure frequency). \| \| --- \| --- \| \| Other outcome—Safety \| Severe (grade 3/4) AEs: NR. The authors reported that no life-threatening adverse event was reported in the LGS and DS groups during the observation period. \| \| Outcome of interest—OS \| NR \| \| Outcome of interest—QoL \| NR \| | | | |
| Study designs | \| Trial \| Patients \| Design \| Follow-up \| \| --- \| --- \| --- \| --- \| \| GWPCARE1 (NCT02091206) \| N=120, DS \| Multicentre, phase II/III RCT, double-blind, placebo-controlled \| 14 weeks \| \| GWPCARE2 (NCT02224703) \| N=198, DS \| Multicentre, phase III RCT, double-blind, placebo-controlled \| 14 weeks \| \| GWPCARE3 (NCT02224560) \| N=225, LGS \| Multicentre, phase III RCT, double-blind, placebo-controlled \| 24 weeks (+4 weeks for safety) \| \| GWPCARE4 (NCT02224690) \| N=171, LGS \| Multicentre, phase III RCT, double-blind, placebo-controlled \| 24 weeks (+4 weeks for safety) \| \| GWPCARE5 (NCT02224573) \| N=315, DS  N=171, LGS \| Open-label extension of GWPCARE1/2 (DS) and GWPCARE3/4 (LGS) \| 150 weeks \| \| Expanded Access Program (NCT03676049) \| N=152, DS, LGS \| NA \| 144 weeks \| \| Meta-analysis of GWPCARE1-4 \| N=318, DS  N=396, LGS \| Meta-analysis \| As above \| \| Post hoc analysis of GWPCARE 3-4 \| N=235, LGS  N=161, LGS \| Pooled analysis \| 14 weeks \| \| UK retrospective review with before-and-after data \| N=5, DS  N=9, LGS  N=2, Other \| Single centre, retrospective review with before-and-after data \| Mean time on treatment was 9.4 (range 3 to 20 months) \| \| Korean retrospective review with before-and-after data \| N=10, DS  N=34, LGS \| Single centre, retrospective review with before-and-after data \| 6 months \| | | | |

| Treatment | Chenodeoxycholic acid (CDCA) | | | |
| --- | --- | --- | --- | --- |
| Evidence rating | I | | | |
| Population | ***Indication***  Infants, children, and adolescents aged 1 month to 18 years and adults with inborn errors of primary bile acid synthesis due to sterol 27 hydroxylase deficiency (presenting as cerebrotendinous xanthomatosis) | | ***Incidence/eligible population***  Incidence: <1–3/100,000 total population [23]  Prevalence: 3–5/100,000 white US/European population [23] | |
| Intervention | **Dose**  5–15 mg/kg/day  Minimum dose: 50 mg  Dose adjustment: 50 mg steps | **Frequency**  Daily | **Duration**  Ongoing | **Stopping criteria**  Lack of efficacy, adverse events |
| Comparator | Replacement therapy with bile acids such as chenodeoxycholic acid, ursodeoxycholic acid, cholic acid, or taurocholic acid | | | |
| Outcomes | **Turkish retrospective review with before-and-after data [24]**   \| Primary outcome—Cholestanol level \| Plasma cholestanol concentrations decreased dramatically with therapy \| \| --- \| --- \| \| Key secondary outcomes—Skeletal findings \| *After mean/median CDCA treatment duration 2.3/2.3 years:*  Plasma calcium, phosphate, and alkaline phosphatase concentrations all within normal ranges  No difference in plasma 25-hydroxy-vitamin D vs. before treatment.  No difference in bone mineral density Z scores vs. before treatment. \| \| Outcome of interest—OS \| NR \| \| Outcome of interest—QoL \| NR \| \| Safety \| Severe (grade 3/4) AEs: NR \|   **Dutch retrospective review with before-and-after data [25]**   \| Key outcome—Symptoms \| EDSS scores improved in 50% of patients and in all patients aged <24 over median 8 years.  Modified Rankin Scale improved in 58% of patients and in all patients aged <24 over median 8 years. \| \| --- \| --- \| \| Outcome of interest—OS \| NR \| \| Outcome of interest—QoL \| NR \| \| Safety \| Severe (grade 3/4) AEs: NR \|   **Dutch and Italian retrospective reviews with before-and-after data (pooled) [26]**   \| Key outcome—Cholestanol levels \| *Both countries*  Significant decrease from baseline to any post-treatment visit (p<0.001)  Mean (SD) baseline cholestanol levels 76.5 μmol/L (± 39.0) in the Dutch study and 87.8 μmol/L (± 39.2) in the Italian study.  Cholestanol levels decreased by approximately 70 μmol/L over 10 years. \| \| --- \| --- \| \| Outcome of interest—Clinical symptoms \| *Netherlands*  Psychiatric impairment resolved, improved, or stabilised in most patients with impairment at baseline.  Cognitive symptoms resolved, stabilised, or improved in all patients with impairment at baseline.  Epilepsy resolved and polyneuropathy stabilised or improved in all patients with these symptoms at baseline.  Rankin scale scores improved in 15%, stabilised in 69% and deteriorated in 15%; median 0.0 change from baseline.  Expanded Disability Status Scale scores improved in 23%, stabilised in 54% and deteriorated in 23%; median 0.0 change from baseline.  *Italy*  Neurological impairment stabilised in 45% with impairment at baseline.  Psychiatric impairment improved or stabilised in 92% with impairment at baseline.  Cognitive impairment stabilised in 73% with impairment at baseline.  Diarrhoea stabilised, improved, or disappeared in 93% with this symptom at baseline.  Rankin scale scores did not deteriorate in 62%; median 0.0 change from baseline.  Expanded Disability Status Scale scores improved in 4%, stabilised in 46% and deteriorated in 50%; median 0.5 change from baseline. \| \| Outcome of interest—OS \| NR \| \| Outcome of interest—QoL \| NR \| \| Safety \| Severe (grade 3/4) AEs: <30% (Netherlands 3%, Italy 18%) \|   **French retrospective review with before-and-after data [27]**   \| Primary outcome—Cholestanol level \| Plasma cholestanol levels normalised with treatment  within a few months from baseline 62 ± 25 μmol/l (range: 20–98 μmol/l to mean 9 μmol/L (range 2–16, p<0.001) \| \| --- \| --- \| \| Key outcome—Clinical symptoms \| *Expanded Disability Status Scale (EDSS):*  Over 5 years, EDSS worsened in 3 (25%) patients improved in 3 (25%) patients and remained stable in 6 (50%) patients.  The score changed from 2.5 ± 1.9 to 1.9 ± 1.6 (p=0.034) and remained stable at mean 3.4 (range 0–8) years.  *Scale for the Assessment and Rating of Ataxia (SARA):*  In six patients with data, mean scores improved significantly from 5.3 ± 3.9 (range 1.5–12) to 2.0 ± 1.4 (range 1–4), p=0.043. \| \| Outcome of interest—OS \| NR \| \| Outcome of interest—QoL \| NR \| \| Safety \| Severe (grade 3/4) AEs: NR \|   **US retrospective review with before-and-after data [28]**   \| Primary outcome—Cholestanol level \| Mean plasma cholestanol decreased from 32 mg/L at baseline to 6.0 mg/L with CDCA therapy (normal range <5.0 mg/L); 63% achieved normal cholestanol levels of <5.0 mg/L. \| \| --- \| --- \| \| Key secondary outcome—Clinical symptoms \| Improvement: 57%  Deterioration: 20% (all of whom were ≥25 years with significant neurological disease at diagnosis) \| \| Outcome of interest—OS \| NR \| \| Outcome of interest—QoL \| NR \| \| Safety \| Severe (grade 3/4) AEs: NR \| | | | |
| Study designs | \| Trial \| Patients \| Design \| Follow-up \| \| --- \| --- \| --- \| --- \| \| Turkish retrospective review with before-and-after data [24] \| N=7, mean age at diagnosis 22.3 years \| Single centre, retrospective review with before-and-after data \| Mean treatment duration 2.3 years \| \| Dutch retrospective review with before-and-after data [25] \| N=56 \| Single centre, retrospective review with before-and-after data \| Median 8 years \| \| Dutch and retrospective reviews with before-and-after data [26] \| N=63 (Netherlands n=35, mean age at diagnosis 25.6 years, Italy n=28, mean age at diagnosis 35.0 years) \| Pooled analysis \| Netherlands: median treatment duration 9 years  Italy: median treatment duration 5.75 years \| \| French retrospective review with before-and-after data [27] \| N=14, mean age at evaluation 29 years (range 8–59) \| Single centre, retrospective review with before-and-after data \| Mean treatment duration 5 years (range 2–9) \| \| US retrospective review with before-and-after data [28] \| N=43, mean age at diagnosis 32 years \| Multicentre, retrospective review with before-and-after data \| Mean follow-up 8 years \| | | | |

| Treatment | Cerliponase alfa | | | |
| --- | --- | --- | --- | --- |
| Evidence rating | C+ | | | |
| Population | ***Indication***  Patients of any age with a confirmed diagnosis of CLN2 disease. | | ***Incidence/eligible population***  Incidence 0.02–0.9/100,000 live births [29]  Prevalence <0.1/100,000 population, 0.2/100,000 live births [29] | |
| Intervention | **Dose**  300 mg infused into cerebral ventricle  Patients <2 years: lower doses recommended  Pre-treatment with antihistamines ± antipyretics recommended | **Frequency**  Once every 2 weeks | **Duration**  Ongoing | **Stopping criteria**  Subject to benefit–risk assessment |
| Comparator | No alternative, symptom control only | | | |
| Outcomes | **Study 190-201/202 [29-31]**   \| Primary outcome—Time to 2-point decline in motor and language domains of the CLN2 Clinical Rating Scale \| Median time not reached in treated patients; 345 days (49.3 weeks) in historical controls. \| \| --- \| --- \| \| Key secondary outcomes—Clinical response \| *Combined motor–language score:*  HR for 2-point decline (treated vs. historical control): 0.08 (95% CI 0.02, 0.23), p<0.001.  *Responder rate (no change or improvement in motor–language score):*  87% of treated patients at week 48.  100% vs. 43% at week 96 (difference 57%, p<0.0001). \| \| Outcome of interest—OS \| NR \| \| Outcome of interest—QoL \| *From baseline to end of stable dose period (week 49):*  PedsQL Generic Core Scale, Parent Report for Toddlers: improvement of ~4.3% to week 48.  PedsQL Family Impact Module: improvement of ~6%.  CLN2 Disease-based QoL: improvement of ~10.9%. \| \| Safety \| Severe (grade 3/4) AEs: >30% (58%) \| \| **Study 190-203 [32]** \| \| \| Primary outcome—Rate of decline in motor and language domains of the CLN2 Clinical Rating Scale \| *Combined motor–language (ML) score (at 48 weeks):*  Mean (SD) rate of decline was 0.14 (0.262) points for treated children and 1.24 (1.022) points for controls (mean difference: 1.10; 95% CI 0.69, 1.52)  Pre- vs. post-treatment analysis: The ML score remained stable in 11 patients (treatment duration 11-43 months), improved in 1 patient by 1 point (in the motor domain) after 13 months of treatment, and declined in 2 patients by 1 point (in the motor domain) after 15 and 58 months of treatment \| \| Key secondary outcomes—Clinical response \| NR \| \| Outcome of interest—OS \| NR \| \| Outcome of interest—QoL \| NR \| \| Safety \| Severe (grade 3/4) AEs: NR \| | | | |
| Study designs | \| Trial \| Patients \| Design \| Follow-up \| \| --- \| --- \| --- \| --- \| \| Study 190-201 (NCT01907087) \| N=23 \| Multicentre, single-arm, open-label, phase I/II \| 96 weeks \| \| Study 190-202 (NCT02485899) \| Extension of Study 190-201 \| Up to 240 weeks \| \| Study 190-203  (NCT02678689) \| N=14 (n=12 matched to historical controls) \| Multicentre, single-arm, open-label, phase II \| Interim analysis (48 weeks); pre-post analysis up to 58 months \| | | | |

| Treatment | Dinutuximab beta | | | |
| --- | --- | --- | --- | --- |
| Evidence rating | A (high risk neuroblastoma, maintenance (HRNB)), B+ (relapsed/refractory high-risk neuroblastoma (RRNB)) | | | |
| Population | ***Indication***  High-risk neuroblastoma in patients aged ≥12 months, who have previously received induction chemotherapy and achieved at least a partial response, followed by myeloablative therapy and stem cell transplantation (HRNB)  Patients with history of relapsed or refractory neuroblastoma, with or without residual disease (RRNB) | | ***Incidence/eligible population***  1/100,000 children <15 years [33, 34] | |
| Intervention | **Dose**  Based on BSA; a total of 100 mg/m^2^ per course  Two modes of administration:   - Continuous infusion of 10 mg/m^2^/day over the first 10 days of each course - Daily infusions of 20 mg/m^2^ over 8 hours, on the first 5 days of each course | **Frequency**  5 × 35-day courses | **Duration**  5 × 35-day courses | **Stopping criteria**  Completion of treatment; disease progression, adverse events |
| Comparator | No effective alternative.  Isotretinoin used historically as part of best supportive care. | | | |
| Outcomes | **HRNB**  **HRNBL-1/SIOPEN [35-43]**   \| Primary outcome—EFS (3-year) \| Dinutuximab beta + isotretinoin 56% (95% CI: 49, 63)  Dinutuximab beta + isotretinoin + SC IL-2 60% (95% CI: 53, 66)  p=0.76 between groups  Dinutuximab beta + isotretinoin ± SC IL-2 vs. isotretinoin (historical control):  EFS HR=1.74 (95% CI: 1.34,2.24), p<0.0001 or expressed as 1/HR:  EFS HR=0.57 (95% CI: 0.45,0.75), p<0.0001  Dinutuximab beta (long-term infusion) + isotretinoin 64% (± 0.04)  Dinutuximab beta (long-term infusion) + isotretinoin +SC IL-2 56% (± 0.05) \| \| --- \| --- \| \| Key secondary outcome—EFS (5-year) \| Dinutuximab beta + isotretinoin vs. dinutuximab beta + isotretinoin + SC IL-2: 53% vs. 57%  Dinutuximab beta + isotretinoin ± SC IL-2 vs. isotretinoin alone  57% vs. 42% (p<0.001)  Dinutuximab beta + isotretinoin ± SC IL-2 vs. isotretinoin historical control, adjusted for prior consolidation treatment, age at diagnosis, MYCN status and INSS stage:  EFS HR=1.75 (95% CI: 1.36, 2.25), p<0.0001 or expressed as 1/HR:  EFS HR=0.57 (95% CI: 0.44, 0.74), p<0.0001  Dinutuximab beta + isotretinoin ± SC IL-2 vs. isotretinoin historical control:  EFS HR=1.5 (95% CI: 1.3, 1.7), p<0.0001 or expressed as 1/HR:  EFS HR=0.67 (95% CI: 0.59, 0.77), p<0.0001 \| \| Outcome of interest—OS (secondary) \| *3-year OS:*  Dinutuximab beta 69% (95% CI: 62, 75)  Dinutuximab beta + SC IL-2 70% (95% CI: 63, 75)  Dinutuximab beta (long-term infusion) + isotretinoin 71% (± 0.04)  Dinutuximab beta (long-term infusion) + isotretinoin +SC IL-2 68% (± 0.05).  *5-year OS:*  Dinutuximab beta + isotretinoin vs. dinutuximab beta + isotretinoin + SC IL-2: 63% vs. 62%  Dinutuximab beta + isotretinoin ± SC IL-2 vs. isotretinoin alone  64% vs. 50% (p<0.001)  Dinutuximab beta + isotretinoin ± SC IL-2 vs. isotretinoin historical control, adjusted for prior consolidation treatment, age at diagnosis, MYCN status and INSS stage:  OS HR=0.70 (95% CI: 0.54, 0.91)  Dinutuximab beta + isotretinoin ± SC IL-2 vs. isotretinoin historical control:  OS HR=1.6 (95% CI: 1.4, 1.9), p=0.0070 or expressed as 1/HR:  OS HR=0.63 (95% CI: 0.53, 0.71), p=0.0070 \| \| Outcome of interest—QoL \| NR \| \| Safety \| Severe (grade 3/4) AEs: >30% \|   **RRNB**  **APN311-202** [33, 44-46]   \| Primary outcome—Use of IV morphine \| Patients receiving long-term infusions required significantly less IV morphine than patients who received bolus infusions \| \| --- \| --- \| \| Co-primary outcome—Antibody effects \| Long-term infusion of dinutuximab beta + SC IL-2 resulted in strong  activation of antibody effector functions \| \| Key secondary outcome—EFS \| *2-year EFS:*  Dinutuximab beta: 59%  Dinutuximab beta + SC IL-2: 65% \| \| Outcome of interest—OS (secondary) \| *2-year OS:*  Dinutuximab beta: 79%  Dinutuximab beta + SC-IL-2: 84%  p=0.904 \| \| Outcome of interest—QoL \| NR \| \| Safety \| Severe (grade 3/4) AEs: >30% (95%) \|   **APN311-303 202 [33, 47]**   \| Primary outcome—Use of IV morphine \| Decreased from 96% in cycle 1 to 11% in cycle 5 \| \| --- \| --- \| \| Key secondary outcome—EFS (2-year) \| 35% \| \| Outcome of interest—OS (secondary; 2-year) \| 75%  In relapsed patients at 2 years: 69% (dinutuximab beta) vs. 30% (non-immunotherapy Garaventa control), p=0.002 \| \| Outcome of interest—QoL \| NR \| \| Safety \| Severe (grade 3/4) AEs: >30% (94%) \|   **Pooled APN311-202 + APN311-303 data 202 [33, 43]**   \| Outcome of interest—OS \| *3-year OS:*  Dinutuximab beta: 50%  Historical controls from Italian registry (retrospective): 24%  p=0.0031  Historical controls from HRNBL1 randomisation 1: 28%  p=0.0302  Dinutuximab beta + SC IL-2 vs. different historical controls:  OS HR=0.43 (95% CI: 0.24, 0.78), p=0.00542 (vs. Italian registry)  OS HR=0.56 (95% CI: 0.32, 0.97), p=0.0376 (vs. randomisation 1) \| \| --- \| --- \| \| Outcome of interest—QoL \| NR \| \| Safety \| Severe (grade 3/4) AEs: NR \| | | | |
| Study designs | \| Trial \| Patients \| Design \| Follow-up \| \| --- \| --- \| --- \| --- \| \| HRNBL-1/SIOPEN  APN311-302 R2 (NCT01704716) \| N=406, HRNB \| Multicentre, phase III RCT, open label  (randomisation R2) \| 5.6 years \| \| HRNBL-1/SIOPEN  APN311-302 R4 (NCT01704716) \| N=408, HRNB \| Multicentre, phase III RCT, open label  (randomisation R4) \| Up to 2 years \| \| APN311-202 (NCT01701479) \| N=44, RRNB \| Multicentre, phase II, single-arm, prospective \| Up to 3 years \| \| APN311-303 \| N=54, RRNB  N=30 historical controls \| Single centre, single-arm, open-label, retrospective \| Up to 3 years \| | | | |

| Treatment | Glibenclamide oral suspension | | | |
| --- | --- | --- | --- | --- |
| Evidence rating | C+ | | | |
| Population | ***Indication***  Newborns, infants, and children with neonatal diabetes mellitus | | ***Incidence/eligible population***  Incidence: ~1/100,000 live births [48]  Prevalence: 0.2–1/100,000 population [48, 49] | |
| Intervention | **Dose**  Oral suspension: initial dose 0.2 mg/kg/day increased within 6 days to final total dose 1mg/kg; maximum dose 1ml/kg/day | **Frequency**  Daily | **Duration**  Transient disease: treatment discontinuation at few weeks to 5 years of age  Permanent disease: lifetime treatment | **Stopping criteria**  Uncontrolled HbA1c (need for insulin) |
| Comparator | Glibenclamide tablets; other sulphonylurea tablets (indicated in adults); insulin | | | |
| Outcomes | **Neonatal Diabetes International Collaborative Group study (includes different glibenclamide formulations) [50]**   \| Primary outcome—Withdrawal of insulin therapy \| 90% able to stop insulin therapy  Four patients were insulin independent for >15 months \| \| --- \| --- \| \| Key secondary outcome—HbA1c \| Mean 1.7% reduction by week 12 (p<0.001)  Sustained reduction in 12 patients who were insulin-independent for >1 year \| \| Outcome of interest—OS \| NR \| \| Outcome of interest—QoL \| NR \| \| Safety \| Severe (grade 3/4) AEs:<30% (0%) \|   **GLIDKIR Study [51]**   \| Primary outcome—Withdrawal of insulin therapy \| 12 months: 1/18 patients discontinued insulin therapy  18 months: 18/18 patients discontinued insulin therapy \| \| --- \| --- \| \| Key secondary outcome—HbA1c \| Reduction in HbA1c over 18 months:  1.55% (95% CI: -3.8, 0.1%), p<0.0001 \| \| Outcome of interest—OS \| NR \| \| Outcome of interest—QoL \| NR \| \| Safety \| Severe (grade 3/4) AEs:<30% (0%) \|   **NEOGLI Study[52]**   \| Primary outcome—Hedonic visual scale (acceptability of treatment) \| *Satisfied with suspension at 4 months:*  Children <5 years: 67%  Children ≥5 years: 25%  Parents of children <5 years: 67%  Parents of children ≥5 years: 75% \| \| --- \| --- \| \| Key secondary outcome—HbA1c \| Median HbA1c  Glibenclamide suspension: 6.1%  Glibenclamide tablets: 6.4%  p=0.07 \| \| Key secondary outcome—Safety \| Median frequencies of hypoglycaemia and hyperglycaemia:  <5% of routine blood glucose assays  Similar with glibenclamide suspension vs. glibenclamide tablets  Severe (grade 3/4) AEs: NR \| \| Outcome of interest—OS \| NR \| \| Outcome of interest—QoL \| NR \|   **10-year multicentre follow-up cohort study [53]**   \| Primary outcome—Failure of sulfonylurea treatment \| No need for insulin therapy: 75/81 patients (93%) \| \| --- \| --- \| \| Key secondary outcome—HbA1c \| Median HbA1c decreased from 8.1% before sulfonylurea to  5.9% at 1 year (p<0.0001 vs. pre-treatment)  6.4% at last follow-up (median 10.3 years; p<0.0001 vs. 1 year) \| \| Outcome of interest—OS \| NR \| \| Outcome of interest—QoL \| NR \| \| Safety \| Severe (grade 3/4) AEs: <30% \| | | | |
| Study designs | \| Trial \| Patients \| Design \| Follow-up \| \| --- \| --- \| --- \| --- \| \| Neonatal Diabetes International Collaborative Group study (NCT00334711) \| N=49 \| Multicentre, single-arm, open-label, prospective \| Up to 12 months \| \| GLIDKIR Study (NCT00610038) \| N=18 \| Single centre, single-arm, open-label, phase II, prospective \| Up to 18 months \| \| NEOGLI Study (NCT02375828) \| N=10 \| Single centre, single-arm, open-label, phase III, prospective \| 4 months \| \| 10-year multicentre follow-up study (NCT02624817) \| N=81 \| Multicentre, single-arm, phase IV, prospective \| 10.2 years (median) \| | | | |
| Treatment | Metreleptin | | | |
| Evidence rating | C+ (generalised lipodystrophy (LD)) and I (acquired partial lipodystrophy (LD)) | | | |
| Population | ***Indication***  Replacement therapy adjunctive to diet to treat the complications of LD:   - Confirmed congenital generalised LD (Berardinelli-Seip syndrome) or acquired generalised LD (Lawrence syndrome) in children ≥2 years - Familial or acquired partial LD (Barraquer-Simons syndrome) confirmed in patients >12 years in whom standard treatments failed to achieve adequate metabolic control | | ***Incidence/eligible population***  Prevalence: <0.1–1.5/100,000 population (generalised LD), <0.3/100,000 population (partial LD eligible for metreleptin) [54, 55] | |
| Intervention | **Dose**  Males & females ≤ 40 kg:  Starting dose: 0.06 mg/kg/day  Dose adjustments: 0.02 mg/kg  Maximum daily dose: 0.13 mg/kg/day  Males >40 kg:  Starting dose: 2.5 mg/day  Dose adjustments: 1.25–2.5 mg  Maximum dose: 10 mg/day  Females >40 kg:  Starting dose: 5 mg/day  Dose adjustments: 1.25–2.5 mg  Maximum dose: 10 mg/day | **Frequency**  Daily | **Duration**  Ongoing | **Stopping criteria**  Lack of efficacy, tolerability, end of life |
| Comparator | There are currently no treatments allowing reconstitution of adipose tissue and correcting lipoatrophy, a major symptom of lipodystrophy | | | |
| Outcomes | **Generalised LD**  **Study 991265/20010769 [54, 56]**   \| Co-primary outcome—HbA1c \| Mean change from baseline to month 12: –2.2%, (95% CI: –2.7, –1.6), p<0.001 \| \| --- \| --- \| \| Co-primary outcome—Fasting TGs \| Mean change from baseline to month 12: –32.1% (95% CI: –51.0, –13.2), p=0.001 \| \| Key secondary outcome—Responder rates at month 12 \| ≥1% decrease in HbA1c or ≥30% decrease in fasting serum TG: 80%  ≥2% decrease in HbA1c or ≥40% decrease in fasting serum TG: 66% \| \| Outcome of interest—OS \| NR \| \| Outcome of interest—QoL \| NR \| \| Safety \| Severe (grade 3/4) AEs <30% (29%) \|   **Study FHA101 [54]**   \| Co-primary outcome—HbA1c \| Mean change from baseline to month 12: –1.2% (95% CI: 4.3, 2.0), p=0.360 \| \| --- \| --- \| \| Co-primary outcome—Fasting TGs \| Mean change from baseline to month 12: –26.9% (95% CI: –124.1, 70.4), p=0.486 \| \| Key secondary outcome—Responder rates at month 12 \| ≥1% decrease in HbA1c or ≥30% decrease in fasting serum TG: 50%  ≥2% decrease in HbA1c or ≥40% decrease in fasting serum TG: 50% \| \| Outcome of interest—OS \| NR \| \| Outcome of interest—QoL \| NR \| \| Safety \| Severe (grade 3/4) AEs: NR \|   **Cook et al 2019 [57]**   \| Key outcomes (primary/secondary not identified) \| *At month 12:*  Mean % TG decreased by 53% (p<0.001) from baseline  Mean HbA1c decreased by 1.9 percentage points (p<0.001) from baseline  TG ≤200 mg/dL achieved by 50% (vs. 5% at baseline)  HbA1c ≤7% achieved by 57% (vs. 22% at baseline) \| \| --- \| --- \| \| Outcome of interest—OS \| NR \| \| Outcome of interest—QoL \| NR \| \| Safety \| Severe (grade 3/4) AEs: NR \|   **Partial LD**  **Study 991265/20010769 [54]**   \| Co-primary outcome—HbA1c \| Mean change from baseline to month 12: –0.6% (95% CI: –1.0, –0.2), p=0.005 \| \| --- \| --- \| \| Co-primary outcome—Fasting TGs \| Mean change from baseline to month 12: –20.8% (95% CI: –37, to –4.6), p=0.013 \| \| Key secondary outcome—Responder rates at month 12 \| ≥1% decrease in HbA1c or ≥30% decrease in fasting serum TG: 68%  ≥2% decrease in HbA1c or ≥40% decrease in fasting serum TG: 43% \| \| Outcome of interest—OS \| NR \| \| Outcome of interest—QoL \| NR \| \| Safety \| Severe (grade 3/4) AEs: NR \|   **Study FHA101 [54]**   \| Co-primary outcome—HbA1c \| Mean change from baseline to month 12: –0.4% (95% CI: –1.0, 0.2), p=0.210 \| \| --- \| --- \| \| Co-primary outcome—Fasting TGs \| Mean change from baseline to month 12: –8.7% (95% CI: –29.1, 46.4), p=0.640 \| \| Key secondary outcome—Responder rates (Month 12) \| ≥1% decrease in HbA1c or ≥30% decrease in fasting serum TG: 29%  ≥2% decrease in HbA1c or ≥40% decrease in fasting serum TG: 14% \| \| Outcome of interest—OS \| NR \| \| Outcome of interest—QoL \| NR \| \| Safety \| Severe (grade 3/4) AEs: NR \|   **Cook et al 2019 [57]**   \| Key outcomes (primary/secondary not identified) \| *At month 12:*  Mean % TG decreased by 21.5% (p<0.005) from baseline  Mean HbA1c decreased by 0.5 percentage points (p<0.1) from baseline  TG ≤200 mg/dL achieved by 52% (vs. 29% at baseline)  HbA1c ≤7% unchanged vs. baseline (32%) \| \| --- \| --- \| \| Outcome of interest—OS \| NR \| \| Outcome of interest—QoL \| NR \| \| Safety \| Severe (grade 3/4) AEs: NR \| | | | |
| Study designs | \| Trial \| Patients \| Design \| Follow-up \| \| --- \| --- \| --- \| --- \| \| 991265 (pilot)/ 20010769 (long-term extension) \| N=107, generalised LD n=66 (mean age 18 years), partial LD n=41 (mean age 34 years) \| Single-centre, single-arm, open-label, prospective \| Generalised LD: efficacy reported at months 24 (n=25), 36 (n=17), and 48 (n=11)  Partial LD: efficacy reported at months 24 (n=8) and 36 (n=7) \| \| FHA101 (NCT00677313) \| N=41, generalised LD n=9, partial LD n=32 \| Multicentre, single-arm, open-label, prospective, US early access program \| 12 months \| \| Cook et al. 2019 \| N=53, generalised LD n=28, partial LD n=25 \| Multicentre, single-arm, open-label, retrospective, European early-access \| Metreleptin exposure: mean 5.4 years; maximum: 14.7 years \| | | | |

| Treatment | Nusinersen | | | |
| --- | --- | --- | --- | --- |
| Evidence rating | A (type I Spinal Muscular Atrophy (SMA)), B+ (type II/III SMA), C+ (presymptomatic SMA) | | | |
| Population | ***Indication***  Infantile-onset (type I) SMA  Later-onset (type II/III) SMA  Presymptomatic SMA | | ***Incidence/eligible population***  Incidence 8.5–10/100,000 live births (all types; 58% are type I, 29% type II, 13% type III) [58] [59] | |
| Intervention | **Dose**  12 mg (5 mL) per administration | **Frequency**  4 loading doses on days 0, 14, 28, and 63, followed by maintenance dose every 4 months  Administered as an intrathecal bolus injection over 1–3 minutes, using a spinal anaesthesia needle by healthcare professionals experienced in performing lumbar punctures  Sedation may be required  Ultrasound-guided administration may be required | **Duration**  Ongoing | **Stopping criteria**  No demonstrated maintenance or improvement of motor milestone function (assessed using the HINE section 2); need for permanent invasive ventilation |
| Comparator | No clinically effective (disease-modifying) alternative treatments | | | |
| Outcomes | **Infantile-onset (type I) SMA**  **ENDEAR [58, 60]**   \| Co-primary outcome—HINE motor milestone response \| Nusinersen 51% vs. sham control 0%, difference 50.58% (95% CI: 31.81, 66.48), p<0.0001 over 187-280 days \| \| --- \| --- \| \| Co-primary outcome—EFS (time to death or permanent ventilation) \| 61% vs. 32%; HR 0.53 (95% CI: 0.32, 0.89), p=0.005 \| \| Outcome of interest—OS (secondary) \| 84% vs. 61%, HR 0.37 (95% CI: 0.18, 0.77), p=0.004 \| \| Outcome of interest—QoL \| NR \| \| Safety \| Severe (grade 3/4) AEs: >30% (nusinersen 56% vs. control 80%) \|   **CS3A [58]**   \| Primary outcome—HINE motor milestone response \| 60% had improvement \| \| --- \| --- \| \| Key secondary outcome—EFS (time to death or permanent ventilation) \| 55% at last visit \| \| Outcome of interest—OS (secondary) \| At 3 years, 25% had died \| \| Outcome of interest—QoL \| NR \| \| Safety \| Severe (grade 3/4) AEs: >30% (100%) \|   **SHINE (long-term extension ) [61-63]**   \| Primary outcome—safety \| Long-term safety profile consistent with previously reported findings \| \| --- \| --- \| \| Key secondary outcome—HFSME motor function \| *Mean score at day 240:*  Nusinersen (ENDEAR)–nusinersen (SHINE): 7.3 (6.82, n=50)  Sham (ENDEAR)–nusinersen (SHINE): 0 (n=17) \| \| Other outcome—Children's Hospital of Philadelphia Infant Test of Neuromuscular Disorders (CHOP INTEND) \| Nusinersen (ENDEAR) ENDEAR baseline: mean 27.84 (SD 8.09; n=60)  Nusinersen (ENDEAR) SHINE MMDR Day 1: 44.6 (11.33; n = 59)  Nusinersen (ENDEAR)–nusinersen (SHINE): 45.8 (SD 13.27; n=56) at day 480  Sham (ENDEAR) ENDEAR baseline: 29.59 (SD 7.97; n=22)  Sham (ENDEAR) sham MMDR 1: 22.5 (10.07; n = 22)  Sham (ENDEAR)–nusinersen (SHINE): 24.5 (SD 12.33; n=20) at day 480 \| \| Other outcomes—WHO motor milestones \| Nusinersen (ENDEAR)–nusinersen (SHINE): sitting unassisted: 37/58 (64%), standing with assistance: 11/58 (19%), walking with assistance: and 4/58 (7%) at day 480.  Sham (ENDEAR)–nusinersen (SHINE):1/20 (5%) was sitting without support at day 480 \| \| Outcome of interest—OS \| NR \| \| Outcome of interest—QoL \| NR \|   **Canadian registry study [64]**   \| CHOP-INTEND \| Increase of 14.8 per year (median follow-up 12 months) \| \| --- \| --- \|   **Japanese before-after study [65]**   \| CHOP-INTEND \| No improvement \| \| --- \| --- \|   **Brazilian before-after study (type I) [66]**   \| CHOP-INTEND \| Mean change of 4.9, 5.9, 6.6, and 14 points after 6, 12, 18, and 24 months, respectively \| \| --- \| --- \| \| HINE-2 \| 28.6% of patients acquired some motor milestone or gained at least three points on the HINE-2 \|   **Russian before-after study [67]**   \| CHOP-INTEND \| Mean change was 9.8 points (N=30, *p* <0.001) after 6 months \| \| --- \| --- \| \| HINE-2 \| Mean change was 3.7 ± 1.9 points (N=39, *p*<0.001) after 6 months \|   **Hungarian before-after study [68]**   \| CHOP-INTEND \| 14.9 (± 5.1) improvement (p=0.016) at day 307 (N=7). All patients have improved by more than four points. \| \| --- \| --- \|   **Italian cohort with before-and-after data [69]**   \| Primary outcome 1—Children's Hospital of Philadelphia Infant Test of Neuromuscular Disorders (CHOP INTEND) \| Baseline:18.09 (SD 14.22)  12 months: 24.81 (SD 18.85) (mean change from baseline: 6.72 [SD 8.33])  24 months: 26.75 (SD 19.45).(mean change from baseline: 8.66 [9.35]) \| \| --- \| --- \| \| Primary outcome 2—HINE-2 motor milestones \| Baseline:0.88 (SD 1.33)  12 months: 2.75 (SD 3.87) (mean change from baseline: 1.87 [SD 3.18])  24 months: 3.50 (SD 4.96).(mean change from baseline: 2.62 [4.39]) \| \| Other outcomes—sitting position \| By 24‐months, 21/68 reached the sitting position. Of these 21, 10 were also able to half roll to both sides independently, 5 to roll from prone to supine and/or vice versa, 1 was able to maintain “prone on elbows” position independently, 1 was able to maintain four‐point kneeling independently, 1 was able to stand with support, 1 was able to walk with support, and 1 was able to walk independently. \| \| Outcome of interest—OS \| Not reported for the 68 patients analysed in this paper \| \| Outcome of interest—QoL \| NR \| \| Safety \| Severe (grade 3/4) AEs: NR \|   **UK cohort with before-and-after data [70]**   \| The Great Ormond Street (GSR) score (primary and secondary outcomes not stated) \| Baseline: median 5.5 (IQR3-16.8)  Change from baseline at 2 months: median +2.5 (IQR 0.3- 7.3), p<0.001  Change from baseline at 22 months: median 0 (0-4), p=0.04 \| \| --- \| --- \| \| CHOP \| Baseline: median 28.5 (IQR 19.0-34.8)  Change from baseline at 2 months: median +9.5 (IQR 3.5-15.5), p<0.001  Change from baseline at 22 months: median +7 (5-9.5), p<0.001 \| \| Deterioration in respiratory health \| 3/20 cases after 6 months \| \| Outcome of interest—OS \| NR \| \| Outcome of interest—QoL \| NR \| \| Safety \| Severe (grade 3/4) AEs: NR \|   **Israeli before-and-after study [71]**   \| Primary outcome—Respiratory characteristics \| Baseline: 12 patients were using permanent assisted ventilation; 4 were using partial assisted ventilation  2 years: All patients with assisted ventilation remained ventilated; 4 patients who were not previously ventilated required assisted ventilation (partial non-invasive)  *Mean number of hospitalisations:*  Baseline: 0.625 (SD 0.7) per year (in 2 years before treatment)  After treatment: 0.55 (SD 0.8) \| \| --- \| --- \| \| Outcome of interest—OS \| NA Two  patients died from acute respiratory failure during the 1st year of  treatment  2 patients died from acute respiratory failure during the first year of treatment \| \| Outcome of interest—QoL \| NR \| \| Safety \| 1 patient sustained an anoxic brain injuring due to massive aspiration during the second year of treatment (treatment was stopped) \|   **Later-onset (type II/III) SMA**  **CHERISH [72] [73]**   \| Primary outcome—HFMSE motor function (change at 15 months) \| *Final analysis*  LSM change from baseline: nusinersen 3.9, sham –1.0  LSM difference 4.9 (95% CI: 3.1, 6.7) \| \| --- \| --- \| \| Key secondary outcome—clinically meaningful (≥3-point) increase in HFMSE from baseline to month 15 \| 57% vs. 26%, p<0.001 \| \| Revised Upper Limb Module (RULM) (change at 15 months) \| LSM increase from baseline: nusinersen 4.2, sham 0.5 points  LSM difference 3.7 (95% CI: 2.3, 5.0) \| \| Outcome of interest—OS \| NR \| \| Outcome of interest—QoL \| *Assessment of Caregiver Experience with Neuromuscular Disease (ACEND):*  Nusinersen: The impact of caregivers was *reduced* at months 6 and 15 months for 3/7 domains; feeding/grooming/dressing, transfer, and mobility  Sham: The impact on caregivers was *increased* in these domains for the sham group at both assessments  The greatest reduction for caregivers was in the mobility domain: LS mean difference (95%) from baseline to Month 15 of nusinersen vs. sham was 11.9 (3.9, 19.8). (Further data not reported in the abstract)  *Pediatric Quality of Life Inventory (PedsQL):*  LS mean difference for nusinersen vs. sham from baseline to month 15 was 5.0 (95% CI: 0.7, 9.3) for the parent assessment. \| \| Safety \| Severe (grade 3/4) AEs: NR (SAEs: nusinersen 17%, sham 29%) \|   **SHINE (long-term extension) [62, 74] [75, 76]**   \| Primary outcome—safety \| Long-term safety profile consistent with previously reported findings \| \| --- \| --- \| \| Key secondary outcome—HFSME motor function \| *Mean score at day 1:*  Nusinersen (CHERISH)–nusinersen (SHINE): 26.5 (10.85, n=81)  Sham (CHERISH)–nusinersen (SHINE): 21.5 (7.79, n=42)  *Mean score at day 240:*  Nusinersen (CHERISH)–nusinersen (SHINE): 26.0 (SD 11.01. n=61)  Sham (CHERISH)–nusinersen (SHINE): 21.2 (SD 7.75, n=26)  *Mean score at day 480:*  Nusinersen (CHERISH)–nusinersen (SHINE): 26.1 (SD 11.94, n=79)  Sham (CHERISH)–nusinersen (SHINE): 21.2 (SD 8.75, n=40) \| \| Other outcomes—Revised Upper Limb Module (RULM) score \| *Mean score at day 1:*  Nusinersen (CHERISH)–nusinersen (SHINE): 24.1 (SD 5.64; n=78)  Sham (CHERISH)–nusinersen (SHINE): 21.0 (4.28; n=41)  *Mean score at day 480:*  Nusinersen (CHERISH)–nusinersen (SHINE): 25.4 (SD 6.07; n=79)  Sham (CHERISH)–nusinersen (SHINE): 22.7 (SD 4.65; n=41) \| \| Outcome of interest—OS \| NA \| \| Outcome of interest—QoL \| *ACEND scores among caregivers* (mean change from baseline of CHERISH to Day 1170):  Nusinersen (CHERISH)–nusinersen (SHINE) (n=67) subdomains: Feeding/Grooming/Dressing: 10.3 (95% CI: 5.99, 14.54), Sitting/Playing: 1.1 (95% CI: ‐2.18, 4.45), Transfers: 3.5 (95% CI: ‐0.68, 7.70), Mobility: 5.0 (95% CI: ‐0.47, 10.45), and Finance: 0.7 (95% CI: ‐3.76, 5.25).  *PedsQL generic total score (mean change* from baseline of CHERISH to Day 1170):  Nusinersen (CHERISH)–nusinersen (SHINE) (n=63): 2.3 (95% CI: ‐1.08, 5.72)  PedsQL NM score: ‐0.2 (95% CI: ‐3.79,3.49) \|   **Canadian registry study [64]**   \| HFMSE \| Type II:  Increase of 3.25 points/year (4.8 months follow-up)  Type III:  Increase of 5.4 points/year (7.5 months follow-up) \| \| --- \| --- \| \| RULM \| Type II only: Increase of 2 points/year (4.8 months follow-up) \|   **Italian before-and-after study [77]**   \| HFMSE \| Type II only:  Increase of 1.90, SD: ± 3.85 (*p*< 0.001; 95%CI −2.608; −0.892) (12 months follow-up) \| \| --- \| --- \| \| RULM \| Type II only:  Increase of 1.59, SD: ±3 .60 (*p*< 0.001; 95%CI −1.721; −0.198) (12 months follow-up) \|   **Japanese before-and-after study [65]**   \| HFMSE \| Improvement in 80% of patients \| \| --- \| --- \|   **Brazilian before-and-after study (type II/III) [78]**   \| HFMSE \| In the nusinersen group (N=30) mean change was 1.47 points (SD=0.4) at 12 months and 1.60 points (SD = 0.6) after 24 months of treatment.  In the control group (N= 37) mean change was −1.71 points (SD = 0.02) at 12 months and −3.93 points (SD = 0.55) after 24 months of follow-up. \| \| --- \| --- \| \| CHOP-INTEND \| In the nusinersen group mean change was 2.37 points (range: −5 to 10) at 12 months (N=11) and +3.42 (range: 0 to 14) at 24 months (N=7). \|   **German before-after study [79]**   \| FVC \| No significant difference in FVC at baseline and day 300 (p=1.000) and baseline and day 180 (p=1.000) \| \| --- \| --- \|   **Slovenian before-and-after study [80]**   \| Primary outcome—Respiratory support \| Baseline: 6/15 patients required non-invasive (NIV) respiratory support at night for a mean of 458 (SD 168) minutes/day  12 months: 619 (SD 239) minutes/day, p=0.01. No new patients required overnight NIV, \| \| --- \| --- \| \| Outcome of interest—OS \| NA Two  patients died from acute respiratory failure during the 1st year of  treatment  NR \| \| Outcome of interest—QoL \| NR \| \| Safety \| Severe (grade 3/4) AEs: NR \|   **Hungarian before-after study [68]**   \| HFMSE \| 7.2 (range -2 to 17) point increase from baseline (p < 0.001) at day 307 (N=16, type 2 only) \| \| --- \| --- \| \| RULM \| 4.3 (range: 2 to 9) point increase (p=0.031) at day 307 (N=16, type 2 only) \| \| 6MWT \| Increase by 33.9 m was found at day 307 (± 44; range -16.3 to 106.5 m, p=0.007) (N=15, type 3 only) \|   **French before-after study [81]**   \| FVC % predicted \| 66 (SD ± 28) in the nusinersen group (N=12) versus 45 ± 15 in the historical control (N=14), p=0.029; type II only, no baseline measurements \| \| --- \| --- \| \| Sniff Pes (cmH_2_O) \| 50 (SD ± 16) in the nusinersen group (N=12) versus 37 ± 13 in the historical control (N=14), p=0.018; type II only, no baseline measurements \| \| Sniff Pes (%pred) \| 49 (SD ± 17) in the nusinersen group (N=12) versus 37 ± 14 in the historical control (N=14), p=0.028; type II only, no baseline measurements \|   **Infantile onset and later onset (type I and type II/III)**  **EMBRACE [82] [83]**   \| Primary outcome—safety \| Severe (grade 3/4) AEs: >30% (nusinersen: 57% vs. sham 43%) \| \| --- \| --- \| \| Key secondary outcome—HINE-2 motor milestone response \| Nusinersen 79%; sham 29% \| \| Outcome of interest—OS \| NR \| \| Outcome of interest—QoL \| NR \|   **UK before-and-after study [84, 85]**   \| Children's Hospital of Philadelphia Infant Test of Neuromuscular Disorders (CHOP INTEND) (primary and secondary outcomes not stated) \| Baseline: median 31  Post-nusinersen: median 41.5 \| \| --- \| --- \| \| Hospital admissions for respiratory infections \| Baseline: 26 (before treatment)  Post-nusinersen: 8 \| \| Outcome of interest—OS \| NR \| \| Outcome of interest—QoL \| NR \| \| Safety \| Severe (grade 3/4) AEs: NR \|   **Australian before-and-after study [86, 87]**   \| Primary outcome—Forced Vital Capacity (FVC) \| *Annual rate decline z-score:*  Type II SMA: from -0.5 per annum (95% CI 0.26-0.72) to +0.002 (95% CI -0.08-0.006), p=0.009  Type 3 SMA: -0.4 per annum (95% CI 0.1-0.6) to -0.001 (95% CI -0.005-0.002), p=0.3 \| \| --- \| --- \| \| Other outcomes— Hammersmith Functional Motor Scale Expanded (HFMSE) \| Motor muscle response was attained in 5/11 (45%) type II and 6/9 (67%) type III patients. \| \| Outcome of interest—OS \| NR \| \| Outcome of interest—QoL \| NR \| \| Safety \| Severe (grade 3/4) AEs: NR \|   **Italian before-and-after study [88]**   \| Primary outcomes—CHOP-INTEND and Hammersmith Functional Motor Scale Expanded (HFMSE) \| There was an average increase of 4 points for CHOP-INTEND and 3.75 points for HFMSE in SMA II patients after 6 months of treatment (data at baseline not reported) (the authors stated an average trend for CHOP-INTEND scores could not be made for SMA I patients) \| \| --- \| --- \| \| Outcome of interest—OS \| NA Two  patients died from acute respiratory failure during the 1st year of  treatment  NR \| \| Outcome of interest—QoL \| NR \| \| Safety \| Severe (grade 3/4) AEs: NR \|   **Presymptomatic SMA**  **NURTURE [89, 90]**   \| Primary outcome—time to death or respiratory intervention \| Not estimated, because of insufficient events: at last visit (median time on study 34 months), 25/25 children were alive and 0/25 required permanent ventilation \| \| --- \| --- \| \| Achievement of WHO motor milestone \| 100% of infants achieved sitting without support, 92% with two SMN2 copies achieved walking with assistance (100% with three SMN2 copies), 88% walking independently (100% with three SMN2 copies). \| \| Key secondary outcome—HINE-2 motor milestones \| Total scores increased over time for all participants.  Mean (range) total scores increased from a baseline of 2.7 (0–5) to 23.9 (16–26) at the last observed visit, up to and including Day 778, for patients with two SMN2 copies and from 3.2 (0–7) to 26.0 (26–26) for those with three SMN2 copies. \| \| Change from baseline in the CHOP INTEND motor function scale \| At the last visit, mean (range) total score changed to 62.1 (48–64) from 47.0 (SD=10.04) at baseline for those with two SMN2 copies and to 63.4 (58–64) from 51.9 (6.10) in those with three SMN2 copies. \| \| Key secondary outcome—clinically manifest SMA \| At last visit, children were median 34.8 months old and past the expected age of symptom onset for SMA types I or II \| \| Outcome of interest—OS \| 25/25 alive at last visit \| \| Outcome of interest—QoL \| NR \| \| Safety \| Severe (grade 3/4) AEs: <30% (20%) \| | | | |
| Study designs | \| Trial \| Patients \| Design \| Follow-up \| \| --- \| --- \| --- \| --- \| \| ENDEAR (NCT02193074) \| N=122 (121 treated and 80 with nusinersen), type I SMA \| Multicentre, phase III RCT, double-blind, sham-controlled \| 13 months (the study was terminated early after a prespecified interim analysis. The median duration of observation at the final data cut-off was 280 days in the nusinersen arm and 187 days in the sham intervention arm.) \| \| CS3A (NCT01839656) \| N=20, type I SMA \| Multicentre, phase II uncontrolled trial \| 3.7 years \| \| CHERISH (NCT02292537) \| N=126, type II/III SMA \| Multicentre, phase III RCT, double-blind, sham-controlled \| 15 months \| \| EMBRACE (NCT02462759) \| N=21, type II/III SMA \| Multicentre, phase II RCT, double-blind, sham-controlled \| ~3 years \| \| NURTURE (NCT02386553) \| N=25, presymptomatic SMA \| Multicentre, phase II, single-arm, open-label trial \| 2.9 years \| \| SHINE (NCT02594124) \| N=292, type I, II or III SMA \| Open-label extension of all trials except NURTURE \| 6.5 years \| \| Italian cohort with before-and-after data \| N=68, type I SMA \| Multicentre cohort with before-and-after data \| 2 years \| \| Italian cohort with before-and-after data \| N=77, type II SMA \| Multicentre cohort with before-and-after data \| 12 months \| \| UK before-and-after study \| N=12, type I, Ib or II SMA (also reported as N=22 in type I-II SMA patients in a second abstract) (but same outcomes) \| Single-centre before-and-after study \| Median 23 months \| \| UK cohort with before-and-after data (not CS3A) \| N=20, type I SMA \| Cohort with before-and-after data \| 22 months \| \| Australian before-and-after study \| N=31, type I, II or III SMA \| Before-and-after study \| 12 months \| \| Israeli before-and-after study \| N=20, type I SMA \| Single-centre before-and-after study \| 2 years \| \| Slovenian before-and-after study \| N=15, type 2 SMA \| Single-centre before-and-after study \| 12 months \| \| Italian before-and-after study \| N=8, type I or 2 SMA \| Before-and-after study \| 6 months \| \| Canadian registry study \| N=146, type I, II or III SMA \| Before-and-after study, multicentre \| 12 months (type I), 4.8 months (type II), 7.5 months (type III) (median) \| \| Japanese before-and-after study \| N=11, type I, II or III SMA \| Before-and-after study, single centre \| 9-12 months \| \| Brazilian before-and-after study (type I) \| N=21, type I SMA \| Before-and-after study, single centre \| 6-24 months \| \| Brazilian before-and-after study (type II/III) \| N=41, type II or III SMA \| Before-and-after study, single centre \| 6-24 months \| \| Russian before-and-after study \| N=41, type I SMA \| Before-and-after study, multicentre \| 6 months \| \| German before-and-after study \| N=12, type II/III SMA \| Before-and-after study, single centre \| 180-300 days \| \| Hungarian before-and-after study \| N=54, type I, II or III SMA \| Before-and-after study, multicentre \| 307-551 days \| \| French before-and-after study \| N=24, type II SMA \| Multicentre cohort with historical control \| Till after 6 injections \| | | | |

**Number of patients in each trial by SMA type.**

|  | **Type I** | **Type II/III** | **presymptomatic** | **Type I and II** | **Type I, II, III** |
| --- | --- | --- | --- | --- | --- |
|  | 122 | 126 | 25 | 12 | 292 |
|  | 20 | 21 |  | 31 | 146 |
|  | 68 | 77 |  | 8 | 11 |
|  | 20 | 15 |  |  | 54 |
|  | 20 | 41 |  |  |  |
|  | 21 | 12 |  |  |  |
|  | 41 | 24 |  |  |  |
| **Total number of patients** | **312** | **316** | **25** | **51** | **503** |

| Treatment | Tisagenlecleucel | | | |
| --- | --- | --- | --- | --- |
| Evidence rating | B+ | | | |
| Population | ***Indication***  Children and young adult patients (3–25 years old) with confirmed relapsed/refractory B-cell acute lymphoblastic leukaemia (ALL). | | ***Incidence/eligible population***  Incidence of paediatric ALL: 2.9–3.5/100,000 population [91]  10–15% have relapsed/refractory disease [92] | |
| Intervention | **Dose**  Patients ≤50 kg 0.2–5.0 × 10^6^ CAR-T/kg body weight  Patients >50 kg: 0.1–2.5 × 10^8^ CAR-T (non-weight based) | **Frequency**  Single infusion | **Duration**  Single infusion | **Stopping criteria**  Not applicable |
| Comparator | Blinatumomab- or inotuzumab-based therapy  Salvage chemotherapy or clofarabine with intention to proceed to allogenic SCT | | | |
| Outcomes | **B2101J [91]**   \| Primary outcome—safety/feasibility \| 73 patients enrolled, 62 patients infused  89% had cytokine release syndrome \| \| --- \| --- \| \| Key secondary outcome—overall remission (CR+CRi) \| 95% at 28 days  First paediatric patient treated has been in remission for 5 years \| \| Key secondary outcome—EFS \| 74% at 6 months \| \| Outcome of interest—OS (secondary) \| 86% at 12 months \| \| Outcome of interest—QoL \| NR \|   **ENSIGN (B2205J) [91]**   \| Primary outcome—overall remission (CR+CRi) at 6 months \| 69% BOR lasting for  at least 28 days during 6 months after infusion. \| \| --- \| --- \| \| Key secondary outcome—EFS \| 55% at 6 months \| \| Outcome of interest—OS (secondary) \| 76% at 12 months \| \| Outcome of interest—QoL \| NA \| \| Safety \| Severe (grade 3/4) AEs: NR \|   **ELIANA (B2202) [93, 94]**   \| Primary outcome—overall remission (CR+CRi) at 3 months \| 81% at 3 months maintained for at least 28 days \| \| --- \| --- \| \| Key secondary outcome—EFS \| 73% at 6 months \| \| Outcome of interest—OS (secondary) \| 76% at 12 months \| \| Outcome of interest—QoL \| *Mean change from baseline to month 3*  PedsQL total score 13.3 (95% CI 8.9–17.6)  EQ-5D VAS 16.8 (9.4–24.3) \| \| Safety \| Severe (grade 3/4) AEs: >30% (88%) \|   **French RWE [95]**   \| Primary outcome—overall remission (CR+CRi) \| 95% at 1 month (55 patients enrolled, 41 patients infused) \| \| --- \| --- \| \| Key secondary outcome—EFS \| 58% (95% CI: 37, 74) at 18 months (median EFS not yet reached) \| \| Outcome of interest—OS (secondary) \| 80% (95% CI: 58, 92) at 18 months (median OS not yet reached) \| \| Outcome of interest—QoL \| NR \| \| Safety \| Severe (grade 3/4) AEs: >30% (54%) \|   **Canada/US registry study [96]**   \| Primary outcome—complete response \| 85.5% initial response \| \| --- \| --- \| \| Key secondary outcome—EFS \| 68.6% (95% CI: 62.0, 74.4) at 6 months and 52.4 (95% CI: 43.4, 60.7) at 12 months \| \| Outcome of interest—OS (secondary) \| 6 months: 88.5% (95% CI: 83.6, 92.0)  12 months: 77.2% (95% CI: 69.8, 83.1) \| \| Outcome of interest—QoL \| NR \| \| Safety \| Severe (grade 3/4) AEs: >30% (41%) \|   **US retrospective study [97]**   \| Primary outcome—complete response \| 85% at 1 month \| \| --- \| --- \| \| Key secondary outcome—EFS \| 64% at 6 months and 51% at 12 months \| \| Outcome of interest—OS (secondary) \| 85% at 6 months and 72% at 12 months \| \| Outcome of interest—QoL \| NR \| \| Safety \| Severe (grade 3/4) AEs: NR \|   **UK retrospective study [98]**   \| Primary outcome——overall remission (CR+CRi) \| 95% in the first 90 days (on an ITT basis CR/Cri rate was 84.8%) \| \| --- \| --- \| \| Key secondary outcome—EFS \| 74.8% at 6 months and 68.2% at 12 months (median EFS not yet reached) \| \| Outcome of interest—OS (secondary) \| 97.6% at 6 months and 86.1% at 12 months (median OS not yet reached) \| \| Outcome of interest—QoL \| NR \| \| Safety \| Severe (grade 3/4) AEs: CRS, neurotoxicity, infection or cytopenia after day 30 post infusion occurred in 20.4%,10.2% 27.1% and 54.2% respectively \|   **B2001X [99]**   \| Primary outcome— Primary outcome——overall remission (CR+CRi) \| N=73 enrolled, N=67 infused  85% (74, 92) at 3 months \| \| --- \| --- \| \| Key secondary outcome—DOR \| 83% (69, 91) at 6 months \| \| Outcome of interest—QoL \| NR \| \| Safety \| Severe (grade 3/4) AEs: NR \| | | | |
| Study designs | \| Trial \| Patients \| Design \| Follow-up \| \| --- \| --- \| --- \| --- \| \| B2101J \| N=73 enrolled, n=62 infused (n=56 meeting indication), age 1–24, CD19+ B cell malignancies \| Single centre phase I/II single-arm study, cumulative dosing over 3 days \| >3 years \| \| ENSIGN (NCT02228096) \| N=75 enrolled, n=64 infused, paediatric patients with r/r B-cell ALL and B-cell lymphoblastic lymphoma \| Multicentre, phase II,  single-arm, open-label study \| Median 19.6 months \| \| ELIANA (NCT02435849) \| N=92 enrolled, n=75 infused \| Multicentre, phase II,  single-arm, open-label study (registrational) \| Median 13.1 months \| \| French RWE \| N=55 enrolled, n=41 infused \| Multicentre, single arm, open-label study \| Median 7.2 months \| \| Canada/US registry \| N=255 analysed \| Multicentre, single arm, open-label study (registrational) \| Median 13.4 months \| \| US retrospective study \| N=200 enrolled, N=185 infused \| Multicentre, single arm, open-label, retrospective study \| Median 11.2 months \| \| UK retrospective study \| N=66 enrolled, N=49 infused \| Multicentre, single arm, open-label, retrospective study \| Median 9.9 months from infusion \| \| B2001X (NCT03123939) \| N=73 enrolled, N=67 infused \| Multicentre, global study (including patients with prior anti-CD19 therapy after enrolment ended in the ELIANA study) \| Median 9.6 months \| | | | |

| Treatment | Velmanase alfa | | | |
| --- | --- | --- | --- | --- |
| Evidence rating | C+ | | | |
| Population | ***Indication***  Patients with mild to moderate alpha-mannosidosis | | ***Incidence/eligible population***  Incidence <0.1–1.5/100,000 live births [100]  Prevalence 0.2/100,000 population [101]  Most patients have clinically moderate disease [102] | |
| Intervention | **Dose**  1 mg/kg of body weight by IV infusion | **Frequency**  Once weekly | **Duration**  Ongoing | **Stopping criteria**  Lack of clear benefit |
| Comparator | No effective treatment. HSCT in some patients (generally <5 years old) | | | |
| Outcomes | **Study rhLAMAN-05[103] [104]**   \| Co-primary outcome—serum oligosaccharides \| *Adjusted mean difference in change from baseline to 52 weeks, velmanase alfa vs. placebo:*  Relative: −70.5% (95% CI: −78.4, −59.7), p<0.001  The change in the velmanase alfa arm was -77.6%  Absolute: −3.50 μmol/L (95% CI: −4.37, −2.62), p<0.001 \| \| --- \| --- \| \| Co-primary outcome—3MSCT \| *Adjusted mean difference in change from baseline to 52 weeks, velmanase alfa vs. placebo:*  Relative: 3.0% (95% CI: –9.86, 17.7), p=0.648  Absolute: −1.1% (95% CI –9.0 to 7.6) versus 0.0% (95% CI –13.4 to 6.5). \| \| Key secondary outcome—6MWT \| *Adjusted mean difference in change from baseline to week 52, velmanase alfa vs. placebo:*  Relative: 1.9% (95% CI: –6.6,11.1), p=0.66  Absolute: 3.7 (−20.3 to 27.8) versus –3.6 (−33.1 to 25.9) \| \| Key secondary outcome—FVC \| *Adjusted mean difference in change from baseline to week 52, velmanase alfa vs. placebo:*  Relative: 8.4% (95% CI: –6.1, 25.1), p=0.27  Absolute: 8.2 (1.8 to 14.6) versus 2.3 (−6.2 to 10.8) \| \| Outcome­—response (not clearly defined) \| *In the 12-month, phase III, randomized, placebo-controlled trial:*  Treatment: 93% were responders  Placebo: 0% were responders  *In the open-label treatment extension phase:*  Previously treated patients confirmed their global response to VA (92%) after a median follow-up of 29.3 months  Former placebo patients switched to VA (n = 9) increased their response rate to 100% \| \| Outcome of interest—OS \| NR \| \| Outcome of interest—QoL \| NR \| \| Safety \| Severe (grade 3/4) AEs: <30% (velmanase alfa 7% vs. placebo 0%) \|   **Study rhLAMAN-10 [105-107]**   \| Co-primary outcome—serum oligosaccharides \| Mean change from baseline to last observation: –62.76% (95% CI: –74.68, –50.85), p<0.001  Mean absolute value: –4.59 (95% CI: –5.74, –3.45), p<0.001 \| \| --- \| --- \| \| Co-primary outcome—3MSCT \| Mean change from baseline to last observation: 13.77% (95% CI: 4.61, 22.92), p=0.004  Mean absolute value: 6.384 (95% CI: 2.65, 10.12), p=0.001 \| \| Secondary outcome—6MWT \| Mean change at 48 months: 69.7% (95% CI: 7.4, 132), p=0.033  Mean absolute value at 48 months: 22.5 (95% CI: –5.0, 50.0), p=0.096 \| \| Secondary outcome—FVC \| Mean change: 10.5% (95% CI: 2.6, 18.5), p=0.011  Mean absolute value: 8.1 (95% CI: 2.4, 13.7), p=0.007 \| \| Outcome of interest—OS \| NR \| \| Outcome of interest—QoL \| 75.8% (25/33) of caregivers reported that frequent infections are an important clinical problem of AM that impacted patients' social interactions and QoL. During the post-VA-treatment period, 88.0% (22/25) of caregivers reported a reduction in patient infections.  Pre-treatment joint pain (51.5% [17/33]), walking difficulty (72.7% [24/33]), dexterity problems (60.6% [20/33]), and mental delay  (90.9% [30/33]).  Post-VA-treatment, caregivers reported greater patient independence, measured by improved dexterity (55.0% [11/20]), reduced joint pain (58.8% [10/17]), and improved walking ability (66.7% [16/24]). 66.7% (20/30) of caregivers noticed an improvement in mental delay, which included reports of increased empathy, ability to understand their surrounding environment, or improved vocabulary. \| \| Safety \| Severe (grade 3/4) AEs: NR \|   **Long-term follow-up [106, 108-110]**   \| Co-primary outcome—serum oligosaccharides \| Mean change from baseline to last observation: −62.8% (95% CI: –74.7,  -50.8), p<0.001 \| \| --- \| --- \| \| Co-primary outcome—3MSCT \| Mean change from baseline to last observation: +13.8% (95% CI: 4.61, 22.92), p=0.004 \| \| Secondary outcome—6MWT \| Mean change 7.1% (95% CI: –0.7, 14.9), p=0.071  Mean absolute value 22.4 metres (95% CI: 0.0, 44.8), p=0.050 \| \| Secondary outcome—FVC \| Mean change 28.4% (95% CI: 14.0, 42.8), p<0.001  Mean absolute value 0.6 L (95% CI: 0.3, 0.9), p<0.001 \| \| Outcome of interest—OS \| NR \| \| Outcome of interest—QoL \| *Childhood Health Assessment Questionnaire Disability Index (CHAQ-DI):*  Change from baseline to 12 months: -0.10 (-0.23 to 0.03)  Change from baseline to last observation: -0.13 (-0.29 to 0.02)  *Childhood Health Assessment Questionnaire VAS Pain:*  The mean change value (SD) from baseline to month 12 was 0.148 (0.723) and baseline to LO was -0.173 (0.647), a mean (SD) percentage change from baseline of 3.697% (107.3) at 12 months and -17.0% (109.8%) at LO.  In the paediatric group, the change at 12 months was -13.7% and -0.4% at LO.  *Q-5D-5L Health Index:*  Mean (SD) absolute change from baseline was 0.0346 (0.1044) at 12 months and 0.050 (0.135) at LO; mean (SD) percentage change of 6.932 (19.098) at 12 months and 11.23% (24.72) at LO.  Pediatric EQ-5D-5L health index values changed from baseline to 12 months by -0.513% (8.538%) and increased to LO from by 17.49% (28.27).  *EQ-5D-5L VAS for Best Health:*  Increase in the mean score (SD) at LO of 3.3 (18.1). \| \| Safety \| Severe (grade 3/4) AEs:<30% (:9%) \| | | | |
| Study designs | \| Trial \| Patients \| Design \| Follow-up \| \| --- \| --- \| --- \| --- \| \| Study rhLAMAN-05 (NCT01681953) \| N=25 \| Multicentre, phase III RCT, double-blind, placebo-controlled \| 12 months \| \| Study rhLAMAN-10 (NCT02478840) \| N=33 \| Single arm, open label, “integrated database”, including several small single-arm cohort studies \| 48 months \| | | | |
| Treatment | Vestronidase alfa | | | |
| Evidence rating | I | | | |
| Population | ***Indication***  Mucopolysaccharidosis VII (MPS VII, Sly syndrome) | | ***Incidence/eligible population***  Incidence 0.4/100,000 live births [101]  Prevalence <0.1/100,000 population [111] | |
| Intervention | **Dose**  4 mg/kg of body weight IV infusion | **Frequency**  Every 2 weeks | **Duration**  Ongoing | **Stopping criteria**  Loss of efficacy, adverse events, end of life |
| Comparator | No alternative (haematopoietic stem-cell transplant therapy sometimes used, with limited evidence) | | | |
| Outcomes | **Study 301 [111, 112]**   \| Primary outcome—Urinary GAG excretion \| Mean reduction from baseline to week 24: 64.8% (95% CI –69.66, –59.98) p<0.0001.  Reduction in chondroitin sulfate (LS mean change of 70.6% [GEE analysis; p < 0.0001]) \| \| --- \| --- \| \| Key secondary outcome—MDRI \| Mean improvement at week 24 vs. placebo: +0.5 domains, p=0.0016  83.3% (10/12) had a clinically meaningful improvement in at least one MDRI domain: 6MWT, FVCl, shoulder flexion, visual acuity, and Bruininks-Oseretsky Test of Motor Proficiency (BOT-2) \| \| Key secondary outcome—6MWT \| Mean change from baseline to week 24: 20.8 metres; p=0.2137 \| \| Key secondary outcome—Visual acuity \| *LSM change from baseline to week 24:*  Right eye: 1 line; p=0.1140  Left eye: 0.9 line; p=0.906 \| \| Key secondary outcome—Fine Motor Precision \| Mean –0.2; p=0.3528 \| \| Outcome of interest—OS \| NR \| \| Outcome of interest—QoL \| Fatigue Total Score (PedsQL): mean 3.4; p=0.1953  75% (9/12) of patients improved over baseline in fatigue at some point during the study \| \| Safety \| Severe (grade 3/4) AEs: NR \|   **Study 301/202 [113]**   \| Primary outcome—Urinary GAG \| *LSM change from baseline*  −62% at week 0 of extension study  −58% at week 48 of extension study \| \| --- \| --- \| \| Key secondary outcome—MDRI \| *Mean improvement*  +0.7 at week 24  +0.9 at week 48 \| \| Outcome of interest—OS \| NR \| \| Outcome of interest—QoL \| NR \| \| Safety \| Severe (grade 3/4) AEs:<30% (25%) \|   **Study 203[114] [115]**   \| Primary outcome—Urinary GAG \| LSM decrease from baseline: 64% (LS mean 60%) at week 4 (p<0.0001), sustained to week 48 (LS mean 61%) \| \| --- \| --- \| \| Key secondary outcome—Standing height \| Mean increase  86 cm at baseline to 92 cm at week 48  Z score stable from baseline (mean −2.27) to week 48 (−2.12) \| \| Key secondary outcome—Growth velocity \| Mean velocity increased from 5.06 cm/year in 2 years before treatment to 6.84 cm/year following treatment \| \| Key secondary outcome— Hepatosplenomegaly by ultrasound \| Hepatomegaly n=3 at baseline, resolved in 3 patients at week 48  Splenomegaly n=3 at baseline, resolved in 1 patient at week 48 \| \| Outcome of interest—OS \| NR \| \| Outcome of interest—QoL \| NR \| \| Safety \| Severe (grade 3/4) AEs: NR (37.5% treatment related SAEs) \| | | | |
| Study designs | \| Trial \| Patients \| Design \| Follow-up \| \| --- \| --- \| --- \| --- \| \| Study 301 (NCT02230566) \| N=12 \| Multicentre, phase III blind-start, single crossover, placebo-controlled \| 48 weeks \| \| Study 202 (NCT02432144) \| N=12 \| Single arm extension of study 301 \| 144 weeks \| \| Study 203 (NCT02418455) \| N=8 \| Phase II, open-label, single arm \| 240 weeks \| | | | |

**R****eferences**

1. European Medicines Agency Committee for Medicinal Products for Human Use (CHMP). CHMP assessment report: Crysvita (EMEA/H/C/004275/0000) London: European Medicines Agency; 2018 [Available from: <https://www.ema.europa.eu/en/medicines/human/EPAR/crysvita>.]

2. Imel E, Carpenter T, Gottesman G, Chen A, Skrinar A, Roberts MS, et al. Three-Year Safety and Efficacy Results of Burosumab for Children Aged 1 to 4 years with X-linked Hypophosphatemia (XLH). J Bone Miner Res. 2020;35:16.

3. Padidela R, Whyte MP, Glorieux FH, Munns CF, Ward LM, Nilsson O, et al. Patient-Reported Outcomes from a Randomized, Active-Controlled, Open-Label, Phase 3 Trial of Burosumab Versus Conventional Therapy in Children with X-Linked Hypophosphatemia. Calcif Tissue Int. 2021;108(5):622-33.

4. Imel EA, Glorieux FH, Whyte MP, Munns CF, Ward LM, Nilsson O, et al. Burosumab versus conventional therapy in children with X-linked hypophosphataemia: a randomised, active-controlled, open-label, phase 3 trial. Lancet. 2019;393(10189):2416-27.

5. Carpenter TO, Högler W, Imel EA, Portale AA, Boot AM, Linglart A, et al. Continued improvement in clinical outcomes with long-term burosumab, a fully human anti-FGF23 monoclonal antibody: results from a 3-year, phase 2, clinical trial in children with X-linked hypophosphatemia (XLH). J Bone Miner Res. 2019;34:Abstract 1037.

6. Whyte MP, Carpenter TO, Gottesman GS, Mao M, Skrinar A, San Martin J, et al. Efficacy and safety of burosumab in children aged 1-4 years with X-linked hypophosphataemia: a multicentre, open-label, phase 2 trial. Lancet Diabetes Endocrinol. 2019;7(3):189-99.

7. Brener A, Lebenthal Y, Cleper R, Kapusta L, Zeitlin L. Body composition and cardiometabolic health of pediatric patients with X-linked hypophosphatemia (XLH) under burosumab therapy. Ther Adv Endocrinol Metab. 2021;12:20420188211001150-.

8. National Organization for Rare Diseases. Rare Disease Database: Lennox-Gastaut Syndrome Danbury CT National Organization for Rare Diseases; 2020 [Available from: https://rarediseases.org/rare-diseases/lennox-gastaut-syndrome/.]

9. Orphanet. Dravet syndrome Paris, France: Orphanet; 2020 [Available from: https://www.orpha.net/consor/cgi-bin/OC_Exp.php?Expert=33069.]

10. Auvin S, Irwin J, Abi-Aad P, Battersby A. The problem of rarity: estimation of prevalence in rare disease. Value Health. 2018;21(5):501-7.

11. European Medicines Agency Committee for Medicinal Products for Human Use (CHMP). CHMP assessment report: Epidyolex (EMEA/H/C/004675/0000) London: European Medicines Agency; 2019. [Available from: <https://www.ema.europa.eu/en/medicines/human/EPAR/epidyolex>.]

12. Devinsky O, Cross JH, Laux L, Marsh E, Miller I, Nabbout R, et al. Trial of cannabidiol for drug-resistant seizures in the Dravet syndrome. N Engl J Med. 2017;376(21):2011-20.

13. Miller I, Scheffer IE, Gunning B, Sanchez-Carpintero R, Gil-Nagel A, Perry MS, et al. Dose-ranging effect of adjunctive oral cannabidiol vs placebo on convulsive seizure frequency in Dravet syndrome: a randomized clinical trial. JAMA Neurol. 2020;77(5):613-21.

14. Halford JJ, Scheffer I, Nabbout R, Sanchez-Carpintero R, Malawky YS, Wong M, et al. Long-term safety and efficacy of cannabidiol (CBD) treatment in patients with Dravet syndrome (DS): 3-year interim results of an open-label extension (OLE) trial (GWPCARE5). Neurol. 2020;94:Abstract 439.

15. Devinsky O, Patel AD, Cross JH, Villanueva V, Wirrell EC, Privitera M, et al. Effect of cannabidiol on drop seizures in the Lennox-Gastaut syndrome. N Engl J Med. 2018;378(20):1888-97.

16. Thiele EA, Marsh ED, French JA, Mazurkiewicz-Beldzinska M, Benbadis SR, Joshi C, et al. Cannabidiol in patients with seizures associated with Lennox-Gastaut syndrome (GWPCARE4): a randomised, double-blind, placebo-controlled phase 3 trial. Lancet. 2018;391(10125):1085-96.

17. Patel A, Chin R, Mitchell W, Perry S, Weinstock A, Checketts D, et al. Long-term safety and efficacy of cannabidiol (CBD) treatment in patients with Lennox Gastaut syndrome (LGS): 3-year results of an open-label extension (OLE) trial (GWPCARE5). Neurol. 2020;94:Abstract 668.

18. Privitera M, Bhathal H, Wong M, Cross JH, Wirrell E, Marsh ED, et al. Time to onset of cannabidiol (CBD) treatment effect in Lennox-Gastaut syndrome: Analysis from two randomized controlled trials. Epilepsia. 2021;62(5):1130-40.

19. Laux LC, Bebin EM, Checketts D, Chez M, Flamini R, Marsh ED, et al. Long-term safety and efficacy of cannabidiol in children and adults with treatment resistant Lennox-Gastaut syndrome or Dravet syndrome: Expanded access program results. Epilepsy Res. 2019;154:13-20.

20. Devinsky O, Thiele EA, Wright S, Checketts D, Morrison G, Dunayevich E, et al. Cannabidiol efficacy independent of clobazam: Meta-analysis of four randomized controlled trials. Acta Neurol Scand. 2020;142(6):531-40.

21. Hassan E, Desai I, Soponski R, Muthugovindan D, Tan HJ. UK North West profile of Epidyolex use for refractory seizures in children. Dev Med Child Neurol. 2021;63:92.

22. Koo CM, Kim SH, Lee JS, Park BJ, Lee HK, Kim HD, et al. Cannabidiol for Treating Lennox-Gastaut Syndrome and Dravet Syndrome in Korea. J Korean Med Sci. 2020;35(50):e427-e.

23. Salen G, Steiner RD. Epidemiology, diagnosis, and treatment of cerebrotendinous xanthomatosis (CTX). J Inherit Metab Dis. 2017;40(6):771-81.

24. Zübarioğlu T, Bilen İ P, Kıykım E, Doğan BB, Enver E, Cansever M, et al. Evaluation of the effect of chenodeoxycholic acid treatment on skeletal system findings in patients with cerebrotendinous xanthomatosis. Turk Pediatri Ars. 2019;54(2):113-8.

25. Stelten BML, Huidekoper HH, van de Warrenburg BPC, Brilstra EH, Hollak CEM, Haak HR, et al. Long-term treatment effect in cerebrotendinous xanthomatosis depends on age at treatment start. Neurol. 2019;92(2):e83-e95.

26. Verrips A, Dotti MT, Mignarri A, Stelten BML, Verma S, Federico A. The safety and effectiveness of chenodeoxycholic acid treatment in patients with cerebrotendinous xanthomatosis: two retrospective cohort studies. Neurol Sci. 2020;41(4):943-9.

27. Amador MDM, Masingue M, Debs R, Lamari F, Perlbarg V, Roze E, et al. Treatment with chenodeoxycholic acid in cerebrotendinous xanthomatosis: clinical, neurophysiological, and quantitative brain structural outcomes. J Inherit Metab Dis. 2018;41(5):799-807.

28. Duell PB, Salen G, Eichler FS, DeBarber AE, Connor SL, Casaday L, et al. Diagnosis, treatment, and clinical outcomes in 43 cases with cerebrotendinous xanthomatosis. J Clin Lipidol. 2018;12(5):1169-78.

29. European Medicines Agency Committee for Medicinal Products for Human Use (CHMP). CHMP assessment report: Brineura (EMEA/H/C/004065/0000) London: European Medicines Agency; 2017. [Available from: <https://www.ema.europa.eu/en/medicines/human/EPAR/brineura>.]

30. Schulz A, Ajayi T, Specchio N, de Los Reyes E, Gissen P, Ballon D, et al. Study of intraventricular Cerliponase Alfa for CLN2 Disease. N Engl J Med. 2018;378(20):1898-907.

31. Schulz A, Specchio N, Gissen P, de los Reyes E, Slasor P, Jacoby D. Persistent treatment effect of cerliponase alfa in children with CLN2 disease: A > 4 year update from an ongoing multicenter extension study. Mol Gen Metab. 2020;129(2):S145.

32. Wibbeler E, Wang R, Reyes EdL, Specchio N, Gissen P, Guelbert N, et al. Cerliponase Alfa for the treatment of atypical phenotypes of CLN2 disease: A Retrospective Case Series. J Child Neurol. 2021;36(6):468-74.

33. European Medicines Agency Committee for Medicinal Products for Human Use (CHMP). CHMP assessment report: Qarziba (EMEA/H/C/003918/0000) London: European Medicines Agency; 2017. [Available from: https://www.ema.europa.eu/en/medicines/human/EPAR/qarziba.]

34. Orphanet. Neuroblastoma Paris, France: Orphanet; 2009 [Available from: <https://www.orpha.net/consor/cgi-bin/OC_Exp.php?Lng=GB&Expert=635>.]

35. Ladenstein R, Pötschger U, Valteau-Couanet D, Luksch R, Castel V, Yaniv I, et al. Interleukin 2 with anti-GD2 antibody ch14.18/CHO (dinutuximab beta) in patients with high-risk neuroblastoma (HR-NBL1/SIOPEN): a multicentre, randomised, phase 3 trial. Lancet Oncol. 2018;19(12):1617-29.

36. Ladenstein R, Pötschger U, Pearson ADJ, Brock P, Luksch R, Castel V, et al. Busulfan and melphalan versus carboplatin, etoposide, and melphalan as high-dose chemotherapy for high-risk neuroblastoma (HR-NBL1/SIOPEN): an international, randomised, multi-arm, open-label, phase 3 trial. Lancet Oncol. 2017;18(4):500-14.

37. Ladenstein RL, Poetschger U, Valteau-Couanet D, Gray J, Luksch R, Balwierz W, et al. Randomization of dose-reduced subcutaneous interleukin-2 (scIL2) in maintenance immunotherapy (IT) with anti-GD2 antibody dinutuximab beta (DB) long-term infusion (LTI) in front-line high-risk neuroblastoma patients: Early results from the HRNBL1/ SIOPEN trial. J Clin Oncol. 2019;37(Supplement 15).

38. Ladenstein RL, Poetschger U, Valteau-Couanet D, Gray J, Luksch R, Balwierz W, et al. Risk factors in the HR-NBL-1/SIOPEN study in patients receiving dinutuximab beta (DB) based immunotherapy. J Clin Oncol. 2020;38(15).

39. Ladenstein RL, Poetschger U, Couanet DV, Gray J, Luksch R, Castel V, et al. Immunotherapy with anti-GD2 antibody ch14.18/CHO+/-IL2 within the HRNBL1/ SIOPEN trial to improve outcome of high-risk neuroblastoma patients compared to historical controls. J Clin Oncol. 2018;36(15 Supplement 1).

40. Ladenstein R, Pötschger U, Valteau-Couanet D, Luksch R, Castel V, Ash S, et al. Investigation of the role of dinutuximab beta-based immunotherapy in the SIOPEN High-Risk Neuroblastoma 1 Trial (HR-NBL1). Cancers (Basel). 2020;12(2):309.

41. Holmes K, Poetschger U, Sarnacki S, Monclair T, Cecchetto G, Gomez Chacon J, et al. The influence of surgical excision on survival in high-risk neuroblastoma revisited after introduction of ch14.18/CHO immunotherapy in the HR-NBL1/SIOPEN trial. J Clin Oncol. 2018;36(15_suppl):10521-.

42. Holmes K, Potschger U, Pearson ADJ, Sarnacki S, Cecchetto G, Gomez-Chacon J, et al. Influence of Surgical Excision on the Survival of Patients With Stage 4 High-Risk Neuroblastoma: A Report From the HR-NBL1/SIOPEN Study. J Clin Oncol. 2020;38(25):2902-15.

43. Wex J, Zibelnik N, Zemam A. Dinutuximab beta with isotretinoin versus isotretinoin alone in the treatment of high-risk neuroblastoma: impact on long-term survival. Value Health. 2019;22:S435 (abstract PCN4).

44. Lode HN, Valteau-Couanet D, Gray J, Luksch R, Wieczorek A, Castel V, et al. Randomized use of anti-GD2 antibody dinutuximab beta (DB) long-term infusion with and without subcutaneous interleukin-2 (scIL-2) in high-risk neuroblastoma patients with relapsed and refractory disease: Results from the SIOPEN LTI-trial. J Clin Oncol. 2019;37:10014-.

45. Lode H, Jensen C, Siebert N, Kietz S, Ehlert K, Müller I, et al. Immune activation, clinical response and survival following long-term infusion of anti-GD2 antibody ch14.18/CHO in combination with interleukin-2 in high-risk neuroblastoma patients. Cancer Res. 2014;74(19):CT410.

46. Siebert N, Troschke-Meurer S, Marx M, Zumpe M, Ehlert K, Gray J, et al. Impact of HACA on Immunomodulation and Treatment Toxicity Following ch14.18/CHO Long-Term Infusion with Interleukin-2: Results from a SIOPEN Phase 2 Trial. Cancers (Basel). 2018;10(10):387.

47. Mueller I, Ehlert K, Endres S, Pill L, Siebert N, Kietz S, et al. Tolerability, response and outcome of high-risk neuroblastoma patients treated with long-term infusion of anti-GD(2) antibody ch14.18/CHO. MAbs. 2018;10(1):55-61.

48. Orphanet. Permanent neonatal diabetes mellitus Paris, France: Orphanet; 2014 [Available from: https://www.orpha.net/consor/cgi-bin/OC_Exp.php?Lng=GB&Expert=99885.]

49. European Medicines Agency Committee for Medicinal Products for Human Use (CHMP). CHMP assessment report: Amglidia (EMEA/H/C/004379/0000) London: European Medicines Agency; 2018 [Available from: https://www.ema.europa.eu/en/medicines/human/EPAR/amglidia.]

50. Pearson ER, Flechtner I, Njølstad PR, Malecki MT, Flanagan SE, Larkin B, et al. Switching from insulin to oral sulfonylureas in patients with diabetes due to Kir6.2 mutations. N Engl J Med. 2006;355(5):467-77.

51. Beltrand J, Elie C, Busiah K, Fournier E, Boddaert N, Bahi-Buisson N, et al. Sulfonylurea Therapy Benefits Neurological and Psychomotor Functions in Patients With Neonatal Diabetes Owing to Potassium Channel Mutations. Diabetes Care. 2015;38(11):2033-41.

52. Beltrand J, Baptiste A, Busiah K, Bouazza N, Godot C, Boucheron A, et al. Glibenclamide oral suspension: Suitable and effective in patients with neonatal diabetes. Pediatr Diabetes. 2019;20(3):246-54.

53. Bowman P, Sulen Å, Barbetti F, Beltrand J, Svalastoga P, Codner E, et al. Effectiveness and safety of long-term treatment with sulfonylureas in patients with neonatal diabetes due to KCNJ11 mutations: an international cohort study. Lancet Diabetes Endocrinol. 2018;6(8):637-46.

54. European Medicines Agency Committee for Medicinal Products for Human Use (CHMP). CHMP assessment report: Myalepta (EMEA/H/C/004218/0000) London: European Medicines Agency; 2018 [Available from: https://www.ema.europa.eu/en/medicines/human/EPAR/myalepta.]

55. Chiquette E, Oral EA, Garg A, Araújo-Vilar D, Dhankhar P. Estimating the prevalence of generalized and partial lipodystrophy: findings and challenges. Diabetes, Metabolic Syndrome and Obesity: Targets Ther. 2017;10:375.

56. Brown RJ, Oral EA, Cochran E, Araújo-Vilar D, Savage DB, Long A, et al. Long-term effectiveness and safety of metreleptin in the treatment of patients with generalized lipodystrophy. Endocrine. 2018;60(3):479-89.

57. Cook K, Stears A, Araujo-Vilar D, Santini F, Stephen O, Ceccarini G, et al. Real-world experience of generalized and partial lipodystrophy patients enrolled in the metreleptin early access program. Endocrine Abstracts. 2019;63:586.

58. European Medicines Agency Committee for Medicinal Products for Human Use (CHMP). CHMP assessment report: Spinraza (EMEA/H/C/004312/0000) London: European Medicines Agency; 2017 [Available from: https://www.ema.europa.eu/en/medicines/human/EPAR/spinraza.]

59. National Organization for Rare Diseases. Rare Disease Database: Spinal Muscular Atrophy. Danbury, CT: National Organization for Rare Diseases; 2012 [Available from: https://rarediseases.org/rare-diseases/spinal-muscular-atrophy/.]

60. Finkel RS, Mercuri E, Darras BT, Connolly AM, Kuntz NL, Kirschner J, et al. Nusinersen versus Sham Control in Infantile-Onset Spinal Muscular Atrophy. N Engl J Med. 2017;377(18):1723-32.

61. Castro D, Finkel RS, Farrar MA, Tulinius M, Krosschell KJ, Saito K, et al. Nusinersen in infantile-onset spinal muscular atrophy: results from longer-term treatment from the open-label SHINE extension study. Neurology. 2020;94:1659.

62. Darras BT, De Vivo DC, Farrar MA, Mercuri E, Finkel RS, Foster R, et al. Safety Profile of Nusinersen in Presymptomatic and Infantile-Onset Spinal Muscular Atrophy (SMA): Interim Results From the NURTURE and ENDEAR-SHINE Studies (1659). Neurology. 2020;94(15 Supplement):1659.

63. Finkel R, Castro D, Farrar M, Tulinius M, Krosschell K, Saito K, et al. P.266 Nusinersen in infantile-onset spinal muscular atrophy: results from longer-term treatment from the open-label SHINE extension study. Neuromuscul Dis. 2020;30(S124).

64. Hodgkinson-Brechenmacher V, Oskoui M, Campbell C, Lounsberry J, Brais B, MacKenzie A, et al. P.174 The Canadian neuromuscular disease registry: A national spinal muscular atrophy (SMA) registry for real world evidence. Neuromuscul Dis. 2020;30:S97-S8.

65. Tokunaga S, Shimomura H, Taniguchi N, Lee T, Takeshima Y. Analysis of the therapeutic effect of nusinersen in patients with spinal muscular atrophy using a motor function scale and questionnaire. No To Hattatsu. 2020;52(6):390-6.

66. Mendonça RH, Jorge Polido G, Ciro M, Jorge Fontoura Solla D, Conti Reed U, Zanoteli E. Clinical Outcomes in Patients with Spinal Muscular Atrophy Type 1 Treated with Nusinersen. J Neuromuscul Dis. 2021;8(2):217-224.

67. Artemieva SB, Kuzenkova LM, Ilyina ES, Kursakova YA, Kolpakchi LM, Sapego EY, et al. The efficacy and safety of nusinersen within the expanded access program in Russia. Neuromuscul Disord. 2020;10(3):35-41.

68. Szabo L, Gergely A, Jakus R, Fogarasi A, Grosz Z, Molnar MJ, et al. Efficacy of nusinersen in type 1, 2 and 3 spinal muscular atrophy: Real world data from Hungarian patients. Eur J Paediatr Neurol. 2020;27:37-42.

69. Pane M, Coratti G, Sansone VA, Messina S, Catteruccia M, Bruno C, et al. Type I SMA "new natural history": long-term data in nusinersen-treated patients. Ann Clin Transl Neurol. 2021;8(3):548-57.

70. Edel L, Abbott L, Chan E, Main M, Robinson V, Munot P, et al. SMA - CLINICAL: P.92 Comparing motor and respiratory function in SMA Type1 treated with Nusinersen using CHOP INTEND(CHOP) and Great Ormond Street Respiratory Score(GSR). Neuromuscul Disord. 2020;30:S74.

71. Lavie M, Diamant N, Sadot E, Fatal A, Sagi L, Domany KA, et al. Nusinersen for Spinal Muscular Atrophy type l: Real-World Respiratory Experience. Eur Respir J. 2020;56.

72. Mercuri E, Darras BT, Chiriboga CA, Day JW, Campbell C, Connolly AM, et al. Nusinersen versus sham control in later-onset Spinal Muscular Atrophy. N Engl J Med. 2018;378(7):625-35.

73. Johnson NB, Paradis AD, Naoshy S, Wong J, Montes J, Krasinski DC. Evaluation of nusinersen on impact of caregiver experience and hrqol in later-onset spinal muscular atrophy (SMA): Results from the phase 3 cherish trial. Neurology. 2020;94.

74. Chiriboga CA, Darras BT, Farrar MA, Mercuri E, Kirschner J, Kuntz NL, et al. Longer-term treatment with Nusinersen: Results in later-onset Spinal Muscular Atrophy From the SHINE study. Neurology. 2020;94:P6.007.

75. Mercuri E, Darras B, Chiriboga C, Farrar M, Kirschner J, Kuntz N, et al. P.257 Longer-term treatment with nusinersen: Results in later-onset spinal muscular atrophy from the SHINE study. Neuromuscul Disord. 2020;30:S121.

76. Montes J, Krasinski D, Foster R, Gambino G, Paradis A, Garafalo S, et al. P.269 Impact of Continued Nusinersin treatment on Caregiver Experience and Health-Related Quality of Life in Later-onset SMA: Results From the SHINE Study. Neuromuscul Disord. 2020;30:S125.

77. Coratti G, Pane M, Lucibello S, Pera MC, Pasternak A, Montes J, et al. Age related treatment effect in type II Spinal Muscular Atrophy pediatric patients treated with nusinersen. Neuromuscul Disord. 2021;31(7):596-602.

78. Mendonca RH, Polido GJ, Matsui C, Silva AMS, Solla DJF, Reed UC, et al. Real-World Data from Nusinersen Treatment for Patients with Later-Onset Spinal Muscular Atrophy: A Single Center Experience. J Neuromuscul Dis. 2021;8(1):101-8.

79. Heitschmidt L, Pichlmaier L, Eckerland M, Steindor M, Olivier M, Fuge I, et al. Nusinersen does not improve lung function in a cohort of children with spinal muscular atrophy - A single-center retrospective study. Eur J Paediatr Neurol. 2021;31:88-91.

80. Krivec U, Zver A, Lepej D, Pirs AK, Praprotnik M, Smit SS, et al. Overnight gas exchange and ventilatory support in spinal muscular atrophy type 2 patients after 12 months of nusinersen treatment. Eur Respir J. 2020;56.

81. Gomez-Garcia de la Banda M, Amaddeo A, Khirani S, Pruvost S, Barnerias C, Dabaj I, et al. Assessment of respiratory muscles and motor function in children with SMA treated by nusinersen. Pediatr Pulmonol. 2021;56(1):299-306.

82. Shieh PB, Acsadi G, Mueller-Felber W, Crawford TO, Richardson R, Natarajan N, et al. Safety and efficacy of nusinersen in infants/children with spinal muscular atrophy (SMA): part 1 of the phase 2 EMBRACE study. Neurology. 2018;90:P2.324.

83. Acsadi G, Crawford TO, Muller-Felber W, Shieh PB, Richardson R, Natarajan N, et al. Safety and efficacy of nusinersen in spinal muscular atrophy: The EMBRACE study. Muscle Nerve. 2021;63(5):668-77.

84. Diaconu M, Duru C, Ugonna K, Kulkarni H, Ong M, White K. Respiratory outcomes pre and post nusinersen in children with spinal muscular atrophy (SMA) in a tertiary UK paediatric respiratory and neurology centre. Eur Respir J. 2020;56.

85. Diaconu M, Duru C, Ugonna K, Kulkarni H, White K, Ong M. Respiratory outcomes pre and post nusinersen in children with spinal muscular atrophy (SMA) in a tertiary UK paediatric respiratory and neurology centre. Dev Med Child Neurol. 2021;63.

86. Chacko A, Deegan S, Gauld L, Sly P. Nusinersen stabilises respiratory function in paediatric spinal muscular atrophy. Eur Respir J. 2020;56:1234.

87. Chacko A, Deegan S, Sly P, Gauld L. Stabilization of respiratory function in pediatric spinal muscular atrophy treated with nusinersen. Am J Respir Crit Care Med. 2020;201:A1161.

88. Ortenzi GB, Palmas G, Andresciani E, Garzone AMF, Berardi MA, Siliquini S, et al. Effectiveness of Nusinersen in paediatric patients SMA1 and SMA2. Eur J Hosp Pharm. 2020;27:A109.

89. De Vivo DC, Bertini E, Swoboda KJ, Hwu WL, Crawford TO, Finkel RS, et al. Nusinersen initiated in infants during the presymptomatic stage of spinal muscular atrophy: Interim efficacy and safety results from the Phase 2 NURTURE study. Neuromuscul Disord. 2019;29(11):842-56.

90. Finkel RS, De Vivo DC, Swoboda KJ, Bertini E, Hwu W-L, Foster R, et al. Nusinersen in Infants Who Initiate Treatment in a Presymptomatic Stage of Spinal Muscular Atrophy (SMA): Interim Results From the Phase 2 NURTURE Study (993). Neurology. 2020;94(15 Supplement):993.

91. European Medicines Agency Committee for Medicinal Products for Human Use (CHMP). CHMP assessment report: Kymriah (EMEA/H/C/004090/0000) London:European Medicines Agency; 2018 [Available from: https://www.ema.europa.eu/en/medicines/human/EPAR/kymriah.]

92. Ma Q, Zhang J, O'Brien E, Martin AL, Agostinho AC. Tisagenlecleucel versus historical standard therapies for pediatric relapsed/refractory acute lymphoblastic leukemia. J Comp Ef. Res. 2020;9(12):849-60.

93. Maude SL, Laetsch TW, Buechner J, Rives S, Boyer M, Bittencourt H, et al. Tisagenlecleucel in Children and Young Adults with B-Cell Lymphoblastic Leukemia. N Engl J Med. 2018;378(5):439-48.

94. Laetsch TW, Myers GD, Baruchel A, Dietz AC, Pulsipher MA, Bittencourt H, et al. Patient-reported quality of life after tisagenlecleucel infusion in children and young adults with relapsed or refractory B-cell acute lymphoblastic leukaemia: a global, single-arm, phase 2 trial. Lancet Oncol. 2019;20(12):1710-8.

95. Dourthe M-E, Rabian F, Yakouben K, Cabannes A, Chevillon F, Chaillou D, et al. Safety and efficacy of Tisagenlecleucel (CTL019) in B-Cell Acute Lymphoblastic Leukemia in children, adolescents and young adults: The French experience. Blood. 2019;134(Supplement_1):3876-.

96. Pasquini MC, Hu Z-H, Curran K, Laetsch T, Locke F, Rouce R, et al. Real-world evidence of tisagenlecleucel for pediatric acute lymphoblastic leukemia and non-Hodgkin lymphoma. Blood Adv. 2020;4(21):5414-24.

97. Schultz LM, Baggott C, Prabhu S, Pacenta H, Phillips CL, Rossoff J, et al. Disease burden impacts outcomes in pediatric and young adult B-Cell Acute Lymphoblastic Leukemia after commercial Tisagenlecleucel: Results from the Pediatric Real World CAR Consortium (PRWCC). Blood. 2020;136(Supplement 1):14-5.

98. Ghorashian S, Furness C, Cummins M, Snowden JA, O'Reilly MA, Roddie C, et al. Intention to treat analysis of real-world outcomes following Tisgenlecleucel therapy for pediatric and young adult ALL through a national access programme. Blood. 2020;136:18-9.

99. Baruchel A, Krueger J, Balduzzi A, Bittencourt H, De Moerloose B, Peters C, et al. Tisagenlecleucel for pediatric/young adult patients with relapsed/refractory b-cell acute lymphoblastic leukemia: preliminary report of b2001x focusing on prior exposure to blinatumomab and inotuzumab (S118). European Hematology Association Library. 2020; 294938; S118

100. Finocchiaro D, Rastelletti I, De-Almeida J, Lloyd S, Gupta P. A Systematic Review of the Epidemiology of Alpha-Mannosidosis. Value Health. 2018;21:S249.

101. National Organization for Rare Diseases. Rare Disease Database: Alpha-Mannosidosis Danbury CT: National Organization for Rare Diseases; 2018 [Available from: https://rarediseases.org/rare-diseases/alpha-mannosidosis/.]

102. Malm D, Nilssen Ø. Alpha-mannosidosis. Orphanet J Rare Dis. 2008;3(1):21.

103. Borgwardt L, Guffon N, Amraoui Y, Dali CI, De Meirleir L, Gil-Campos M, et al. Efficacy and safety of Velmanase alfa in the treatment of patients with alpha-mannosidosis: results from the core and extension phase analysis of a phase III multicentre, double-blind, randomised, placebo-controlled trial. J Inherit Metab Dis. 2018;41(6):1215-23.

104. Hendriksz C, Geelissen S, Rastelletti I, Cattaneo F, Ardigò D, Harmatz P. Velmanase alfa enzyme replacement therapy for alpha-mannosidosis improves patient outcomes over standard of care both in terms of clinically relevant improvement and disease stabilization. Mol Genet Metab. 2020;129:S71-S2.

105. European Medicines Agency Committee for Medicinal Products for Human Use (CHMP). CHMP assessment report: Lamzede (EMEA/H/C/003922/0000) London: European Medicines Agency; 2018 [Available from: https://www.ema.europa.eu/en/medicine/human/EPAR/lamzede.]

106. European Medicines Agency Committee for Medicinal Products for Human Use (CHMP). Assessment report: Lamzede (EMEA/205473/2018) London: European Medicines Agency; 2018 [Available from: https://www.ema.europa.eu/en/documents/assessment-report/lamzede-epar-public-assessment-report_en.pdf.]

107. Lund A, Guffon N, Gil-Campos M, Cattaneo F, Heron B, Tylki-Szymanska A, et al. Effect of velmanase alfa (human recombinant alpha-mannosidase) enzyme-replacement therapy on quality of life and disease burden of patients with alpha-mannosidosis: Results from caregiver feedback. Mol Genet Metab. 2021;132:S67.

108. Borgwardt L, Guffon N, Amraoui Y, Jones SA, De Meirleir L, Lund AM, et al. Health related quality of life, disability, and pain in alpha mannosidosis: Long-term data of enzyme replacement therapy with velmanase alfa (human recombinant alpha mannosidase). J Inborn Errors Metab Screen. 2018;6.

109. Lund AM, Borgwardt L, Cattaneo F, Ardigò D, Geraci S, Gil-Campos M, et al. Comprehensive long-term efficacy and safety of recombinant human alpha-mannosidase (velmanase alfa) treatment in patients with alpha-mannosidosis. J Inherit Metab Dis. 2018;41(6):1225-33.

110. Cattaneo F, Borgwardt L, Dali C, Tylki-Szymanska A, Wijburg F, Van den Hout J, et al. Quality of life and activities of daily living in alpha-mannosidosis: long-term data of enzyme replacement therapy with velmanase alfa (human recombinant alpha mannosidase). J Inborn Errors Metab Screen. 2016;4:60-1.

111. European Medicines Agency Committee for Medicinal Products for Human Use (CHMP). CHMP assessment report: Mepsevii (EMEA/H/C/004438/0000) London: European Medicines Agency; 2018. [Available from: https://www.ema.europa.eu/en/medicines/human/EPAR/mepsevii.]

112. Harmatz P, Whitley CB, Wang RY, Bauer M, Song W, Haller C, et al. A novel Blind Start study design to investigate vestronidase alfa for mucopolysaccharidosis VII, an ultra-rare genetic disease. Mol Genet Metab. 2018;123(4):488-94.

113. Wang RY, da Silva Franco JF, López-Valdez J, Martins E, Sutton VR, Whitley CB, et al. The long-term safety and efficacy of vestronidase alfa, rhGUS enzyme replacement therapy, in subjects with mucopolysaccharidosis VII. Mol Genet Metab. 2020;129(3):219-27.

114. Gonzalez-Meneses Lopez AGL, Beuno MB, Lau HL, Viskochil DV, Tanpaiboon PT, Martins EM, et al. P-371 Vestronidase alfa stabilizes or improves disease manifestations in subjects with MPS VII. J Inherit Metab Dis. 2018;41:S187-S8.

115. Lau HA, Viskochil D, Tanpaiboon P, Gonzalez-Meneses Lopez A, Martins E, Taylor J, et al. Long-term efficacy and safety of vestronidase alfa enzyme replacement therapy in pediatric subjects with mucopolysaccharidosis VII < 5 years old. Mol Gen Metab. 2020;129:S95.

1. A number of secondary outcomes of seizure frequency/duration have not been included as they show consistent findings with the primary outcome. [↑](#footnote-ref-1)
