# Supplementary material for: Exploring the feasibility of using the ICER Evidence Rating Matrix for Comparative Clinical Effectiveness in assessing treatment benefit and certainty in the clinical evidence on orphan therapies for paediatric indications
Source: Orphanet J Rare Dis. 2023 Jul 20;18:193. doi: 10.1186/s13023-023-02701-w (PMC10360248; doi:10.1186/s13023-023-02701-w)
Supplement: Supplementary file 4 — Additional file 4: Appendix 4. Narrative summaries of the evidence. [file 13023_2023_2701_MOESM4_ESM.docx]

**Appendix 4 – narrative summaries of the evidence for each of the 11 treatments with 16 indications**

Each indication was graded and rated by four reviewers.

**Nusinersen for Type I SMA**

All reviewers rated the net health benefit (NHB) of nusinersen to treat Type I (infantile onset) spinal muscular atrophy (SMA) as substantial, based on evidence demonstrating survival and function benefit. Evidence for the therapy was available from one RCT and 14 single arm trial extensions or observational studies involving patients receiving nusinersen. 312 patients were in seven trials solely of Type 1 patients, three studies (n=51) included patients with either Type I or Type II SMA, and four trials (n=503) included patients with Type I, Type II, or Type III SMA. The certainty of evidence was judged high by 2 reviewers and high-to-moderate by 2 reviewers. The overall evidence rating for nusinersen for Type I SMA was A.

The clinical benefit of nusinersen was established versus a sham control since no alternative treatment was identified. Response, measured by the Hammersmith Infant Neurological Examination (HINE) in the ENDEAR trial (N=122), found that motor function was achieved in 51% patients receiving nusinersen versus 0% in the sham control group over 187-280 days [1, 2]. A HINE improvement of 0.4 to 0.7 points is considered clinically meaningful. 28.6% of the 21 patients in a Brazilian before-and-after study achieved some motor milestones or gained at least 3 points on the HINE-2 at 24 months [3].

For event free survival (defined as time to death or permanent ventilation), the hazard ratio (HR) reported in the RCT was 0.53 (EFS of 61% for nusinersen vs. 32% for sham control). The overall survival (OS) HR in the RCT was 0.37 (84% for nusinersen vs. 61% for sham control) with comparative survival data available for up to 56 weeks. There was insufficient evidence to evaluate the treatment’s curative potential.

In the SHINE extension study to the ENDEAR RCT, patients treated for up to 280 days with sham treatment and then with nusinersen during the extension study had a decrease in the CHOP-INTEND score from 29.6 to 24.5 at 480 days [4]. A CHOP-INTEND improvement of 3.4 to 4.0 points is considered clinically meaningful [5]. Grade 3 and 4 adverse effects (>30%) were frequent, but less frequent among patients receiving sham treatment [2]. The CHOP-INTEND score for nusinersen was also measured in a series of national before-and-after studies. The score improved across studies by 16.8 (from 27.84 to 44.6) after 120 days of active treatment [4], by 9.8 after 6 months, by 6.7 after 12 months [6], by 14.9 after 307 days [7], by 14.8 after 12 months [8], by 7 (median) after 22 months [9], by 14 after 24 months [3], and by 8.7 after 24 months [6]. One small Japanese cohort study reported no improvement over 6-9 months [10].

**Nusinersen, SMA Type II/III**

The NHB of nusinersen for the treatment for Type II/III SMA was graded as substantial by 2 reviewers and small by 2 reviewers based on evidence demonstrating improved function and health related quality of life (HRQoL). Evidence considered in the rating was derived from two RCTs (CHERISH and EMBRACE) and 11 single arm trial extensions or observational studies. 316 patients were in seven trials solely of Type II/III patients, three studies (n=51) included patients with either Type I or Type II SMA, and four trials (n=503) included patients with Type I, Type II, or Type III SMA. The certainty of evidence was judged high-to-moderate by 2 reviewers and moderate by 2 reviewers. The overall rating of nusinersen for Type II/III SMA was B+.

Clinical benefit was established versus sham controls in the two RCTs since no alternative treatment was identified. Improvement in motor function was measured on the Hammersmith Functional Motor Scale Expanded (HFMSE) and was 4.9 points greater for nusinersen treatment (3.9) than for the sham control (-1.0) in the CHERISH trial, where deterioration was observed over 15 months [11]. An HFMSE improvement of 2-4 points is considered clinically meaningful, although patients and caregivers consider a 1-point increase meaningful [12, 13]. In CHERISH, 57% of nusinersen patients compared to 26% receiving the sham treatment achieved at least a 3-point improvement in HFMSE motor function [11, 14]. The improvement was maintained during the SHINE extension study with nusinersen patients continuing with the treatment achieving HFMSE score of 26.1 at 480 days, compared to a score of 21.2 for patients receiving the sham control in CHERISH and nusinersen in the extension [15]. In a Brazilian before-and-after study, nusinersen improved HFMSE score by a mean of 1.47 points after 12 months and by a mean of 1.60 points after 24 months of treatment, while in the control group the changes were -1.71 points (12 months) and -3.93 points (24 months), demonstrating clinically meaningful differences of 3.18 points (12 months) and 5.53 (24 months) [16].

For Type II SMA alone, one registry study and two before-and-after studies reported improvements of 3.75 points for HFMSE with nusinersen from baseline (6 months), 1.9 points (12 months) and an annual increase of 3.25 points/year (based on 4.8 months of treatment) [8, 17, 18]. In a single arm cohort of patients with Type II SMA, the HFSME improvement was 7.2 points after 307 days of nusinersen treatment [7].

For Type III SMA alone, one registry study reported an increase for HFSME of 5.4 points/year after 7.5 months of follow-up [8]. In non-comparative studies, the magnitude of the mean treatment effect was judged to be uncertain, and it was assumed that without nusinersen, an HFSME improvement greater than 2 is highly unlikely, as comparative studies demonstrated deterioration without active treatment.

Outcomes were also measured using change in the Revised Upper Limb Module (RULM) in the CHERISH trial. The RULM improvement was 3.7 points greater for nusinersen patients than for those receiving the sham control (4.2 versus 0.5) over 15 months [11]. A 2-point RULM improvement was considered clinically meaningful [19]. The improvement was maintained during the SHINE extension study, suggesting that longer treatment with nusinersen does not change the magnitude of the effect: the RULM score was 25.4 at 480 days among patients who received nusinersen in the trial and the extension, compared to 22.7 for patients who had received sham treatment prior to nusinersen in the extension study [15]. For Type II SMA alone, one registry study and two before-and-after studies reported that nusinersen treatment improved the RULM score by 2 points per year (at 4.8 months of follow-up), 4.3 points (at 307 days), and by 1.59 points (at 12 months) [8] [7, 18]. In these non-comparative studies, the magnitude of the mean treatment effect was judged to be uncertain, and it was assumed that without nusinersen any improvement greater than 0.5 points is highly unlikely based on comparative evidence.

The CHOP-INTEND score increased with nusinersen treatment by 4 points at 6 months in one before-and-after study [17] and, in a second before and after study, by 2.37 points at 12 months and by 3.42 at 24 months [16]. Clinically meaningful differences for CHOP-INTEND in Type II/III SMA have not been reported, and the change was judged to be uncertain, but considering the baseline score of 32.27 and standard deviation (SD) of 5.78, it was noted that the improvement was less than 1 SD of baseline score [16].

The change in the six-minute walk test (6MWT) was reported for Type III SMA only in a before-and-after study. Nusinersen patients experienced an increase of 33.9 meters at day 307 [7]. A clinically meaningful difference for 6MWT had been estimated as 30 minutes [20] or to range from 48 to 72 meters in later-onset SMA [21].

Data on the effects of nusinersen on forced vital capacity (FVC) were identified from three studies. A German before-and-after study found no significant change from baseline in FVC at day 300 [22]. A French before-and-after study found significant differences in favour of nusinersen compared to historical controls for FVC % predicted for both Type II SMA (p=0.029) and Type III SMA (p=0.018)[23]. An Australian before-and-after study reported that nusinersen stabilised FVC with the z-score changing from -0.5 per annum to +0.002 for Type II SMA [24, 25]. The change was not statistically significant (from -0.4 to -0.001) for Type III SMA [24, 25].

HR-QoL was reported using two scales in the CHERISH RCT: the Assessment of Caregiver Experience with Neuromuscular Disease (ACEND) and the Pediatric Quality of Life Inventory (PedsQL). The impact on caregivers measured by ACEND was reduced at months 6 and 15 months for 3 of 7 domains (feeding/grooming/dressing, transfer, and mobility) in the nusinersen group, while the impact on caregivers of patients receiving sham treatment was increased in these domains at both assessment points [26]. In the SHINE extension study, the change in ACEND was evaluated at day 1170 with mean changes from baseline in feeding/grooming/dressing (10.3), sitting/playing (1.1), transfers (3.5), mobility (5.0), and finance (0.7) [27]. A clinically meaningful difference for ACEND has not been established. For PedsQL, the mean difference from baseline for nusinersen vs. sham in CHERISH was 5.0 for the parent assessment at 15 months [26]. In the SHINE extension study, the PedsQL generic total score mean change from baseline to day 1170 was 2.3 for patients who received nusinersen in the trial and the extension, and the PedsQL NM score change was ‐0.2 [27]. A change of 4.5 is considered clinically meaningful for the PedsQL generic score, but the value for PedsQL NM has not been established [28].

**Nusinersen, Presymptomatic SMA**

The NHB of nusinersen in the treatment for Type II/III SMA was graded as comparable by 2 reviewers and small by 2 reviewers based on evidence from one single arm open label trial (NURTURE) of 25 patients. The certainty of evidence was judged low to moderate by two reviewers, moderate by one reviewer and high to moderate by one reviewer. The overall rating of nusinersen in presymptomatic SMA was C+.

NURTURE demonstrated benefits in terms of survival, time to respiratory intervention, achievement of developmental milestones and improvement in motor function. After a median 34 months of follow-up, all children were alive, and none required permanent ventilation [29]. The study had no comparator group, but natural history data was considered relevant evidence. Individuals with Type I SMA (the expected phenotype for approximately half of the enrolled cohort based on SMN2 copy number) die or require permanent ventilation at a median age of 13.5 months [30]. In the NURTURE trial, all the infants achieved the World Health Organization (WHO) milestone of sitting without support, 92% of those with two SMN2 copies achieved walking with assistance (100% with three SMN2 copies), and 88% were walking independently (100% with three SMN2 copies) [30]. HINE-2 total scores increased from 2.7 to 23.9 by day 778 for infants with two SMN2 copies and from 3.2 to 26.0 for those with three SMN2 copies [30]. A score of more than 1 point for any given HINE-2 milestone is highly unlikely in untreated patients with SMA type I [31] which suggests the NURTURE results are likely to be clinically meaningful.

The CHOP-INTEND total score changed during the same period from 47.0 to 62.1 for infants with two SMN2 copies and from 51.9 to 63.4 for those with three SMN2 copies. Comparative data was not collected in NURTURE, but natural history results were identified. These were not consistent with the NURTURE outcomes [32] since the CHOP-INTEND scores from NURTURE exceeded those observed in a natural history cohort of symptomatic infants with SMA, who experienced a mean decline of 10.71 points over 6 to 12 months [33].

**Dinutuximab beta for high-risk neuroblastoma, maintenance**

The NHB of dinutuximab beta for the maintenance treatment of high-risk neuroblastoma was graded as substantial by all four reviewers based on evidence from one RCT with three randomisations and one non-contemporaneous (historical) arm with N=1,280 patients studied in total. All four reviewers judged the certainty of evidence to be high. The overall rating of dinutuximab beta for high-risk neuroblastoma maintenance treatment was A.

The study showed event free survival benefit. Five-year EFS with dinutuximab beta and isotretinoin was 57% compared to 42% with isotretinoin alone, representing a 35.7% relative improvement. The 5-year EFS HR for patients treated with dinutuximab beta was 1.75 (reciprocal of 0.57) and 1.5 (0.67 as reciprocal) in two different analyses [34] [35]. At 3 years, EFS was 60% for dinutuximab beta with IL-2 and 56% for dinutuximab beta without IL-2 for patients treated with short-term infusion and 56% and 64% for long-term infusion, respectively, with no differences between the two randomisation groups [36, 37]. Overall survival at 5 years was 64% for dinutuximab beta plus isotretinoin compared to 50% for isotretinoin alone (HR=0.70) [38]. The HR was reported to be 0.63 (reciprocal of 1.60) in a second analysis [35]. According to ESMO criteria, a greater than 5% survival benefit at ≥ 3 years follow-up is of highest magnitude (Grade A). According to ASCO recommendation, 20% or more relative improvement in overall survival (i.e., HR ≤ 0.8) is considered a clinically meaningful benefit [39, 40].

**Dinutuximab beta for relapsed/refractory high-risk neuroblastoma**

The NHB of dinutuximab beta for relapsed/refractory high-risk neuroblastoma was graded as substantial by 1 reviewer and small by 3 reviewers based on evidence of survival benefit from two single-arm studies [41, 42], one with historical controls (N=128), and a pooled analysis of trial data. The certainty of evidence was judged moderate by 1 reviewer, high-to-moderate by 2 reviewers and high by 1 reviewer. The overall rating of dinutuximab beta for the treatment of relapsed/refractory high-risk neuroblastoma was B+.

At 2 years, the OS was 69% for patients receiving dinutuximab beta with IL-2 and isotretinoin compared to 30% in the historical control without immunotherapy [43]. In a pooled analysis of clinical trial data, 3-year OS was 50% for patients receiving dinutuximab beta compared to 24% and 28% in two historical control groups [44]. The OS HRs for patients receiving dinutuximab beta were 0.43 and 0.56 versus two different historical control groups [38, 44]. According to ASCO recommendations, 20% or more relative improvement in overall survival (HR≤0.8) is considered a clinically meaningful benefit [40].

**Tisagenlecleucel for relapsed/refractory B-cell acute lymphoblastic leukaemia**

The NHB of tisagenlecleucel for the treatment of relapsed/refractory B-cell acute lymphoblastic leukaemia (ALL) was rated as substantial by 3 reviewers and small by 1 reviewer based on evidence from five single arm studies (N=550 patients enrolled), 2 retrospective studies (N=266 patients) and 1 extension study (N=73 patients). The studies showed benefits for disease remission, survival and HR-QoL. The certainty of evidence was judged moderate by 1 reviewer and high-to-moderate by 3 reviewers. The overall rating of tisagenlecleucel for the treatment of relapsed/refractory B-cell ALL was B+.

Overall remission, defined as complete remission (CR) or complete remission with incomplete count recovery (CRi), was reported in 6 studies at various timepoints after infusion as 95% (at 1 month or 28 days), 81% (at 3 months, lasting for at least 28 days), 85%(at 3 months), 95% (at 90 days), 91% (at 6 months), and 69% (lasting for at least 28 days during 6 months after infusion) [45, 46] [47] [42] [41] [48]. CR at 1 month was reported as 95% [49]. The magnitude of a clinically meaningful remission rate was not established, but the reported overall remission rates were considered by study authors to be clinically meaningful. In this assessment of comparative NHB it was assumed that without tisagenlecleucel, the remission rate of the disease would be unlikely to exceed 20%, based on evidence from historical treatments [42].

EFS was variously reported to be between 55% and 74% at 6 months (3 studies), between 51% and 68.2% (3 studies) at 12 months, and 58% at 18 months [45-47, 49, 50]. The EFS for a potential comparator was not identified for a population that included patients with multiple relapses. We judged comparative NHB based on an analysis adjusted for baseline characteristics finding that tisagenlecleucel prolonged OS compared with blinatumomab (HR=0.32), clofarabine monotherapy (HR=0.24), clofarabine combination regimens (HR=0.26), and two salvage therapies (HR=0.15 and 0.27) [51]. According to ASCO recommendations, a 20% or greater relative improvement in OS (HR≤0.8) is considered a clinically meaningful benefit [40].

HR-QoL was assessed using the PedsQL instrument and the EQ-5D questionnaire. For patients who responded to treatment, the mean change from baseline in the PedsQL total score was 13.5 at 3 months, 16.9 at six months and 27.2 at 12 months [47]. The mean change from baseline in the EQ-5D VAS score was 16.5 (3 months), 15.9 (six months) and 24.7 (12 months). This was considered a clinically meaningful improvement.

**Cannabidiol for Dravet syndrome**

The NHB of cannabidiol to treat Dravet syndrome was graded as substantial by 3 reviewers and small by 1 reviewer based on evidence on seizure frequency, caregiver assessment and HR-QoL benefit. Evidence considered in the rating was available from 2 RCTs (N=318 patients), 1 trial extension (N=315), 1 expanded access programme, 2 retrospective reviews of before-and-after data (N=15 patients) and a meta-analysis of the RCT data. The certainty of evidence was judged high-to-moderate by 2 reviewers and moderate by 2 reviewers. The overall rating of cannabidiol for the treatment of Dravet syndrome was B+.

In one RCT, cannabidiol reduced adjusted convulsive seizure frequency from baseline by 22.8% over 14 weeks with 43% of cannabidiol patients achieving at least a 50% reduction in convulsive seizure frequency compared with 27% of patients who received placebo [52]. In the second RCT, the reduction in convulsive seizure frequency versus placebo was 29.8% for the CBD20 dose group and 25.7% for the CBD10 dose group [53]. A reduction of at least 50% in convulsive seizures was reported for 49.3% of patients who received CBD20, 43.9% of patients who received CBD10 and 26.2% of patients who received a placebo [53]. In the extension study, the change from baseline in 12-week periods over 156 weeks ranged from 45% to 73% for convulsive seizures and from 49 to 80% for total seizures [54]. After a mean of 9.4 months of treatment in a UK retrospective review of before-and-after data (N=5), 20% of children with Dravet syndrome had a reduction in seizure frequency of more than 75%, 40% had reductions of between 30% to 75%, and 40% showed no improvement [55]. In a Korean retrospective review of before-and-after data for 10 patients, at 3 months, 10% of patients were seizure-free, 20% had a reduction in seizure frequency of more than 50%, and 60% had no change [56]. At 6 months, none of the patients were seizure-free and 20% had a reduction of more than 50%. A 50% reduction in seizure frequency is conventionally considered to be the cut-off for a clinically meaningful change, although a threshold of 44% has also been proposed [57] [58].

In one RCT, there was a -1.0 median difference in change from baseline in favour of cannabidiol versus placebo in the Caregiver Global Impression of Change (CGI-C) scale. On the CGI-C scale “much improved or better” change is considered clinically meaningful [57]. In the second RCT, the change in CGI-C in favour of cannabidiol was reported as odds ratios of 2.02 (CBD20) and 2.93 (CBD10) compared to placebo [53]. The absolute change in the cannabidiol and placebo arms was not reported and therefore its clinical meaningfulness could not be judged. It was, however, reported that patients were more likely to be “much improved” or “very much improved” in the cannabidiol arms of this trial compared to placebo: 36% (CBD20), 32% (CBD10) and 14% (placebo) [53].

In one RCT, the change from baseline for CBD20 versus placebo was reported to be -0.4 on the sleep disruption score (NRS scale), 1.5 on the Epworth Sleepiness Scale, 1.5 for the Quality of Life in Childhood Epilepsy score and -2.6 in the Vineland-II score [52]. In a second trial, the changes from baseline versus placebo were 0.0 (CBD10) and -0.1 (CBD20) for sleep disruption, -0.55 (CBD10) and 0.74 (CBD20) for the Epworth Sleepiness Scale, 3.8 (CBD10) and 1.8 (CBD20) for the Quality of Life in Childhood Epilepsy score and -0.4 (CBD10) and 0.0 (CBD20) for Vineland-II score [53]. None of the HR-QoL changes were statistically significant, but trends were still considered in the assessment of magnitude, and lack of significance was factored into the certainty assessment.

**Cannabidiol for Lennox-Gastaut syndrome**

The NHB of cannabidiol in the treatment of Lennox-Gastaut syndrome was graded as substantial by 3 reviewers and small by 1 reviewer based on evidence on seizure frequency, caregiver assessment and HR-QoL benefit. Evidence considered in the rating was available from 2 RCTs (N=396 patients), 1 single-arm trial extension (N=171), an expanded access programme study, 2 retrospective reviews of before and after data (N=43), a post-hoc analysis of the RCT data and a meta-analysis of the RCT data. Certainty of evidence was judged high-to-moderate by 2 reviewers and moderate by 2 reviewers. The overall rating of cannabidiol for the treatment of Lennox-Gastaut syndrome was B+.

In one RCT, the median difference in the reduction in drop seizures during 28-day periods was 21.6% for CBD20 vs. placebo, and 19.2% for CBD10 vs. placebo over 14 weeks of treatment [59]. In the second RCT, the median difference for CBD20 versus placebo was -17.21% over monthly periods during 14 weeks of treatment [60]. In the extension study (including both DS and LGS patients), the change from baseline in 12-week windows over 156 weeks ranged from 48% to 71% for convulsive seizures and from 48% to 68% for total seizures [61]. In a retrospective analysis of a before and after study of 9 patients, after a mean 9.4 months of treatment, a reduction of more than 75% of seizure frequency was seen in 33.33% of patients, between 30% to 75% reduction in 44.4% of patients and no improvement in 22.2% of patients [55]. In a Korean retrospective analysis of a before-and-after data for 34 patients, at 3 months, 23.5% of patients receiving cannabidiol were seizure-free, 8.8% had a reduction in seizure frequency of more than 50%, and 20.6% showed a reduction in seizure frequency of less than 50% [56]. At 6 months, 11.8% of patients were seizure-free, 8.8% had a reduction of more than 50%, and 8.8% showed a reduction of less than 50% [56]. In one RCT, 39% patients in the CBD20 group, 36% in the CBD10 group, and 14% in the placebo group had at least a 50% reduction from baseline in drop-seizure frequency during the treatment period [59]. In the second RCT, 44% of patients using CBD20 had a reduction in drop seizure frequency of 50% or more compared with 24% in the placebo group [60]. A 50% reduction in seizure frequency is conventionally considered to be the cut-off for a clinically meaningful change [58].

Reported as an odds ratio, the change in CGI-C in favour of cannabidiol was 1.83 (CBD20) and 2.57 (CBD10) in one RCT and 2.54 (CBD20) in a second RCT [59] [60]. The absolute change in the cannabidiol and placebo arms was not reported and therefore its clinical meaningfulness could not be judged. On the CGI-C scale a “much improved or better” change is considered clinically meaningful. In one RCT, patients were more likely to be “much improved” or “very much improved” in the cannabidiol arms (28% for CBD20 and 31.5% for CBD10) compared to the placebo arm (12%) [59]. In a second RCT, 35% of patients receiving CBD20 and 17% of patients receiving placebo were “very much improved” [60]. The changes from baseline versus placebo on HR-QoL measures were for CBD10 and CBD20 arms, respectively: Sleep disruption: -0.8 and –0.3; Epworth Sleepiness Scale: 0.09 and 0.01; Quality of Life in Childhood Epilepsy score: 1.6 and -5.1; Vineland-II score: 0.5 and 0.1 [59]. None of the HR-QoL changes were statistically significant, but trends were still considered in the assessment of magnitude, and lack of significance was factored into the certainty assessment.

**Metreleptin, congenital generalised lipodystrophy (LD) (Berardinelli-Seip syndrome) or acquired generalised LD (Lawrence syndrome)**

The NHB of metreleptin in congenital or acquired generalised LD was graded as small by 2 reviewers and comparable by 2 reviewers based on evidence of improved control of glycaemia and serum triglycerides (TG). Evidence considered in the rating was available from 1 single-arm trial with a long-term extension (N=66 patients) and 2 observational studies (N=37). Certainty of evidence was judged as high-to-moderate by 1 reviewer and moderate by 3 reviewers. The overall rating of metreleptin in the generalised LD population was C+.

The mean change from baseline in HbA1c in the three studies was -1.2% and -1.9% over 12 months in the observational studies and -2.2% over 12 months in the larger single arm trial [62] [63] [64]. The mean change from baseline in fasting TGs in the same three studies over 12 months was -26.9%, and -53% (observational studies) and -32.1% (single arm trial) [62-64]. In the single arm trial, 80% of patients experienced ≥1% decrease in HbA1c or ≥30% decrease in fasting serum TG, while 66% had ≥2% decrease in HbA1c or ≥40% decrease in fasting serum TG [62]. In one observational study, TG ≤200 mg/dL was achieved by 50% of patients (compared to 5% at baseline) and HbA1c ≤7% was achieved by 57% (compared to 22% at baseline) [63]. A minimum clinical response was defined by the European Medicines Agency (EMA) and is listed in the Summary of Product Characteristics (SmPC) as at least a 0.5% HbA1c reduction and/or 15% reduction in triglycerides (TGs) [62]. Comparator data have not been identified, and the magnitude of treatment effect was judged based on the natural history of disease, whereby the severity of disease in inadequately treated generalised LD patients worsens over time [62].

**Metreleptin, acquired partial LD (Barraquer-Simons syndrome)**

The NHB of metreleptin in acquired partial LD was graded as small by 1 reviewer and comparable by 3 reviewers based on evidence of improved control of glycaemia and serum TGs. Evidence considered in the rating was available from 1 single-arm trial with long-term extension (N=41) and 2 observational studies with 57 patients. Certainty of evidence was judged low-to-moderate by 1 reviewer and low by 3 reviewers. The overall rating of metreleptin for patients with acquired partial LD was I (insufficient).

The mean change from baseline in HbA1c over twelve months was -0.4% and -0.5% in the small observational studies and -0.6% in the single arm trial [62] [63] [64]. The mean change in fasting TGs over twelve months was -8.7% and -21.5% in the small observational studies and -20.8% in the single arm trial [62] [63] [64]. 68% of patients in the single arm trial experienced ≥1% decrease in HbA1c or ≥30% decrease in fasting serum TG, while 43% had ≥2% decrease in HbA1c or ≥40% decrease in fasting serum TG [62]. In one observational study, TG ≤200 mg/dL was achieved by 52% of patients (compared to 29% at baseline) and HbA1c ≤7% was unchanged from the 32% at baseline [63]. A minimum clinical response was defined by the European Medicines Agency (EMA) and described in the SmPC as at least a 0.5% HbA1c reduction and/or 15% reduction in TGs [62]. Comparator data have not been identified, and the magnitude of treatment effect was judged based on the natural history of disease, whereby the severity of disease in inadequately treated acquired partial LD patients worsens over time [62].

**Burosumab in X-linked hypophosphatemia**

The NHB of burosumab in X-linked hypophosphatemia was graded as small by 3 reviewers and comparable by 1 reviewer based on evidence for improvements in rickets severity, growth, motor function and HR-QoL. Evidence considered in the rating was available from 2 RCTs (N=113), one single-arm trial (N=13) and 1 observational study (N=7). Certainty of evidence was judged high-to-moderate by 2 reviewers and moderate by 2 reviewers. The overall rating of burosumab for the treatment of X-linked hypophosphatemia was B+.

In one RCT, the mean Radiographic Global Impression of Change (RGI-C) score, measuring severity of rickets, at week 64 following treatment with burosumab was greater by 2.1 compared to treatment with phosphate >40 mg/kg (1.0), phosphate <=40 mg/kg (1.0), alfacalcidol >60 ng/kg or calcitriol >30 ng/kg (1.5), or alfacalcidol >60 ng/kg or calcitriol >30 ng/kg (0.7) [65] [66] [67]. In a single arm study, the RGI-C score improved by 2.2 over 64 and 160 months [68]. For the RGI-C, scores of +1 indicate minimal healing, +2 indicates substantial healing and +3 indicates complete/near complete healing [69].

In one RCT, the improvement in lower limb deformity with burosumab (+1.3) was greater than for the comparators (variously +0.3, +0.3, +0.4, +0.2) [66] [67].

Changes in the Rickets Severity Score (RSS) reported in various single arm studies of burosumab were -1.1 (for burosumab twice weekly measured at week 40), -1.86 (12 months), -2.0 (week 64) and -1.9 (week 160) (some figures calculated by the authors) [70] [71] [68, 69, 72, 73]. RSS scores ≥1.5 are associated with more severe clinical features and changes of this magnitude was considered clinically meaningful [69].

Fasting serum phosphorus concentration increased by 0.29 mmol/L (weeks 40 and 64) for patients receiving burosumab in one RCT and by 0.06 mmol/L (week 40) and 0.07mmol/L (week 64) for those receiving conventional therapy [65]. In a single arm study, serum phosphorus increased for patients receiving burosumab by 0.9 mmol/L (at 64 weeks and 160 weeks) (some figures calculated by the authors) [73]. Changes of <0.5 mg/dL (0.16 mmol/L) were used as a criterion for dose adjustment in the trial, hence we considered it a clinically meaningful change [70]. In one RCT, burosumab was associated with improvement in length/height, with a 0.14 greater increase in Z-score at week 64 than conventional therapy and in a small before and after study length/height increased by 7 cm (5.8%) over twelve months from baseline [65, 72]. In the RCT, patients receiving burosumab also demonstrated 7% greater improvement in 6MWT than the conventional therapy, but no values to assess clinical meaningfulness were identified [65].

In one RCT, the PROMIS Pain Interference score improved by -5.02 (week 40) and -2.26 (week 64) compared to conventional therapy [66] [67]. The improvement in patients receiving burosumab, as well as differences in improvement versus conventional therapy met the criterion for minimal important difference of 2.5 [74]. The improvement in Physical Function Mobility scores was +2.68 (week 40) and +1.90 (week 64), and the improvement in Fatigue scores was -3.25 (week 40) and -1.08 (week 64), for which the criterion for difference versus the comparator was not met.

The improvement with burosumab compared to conventional therapy using the SF-10 Health Survey for Children (physical health scores) was +4.33 (week 40) and +5.49 (week 64), although the differences were not statistically significant and were not assessed for clinical meaningfulness.

**Vestronidase alfa in mucopolysaccharidosis VII**

The NHB of vestronidase alfa for the treatment of mucopolysaccharidosis VII was rated as small by 1 reviewer and comparable by 3 reviewers based on evidence on urinary glycosaminoglycans (uGAG) excretion, clinical morbidities, motor function and HR-QoL. Evidence considered in the rating was available from 1 crossover placebo-controlled trial (N=12) with a single-arm trial extension (N=12) and 1 single arm trial (N=8). Certainty of evidence was judged to be low by all four reviewers. The overall rating of vestronidase alfa for the treatment of mucopolysaccharidosis VII was I (insufficient).

The cross-over trial found a mean reduction from baseline in uGAG dermatan sulfate excretion with vestronidase alfa treatment at 24 weeks, with uGAG dermatan sulfate levels reduced by 64.8% and a reduction in chondroitin sulfate of 70.6 % [75]. All patients in the trial were responders, achieving ≥ 50% reduction from baseline in uGAG [76]. At 48 weeks in the extension study the reduction in uGAG following treatment with vestronidase alfa was -58%, and in the single arm study was 61% [77, 78]. The change in uGAGs was rapid, consistent, and sustained. Even though a clinical meaningfulness threshold was not established, it was understood that this change had meaningful clinical implications based on critical importance of the metabolite in disease pathogenesis and for the mechanism of action. The Multi-Domain Responder Index (MDRI) reported in the cross-over trial showed a mean change of 0.5 at week 24, with 83.3% of patients achieving clinically meaningful improvement (of at least 1) in at least one of the 6 MDRI clinical domains (6MWT, FVCl, shoulder flexion, visual acuity, and Bruininks-Oseretsky Test of Motor Proficiency (BOT-2)) [75]. At week 48, the mean MDRI change was 0.9 in the extension study [78]. We assumed that without replacement therapy with vestronidase alfa any improvement is unlikely. 75% of patients improved over baseline in fatigue at some point during the study [75]. At week 24, the mean change from baseline in the Fatigue Total Score (PedsQL) was 3.4. A change of 10 points is considered a minimal important difference [76]. In the small phase II study, mean growth velocity increased from 5.06 cm/year in the 2 years before treatment to 6.84 cm/year following treatment, but comparative natural history data showing growth velocity in untreated patients were not identified [79].

**Velmanase alfa for alpha-mannosidosis**

The NHB of velmanase alfa for the treatment of alpha-mannosidosis was graded as small by 1 reviewer and comparable by 3 reviewers based on evidence relating to serum oligosaccharides, clinical function, motor function and HR-QoL. Evidence considered in the rating was available from 1 RCT (N=25) and 1 integrated observational data analysis (N=33). Certainty of evidence was judged low by 2 reviewers, low-to-moderate by 1 reviewer and moderate by 1 reviewer. The overall rating of velmanase alfa for the treatment of alpha-mannosidosis was C+.

In the RCT, the mean change in serum oligosaccharides from baseline to 52 weeks for patients treated with velmanase alfa compared to placebo was -70.5% (-3.50 μmol/L) with 4 μmol/L considered a minimal clinically important difference [80, 81]. In the single arm extension study to the RCT, the change in the velmanase alfa arm was -77.6% at 52 weeks and -62.76% at 48 months [80] [81]. The 3-min stair climb test (3MSCT) changed by -1.1% for patients receiving velmanase alfa (3% more than for patients receiving the placebo) at 52 weeks in the RCT and by 13.77% (6.38 steps/min) in the single arm extension at 48 months with ≥7 steps/min considered a minimal clinically important difference [80, 81]. Patients receiving velmanase alfa experienced a 3.7m change in 6MWT (7.3m or 1.9% more compared to patients receiving the placebo) at 52 weeks in the RCT, and a 69.7% change from baseline at 48 months, with ≥30m considered to represent a minimal clinically important difference [80] [81]. In the RCT, a change of 8.2 in FVC % predicted was reported for patients receiving velmanase alfa (8.4% more than for those receiving placebo) at 52 weeks and in the extension study a mean absolute change of 8.1 was reported at 48 months [80] [81]. For FVC, an absolute change of ≥30% is considered a minimal clinically important difference [81].

Various outcomes for quality of life are reported from a single arm study of 33 people treated with velmanase alfa. The Childhood Health Assessment Questionnaire Disability Index (CHAQ-DI) changed from baseline to 12 months by -0.10 and from baseline to last observation by -0.13 [81]. A change of 13 or more is considered a minimal clinically important difference in CHAQ-DI [82] [83]. The CHAQ VAS Pain score changed by 0.148 at 12 months and by -0.173 at last observation [81, 83]. The corresponding mean percentage change was 3.697% and -17.0%. In the paediatric group, however, the change at 12 months was -13.7% and at last observation was -0.4%. The minimal clinically important improvement for the score was defined as ≥8.2% [84]. The EQ-5D-5L change from baseline was 0.0346 (6.932%) at 12 months and 0.050 (11.23%) at last observation [83]. Pediatric EQ-5D-5L health index values changed from baseline to 12 months by -0.513% (8.538%) and increased to last observation by 17.49% (28.27) [83]. The minimal clinically important difference for EQ-5D-5L was estimated to be 0.05 to 0.08 [85]. The change in EQ-5D-5L VAS for Best Health mean score at last observation was 3.3 [83]. Disease specific clinically meaningful values of change for the EQ-5D-5L VAS have not been identified.

**Glibenclamide oral suspension for neonatal diabetes mellitus**

The NHB of glibenclamide in the treatment of neonatal diabetes mellitus was graded as small by 2 reviewers and comparable by 2 reviewers based on evidence on withdrawal of insulin therapy and control of glycaemia. Evidence considered in the rating was available from 4 single-arm prospective studies (N=158). Certainty of evidence was judged moderate by 2 reviewers and high-to-moderate by 2 reviewers. The overall rating of glibenclamide oral suspension for neonatal diabetes mellitus was C+.

In three studies, 90% of patients successfully discontinued insulin after receiving glibenclamide (glyburide) over 12 months, 100% over 18 months and 93% over 10 years [86] [87] [88]. The mean reduction in HbA1c with glibenclamide was 1.7% by 12 weeks, 1.55% by 18 months, 2.2% over 1 year (calculated by the authors) and 1.7% at last follow-up over 10 years (calculated by the authors) [87] [88]. No comparative evidence has been identified, however, according to the EMA “although only uncontrolled data are available, the consistent effects on HbA1c across studies and the magnitude of the observed reduction are considered sufficient to conclude on a favourable metabolic effect in the short-term” [89].

**Chenodeoxycholic acid for cerebrotendinous xanthomatosis**

The NHB of chenodeoxycholic acid (CDCA) in cerebrotendinous xanthomatosis (CTX) was graded as comparable by all four reviewers based on evidence relating to levels of cholestanol and improvements in clinical symptoms. Evidence considered in the rating was available from 5 single-arm retrospective studies (N=183). Certainty of evidence was judged low by 3 reviewers and low-to-moderate by 1 reviewer. The overall rating of CDCA for the treatment of CTX was I (insufficient).

In one study, mean plasma cholestanol decreased by 26 mg/L (23.1%) to 6mg/L with CDCA therapy after a mean 8 years of follow-up [90]. The normal range of plasma cholestanol is considered <5.0 mg/L. Levels of cholestanol in the cerebrospinal fluid and plasma of untreated CTX patients were found to be about 20-fold higher than in patients treated with CDCA [91]. In another study, with 5-year follow-up, cholestanol levels decreased with CDCA treatment from 62 to 9 μmol/L within a few months and in two further studies cholestanol levels decreased significantly with CDCA over 10 years from 76.5 and 87.8 μmol/L to approximately 70 μmol/L, with 7-11 μmol/L considered normal range [92-94].

In one study, an improvement in clinical symptoms was observed in 57% of CDCA treated patients with deterioration in 20% over a mean 8 years of follow-up [90]. The Expanded Disability Status Scale (EDSS) in one study worsened in 3 (25%) patients, improved in 3 (25%) patients, and remained stable in 6 (50%) over 5 years, with the score improving from 2.5 to 1.9 [92]. The EDSS clinically important difference (established for multiple sclerosis) is 1.0 point when the score is between 0 and 5.5, and 0.5 when the score is between 5.5 and 8.5, as established for multiple sclerosis [95] [96]. In other studies, EDSS scores improved in 23%, stabilised in 54% and deteriorated in 23% (with a median 0.0 change from baseline) in The Netherlands, and improved in 4%, stabilised in 46% and deteriorated in 50% (median 0.5 change from baseline) in Italy [93]. Over a median of 8 years, in one study, EDSS scores and Modified Rankin Scale (mRS) scores improved in all patients aged under 24 years [97]. In two other studies, mRS scores improved in 15% of patients, stabilised in 69% patients and deteriorated in 15% over 9 years (median 0.0 change from baseline) in The Netherlands and remained unchanged in 62% of patients in Italy (median 0.0 change from baseline) [93]. A single point change in mRS can be considered clinically meaningful in stroke [98]. In one study, the Scale for the Assessment and Rating of Ataxia (ranging from 0 to 40) improved from 5.3 to 2.0, with scores between 1.1 to 1.5 considered a clinically meaningful change [92, 99].

**Cerliponase Alfa in CLN2 disease**

The NHB of cerliponase alfa in CLN2 disease was rated to be small by 2 reviewers and comparable by 2 reviewers based on evidence on improvements in motor and language function and HR-QoL. Evidence considered in the rating was available from 2 single-arm prospective studies and one study extension (N=37). Certainty of evidence was judged moderate by 2, high-to-moderate by 1 and low-to-moderate by 1 reviewer. The overall rating for cerliponase alfa in CLN2 disease was C+.

The time to 2-point decline in motor and language domains of the CLN2 Clinical Rating Scale in patients who received cerliponase alfa was not reached whereas historical controls reached 2-point decline in 345 days [100]. The HR for a 2-point decline in combined motor–language score versus a historical control was 0.08 [101]. The responder rate (no change or improvement in motor–language score) was 87% of treated patients at week 48 and 100% vs. 43% (historical control) at week 96 (difference 57%, p<0.0001). In the 190-203 study, the primary outcome was rate of decline in motor and language domains of the CLN2 Clinical Rating Scale [102]. The mean (SD) rate of decline was 0.14 points for treated children and 1.24 points for controls (mean difference: 1.10)[100]. The minimal clinically important difference is a decrease of one point [103]. In pre- versus post-treatment analysis, the motor-language score remained stable in 11 patients (treatment duration 11-43 months), improved in 1 patient by 1 point (in the motor domain) after 13 months of treatment, and declined in 2 patients by 1 point (in the motor domain) after 15 and 58 months of treatment.

Safety was assessed in terms of the frequency of adverse events of at least Grade 3 for all the indications. In no cases did it affect the NHB to impact the overall rating.

**References**

1. European Medicines Agency Committee for Medicinal Products for Human Use (CHMP). CHMP assessment report: Spinraza (EMEA/H/C/004312/0000). European Medicines Agency. 2017. <https://www.ema.europa.eu/en/medicines/human/EPAR/spinraza>. Accessed December 2020

2. Finkel RS, Mercuri E, Darras BT, Connolly AM, Kuntz NL, Kirschner J, et al. Nusinersen versus Sham Control in Infantile-Onset Spinal Muscular Atrophy. N Engl J Med. 2017;377(18):1723-32.

3. Mendonça RH, Jorge Polido G, Ciro M, Jorge Fontoura Solla D, Conti Reed U, Zanoteli E. Clinical Outcomes in Patients with Spinal Muscular Atrophy Type 1 Treated with Nusinersen. (2214-3602 (Electronic)).

4. Finkel R, Castro D, Farrar M, Tulinius M, Krosschell K, Saito K, et al. P.266 Nusinersen in infantile-onset spinal muscular atrophy: results from longer-term treatment from the open-label SHINE extension study. Neuromuscular Disorders. 2020;30(S124).

5. Stull D, Williams V, Houghton K, Williams N, Teynor M. Minimal clinically important differences in motor function in patients with infantile-onset spinal muscular atrophy: results from the Phase 3 ENDEAR trial. Poster presented at the 2019 AMCP Managed Care & Specialty Pharmacy Annual Meeting; March 25, 2019. San Diego, CA. [abstract] J Manag Care Spec Pharm. 2019;25:S55.

6. Pane M, Coratti G, Sansone VA, Messina S, Catteruccia M, Bruno C, et al. Type I SMA "new natural history": long-term data in nusinersen-treated patients. Ann Clin Transl Neurol. 2021;8(3):548-57.

7. Szabo L, Gergely A, Jakus R, Fogarasi A, Grosz Z, Molnar MJ, et al. Efficacy of nusinersen in type 1, 2 and 3 spinal muscular atrophy: Real world data from Hungarian patients. Eur J Paediatr Neurol. 2020;27:37-42.

8. Hodgkinson-Brechenmacher V, Oskoui M, Campbell C, Lounsberry J, Brais B, MacKenzie A, et al. P.174 The Canadian neuromuscular disease registry: A national spinal muscular atrophy (SMA) registry for real world evidence. Neuromuscular Disorders. 2020;30:S97-S8.

9. Edel L, Abbott L, Chan E, Main M, Robinson V, Munot P, et al. SMA - CLINICAL: P.92 Comparing motor and respiratory function in SMA Type1 treated with Nusinersen using CHOP INTEND(CHOP) &amp; Great Ormond Street Respiratory Score(GSR). Neuromuscular Disorders. 2020;30:S74.

10. Tokunaga S, Shimomura H, Taniguchi N, Lee T, Takeshima Y. Analysis of the therapeutic effect of nusinersen in patients with spinal muscular atrophy using a motor function scale and questionnaire. No To Hattatsu. 2020;52(6):390-6.

11. Mercuri E, Darras BT, Chiriboga CA, Day JW, Campbell C, Connolly AM, et al. Nusinersen versus Sham Control in Later-Onset Spinal Muscular Atrophy. N Engl J Med. 2018;378(7):625-35.

12. Williams V, Stull D, Houghton K, Williams N, Teynor M. Minimal clinically important differences of the expanded hammersmith functional motor scale in later-onset spinal muscular atrophy: results from the Phase 3 CHERISH trial. Poster presented at the 2019 AMCP Managed Care & Specialty Pharmacy Annual Meeting; March 25, 2019. San Diego, CA. [abstract] J Manag Care Spec Pharm. 2019;25:S54.

13. McGraw S, Qian Y, Henne J, Jarecki J, Hobby K, Yeh WS. A qualitative study of perceptions of meaningful change in spinal muscular atrophy. BMC Neurol. 2017;17(1):68.

14. Mercuri E, Finkel R, Montes J, Mazzone ES, Sormani MP, Main M, et al. Patterns of disease progression in type 2 and 3 SMA: Implications for clinical trials. Neuromuscul Disord. 2016;26(2):126-31.

15. Mercuri E, Darras B, Chiriboga C, Farrar M, Kirschner J, Kuntz N, et al. P.257 Longer-term treatment with nusinersen: Results in later-onset spinal muscular atrophy from the SHINE study. Neuromuscular Disorders. 2020;30:S121.

16. Mendonca RH, Polido GJ, Matsui C, Silva AMS, Solla DJF, Reed UC, et al. Real-World Data from Nusinersen Treatment for Patients with Later-Onset Spinal Muscular Atrophy: A Single Center Experience. J Neuromuscul Dis. 2021;8(1):101-8.

17. Ortenzi GB, Palmas G, Andresciani E, Garzone AMF, Berardi MA, Siliquini S, et al. Effectiveness of Nusinersen in paediatric patients SMA1 and SMA2. European Journal of Hospital Pharmacy. 2020;27:A109.

18. Coratti G, Pane M, Lucibello S, Pera MC, Pasternak A, Montes J, et al. Age related treatment effect in type II Spinal Muscular Atrophy pediatric patients treated with nusinersen. Neuromuscul Disord. 2021;31(7):596-602.

19. Pera MC, Coratti G, Mazzone ES, Montes J, Scoto M, De Sanctis R, et al. Revised upper limb module for spinal muscular atrophy: 12 month changes. Muscle Nerve. 2019;59(4):426-30.

20. Dunaway Young S, Montes J, Kramer SS, Marra J, Salazar R, Cruz R, et al. Six‐minute walk test is reliable and valid in spinal muscular atrophy. Muscle Nerve. 2016;54:826-42.

21. Stolte B, Bois JM, Bolz S, Kizina K, Totzeck A, Schlag M, et al. Minimal clinically important differences in functional motor scores in adults with spinal muscular atrophy. Eur J Neurol. 2020;27(12):2586-94.

22. Heitschmidt L, Pichlmaier L, Eckerland M, Steindor M, Olivier M, Fuge I, et al. Nusinersen does not improve lung function in a cohort of children with spinal muscular atrophy - A single-center retrospective study. Eur J Paediatr Neurol. 2021;31:88-91.

23. Gomez-Garcia de la Banda M, Amaddeo A, Khirani S, Pruvost S, Barnerias C, Dabaj I, et al. Assessment of respiratory muscles and motor function in children with SMA treated by nusinersen. Pediatr Pulmonol. 2021;56(1):299-306.

24. Chacko A, Deegan S, Gauld L, Sly P. Nusinersen stabilises respiratory function in paediatric spinal muscular atrophy. European Respiratory Journal. 2020;56:1234.

25. Chacko A, Deegan S, Sly P, Gauld L. Stabilization of respiratory function in pediatric spinal muscular atrophy treated with nusinersen. American Journal of Respiratory and Critical Care Medicine. 2020;201:A1161.

26. Johnson NB, Paradis AD, Naoshy S, Wong J, Montes J, Krasinski DC. Evaluation of nusinersen on impact of caregiver experience and hrqol in later-onset spinal muscular atrophy (SMA): Results from the phase 3 cherish trial. Neurology. 2020;94.

27. Montes J, Krasinski D, Foster R, Gambino G, Paradis A, Garafalo S, et al. P.269 Impact of Continued Nusinersin treatment on Caregiver Experience and Health-Related Quality of Life in Later-onset SMA: Results From the SHINE Study. Neuromuscular Disorders. 2020;30:S125.

28. Iannaccone ST, Hynan LS, Morton A, Buchanan R, Limbers CA, Varni JW, et al. The PedsQL in pediatric patients with Spinal Muscular Atrophy: feasibility, reliability, and validity of the Pediatric Quality of Life Inventory Generic Core Scales and Neuromuscular Module. Neuromuscul Disord. 2009;19(12):805-12.

29. De Vivo DC, Bertini E, Swoboda KJ, Hwu WL, Crawford TO, Finkel RS, et al. Nusinersen initiated in infants during the presymptomatic stage of spinal muscular atrophy: Interim efficacy and safety results from the Phase 2 NURTURE study. Neuromuscul Disord. 2019;29(11):842-56.

30. Finkel RS, McDermott MP, Kaufmann P, Darras BT, Chung WK, Sproule DM, et al. Observational study of spinal muscular atrophy type I and implications for clinical trials. Neurology. 2014;83(9):810-7.

31. De Sanctis R, Coratti G, Pasternak A, et al. Developmental milestones in type I spinal muscular atrophy. Neuromuscul Disord. 2016;26:754-9.

32. Finkel R, Bertini E, Muntoni F, Mercuri E. 209th ENMC International Workshop: Outcome Measures and Clinical Trial Readiness in Spinal Muscular Atrophy 7–9 November 2014, Heemskerk, the Netherlands. Neuromuscular Disorders. 2015;25(593-602).

33. Kolb SJ, Coffey CS, Yankey JW, Krosschell K, Arnold WD, Rutkove SB, et al. Natural history of infantile-onset spinal muscular atrophy. Ann Neurol. 2017;82:883-91.

34. Ladenstein R, Pötschger U, Valteau-Couanet D, Luksch R, Castel V, Ash S, et al. Investigation of the role of dinutuximab beta-based immunotherapy in the SIOPEN High-Risk Neuroblastoma 1 Trial (HR-NBL1). Cancers (Basel). 2020;12(2):309.

35. Holmes K, Potschger U, Pearson ADJ, Sarnacki S, Cecchetto G, Gomez-Chacon J, et al. Influence of Surgical Excision on the Survival of Patients With Stage 4 High-Risk Neuroblastoma: A Report From the HR-NBL1/SIOPEN Study. J Clin Oncol. 2020;38(25):2902-15.

36. Ladenstein RL, Poetschger U, Valteau-Couanet D, Gray J, Luksch R, Balwierz W, et al. Randomization of dose-reduced subcutaneous interleukin-2 (scIL2) in maintenance immunotherapy (IT) with anti-GD2 antibody dinutuximab beta (DB) long-term infusion (LTI) in front-line high-risk neuroblastoma patients: Early results from the HRNBL1/ SIOPEN trial. Journal of Clinical Oncology. 2019;37(Supplement 15).

37. Ladenstein RL, Poetschger U, Valteau-Couanet D, Gray J, Luksch R, Balwierz W, et al. Risk factors in the HR-NBL-1/SIOPEN study in patients receiving dinutuximab beta (DB) based immunotherapy. Journal of Clinical Oncology. 2020;38(15).

38. Wex J, Zibelnik N, Zemam A. Dinutuximab beta with isotretinoin versus isotretinoin alone in the treatment of high-risk neuroblastoma: impact on long-term survival. Value Health. 2019;22:S435 (abstract PCN4).

39. de Vries E, Cherny N, Latino N. The ESMO-Magnitude of Clinical Benefit Scale V1.1: user instructions and ESMO-MCBS case studies. Oncology/pro. <https://oncologypro.esmo.org/content/download/124996/2367014/1/Tips-Tricks-ESMO-MCBS-Detailed-Use-Guidelines-Case-Studies.pdf>. Accessed 16 Sept 2022.

40. Ellis LM, Bernstein DS, Voest EE, et al. American Society of Clinical Oncology perspective: raising the bar for clinical trials by defining clinically meaningful outcomes. J Clin Oncol. 2014;32(12):1277-80.

41. Laetsch TW, Myers GD, Baruchel A, Dietz AC, Pulsipher MA, Bittencourt H, et al. Patient-reported quality of life after tisagenlecleucel infusion in children and young adults with relapsed or refractory B-cell acute lymphoblastic leukaemia: a global, single-arm, phase 2 trial. Lancet Oncol. 2019;20(12):1710-8.

42. Maude SL, Laetsch TW, Buechner J, Rives S, Boyer M, Bittencourt H, et al. Tisagenlecleucel in Children and Young Adults with B-Cell Lymphoblastic Leukemia. N Engl J Med. 2018;378(5):439-48.

43. Mueller I, Ehlert K, Endres S, Pill L, Siebert N, Kietz S, et al. Tolerability, response and outcome of high-risk neuroblastoma patients treated with long-term infusion of anti-GD(2) antibody ch14.18/CHO. MAbs. 2018;10(1):55-61.

44. European Medicines Agency Committee for Medicinal Products for Human Use (CHMP). CHMP assessment report: Qarziba (EMEA/H/C/003918/0000). European Medicines Agency. 2017. <https://www.ema.europa.eu/en/medicines/human/EPAR/qarziba>. Accessed December 2020

45. Dourthe M-E, Rabian F, Yakouben K, Cabannes A, Chevillon F, Chaillou D, et al. Safety and Efficacy of Tisagenlecleucel (CTL019) in B-Cell Acute Lymphoblastic Leukemia in Children, Adolescents and Young Adults: The French Experience. Blood. 2019;134(Supplement_1):3876-.

46. Ghorashian S, Furness C, Cummins M, Snowden JA, O'Reilly MA, Roddie C, et al. Intention to Treat Analysis of Real-World Outcomes Following Tisgenlecleucel Therapy for Pediatric and Young Adult ALL through a National Access Programme. Blood. 2020;136:18-9.

47. European Medicines Agency Committee for Medicinal Products for Human Use (CHMP). CHMP assessment report: Kymriah (EMEA/H/C/004090/0000). European Medicines Agency. 2018. <https://www.ema.europa.eu/en/medicines/human/EPAR/kymriah>. Accessed December 2020

48. Baruchel A, Krueger J, Balduzzi A, Bittencourt H, De Moerloose B, Peters C, et al. TISAGENLECLEUCEL FOR PEDIATRIC/YOUNG ADULT PATIENTS WITH RELAPSED/REFRACTORY B-CELL ACUTE LYMPHOBLASTIC LEUKEMIA: PRELIMINARY REPORT OF B2001X FOCUSING ON PRIOR EXPOSURE TO BLINATUMOMAB AND INOTUZUMAB (S118). European Hematology Association 25 virtual2020.

49. Schultz LM, Baggott C, Prabhu S, Pacenta H, Phillips CL, Rossoff J, et al. Disease Burden Impacts Outcomes in Pediatric and Young Adult B-Cell Acute Lymphoblastic Leukemia after Commercial Tisagenlecleucel: Results from the Pediatric Real World CAR Consortium (PRWCC). Blood. 2020;136(Supplement 1):14-5.

50. Pasquini MC, Hu Z-H, Curran K, Laetsch T, Locke F, Rouce R, et al. Real-world evidence of tisagenlecleucel for pediatric acute lymphoblastic leukemia and non-Hodgkin lymphoma. Blood Adv. 2020;4(21):5414-24.

51. Ma Q, Zhang J, O'Brien E, Martin AL, Agostinho AC. Tisagenlecleucel versus historical standard therapies for pediatric relapsed/refractory acute lymphoblastic leukemia. Journal of Comparative Effectiveness Research. 2020;9(12):849-60.

52. Devinsky O, Cross JH, Laux L, Marsh E, Miller I, Nabbout R, et al. Trial of cannabidiol for drug-resistant seizures in the Dravet syndrome. N Engl J Med. 2017;376(21):2011-20.

53. Miller I, Scheffer IE, Gunning B, Sanchez-Carpintero R, Gil-Nagel A, Perry MS, et al. Dose-ranging effect of adjunctive oral cannabidiol vs placebo on convulsive seizure frequency in Dravet syndrome: a randomized clinical trial. JAMA Neurol. 2020;77(5):613-21.

54. Halford JJ, Scheffer I, Nabbout R, Sanchez-Carpintero R, Malawky YS, Wong M, et al. Long-term safety and efficacy of cannabidiol (CBD) treatment in patients with Dravet syndrome (DS): 3-year interim results of an open-label extension (OLE) trial (GWPCARE5). Neurology. 2020;94:Abstract 439.

55. Hassan E, Desai I, Soponski R, Muthugovindan D, Tan HJ. UK North West profile of Epidyolex use for refractory seizures in children. Developmental Medicine and Child Neurology 2021;63:92.

56. Koo CM, Kim SH, Lee JS, Park BJ, Lee HK, Kim HD, et al. Cannabidiol for Treating Lennox-Gastaut Syndrome and Dravet Syndrome in Korea. J Korean Med Sci. 2020;35(50):e427-e.

57. Nabbout R, Sullivan J, Dlugos D, Farfel G, Galer BS, Morrison G, et al. What Defines “Clinically Meaningful Changes in Seizure Frequency”? Analysis of Data From a Phase 3 Clinical Study of Low-Dose Fintepla® (Fenfluramine HCl Oral Solution) in Dravet Syndrome. American Epilepsy Society (AES) Annual Meeting; New Orleans, LA2018.

58. Chiron C, Marchand MC, Tran A, Rey E, d'Athis P, Vincent J, et al. Stiripentol in severe myoclonic epilepsy in infancy: a randomised placebo-controlled syndrome-dedicated trial. STICLO study group. Lancet. 2000;356(9242):1638-42.

59. Devinsky O, Patel AD, Cross JH, Villanueva V, Wirrell EC, Privitera M, et al. Effect of cannabidiol on drop seizures in the Lennox-Gastaut syndrome. N Engl J Med. 2018;378(20):1888-97.

60. Thiele EA, Marsh ED, French JA, Mazurkiewicz-Beldzinska M, Benbadis SR, Joshi C, et al. Cannabidiol in patients with seizures associated with Lennox-Gastaut syndrome (GWPCARE4): a randomised, double-blind, placebo-controlled phase 3 trial. Lancet. 2018;391(10125):1085-96.

61. Patel A, Chin R, Mitchell W, Perry S, Weinstock A, Checketts D, et al. Long-term safety and efficacy of cannabidiol (CBD) treatment in patients with Lennox Gastaut syndrome (LGS): 3-year results of an open-label extension (OLE) trial (GWPCARE5). Neurology. 2020;94:Abstract 668.

62. European Medicines Agency Committee for Medicinal Products for Human Use (CHMP). CHMP assessment report: Myalepta (EMEA/H/C/004218/0000). European Medicines Agency. 2018. <https://www.ema.europa.eu/en/medicines/human/EPAR/myalepta>. Accessed December 2020

63. Cook K, Stears A, Araujo-Vilar D, Santini F, Stephen O, Ceccarini G, et al. Real-world experience of generalized and partial lipodystrophy patients enrolled in the metreleptin early access program. Endocrine Abstracts. 2019;63:586.

64. Brown RJ, Oral EA, Cochran E, Araújo-Vilar D, Savage DB, Long A, et al. Long-term effectiveness and safety of metreleptin in the treatment of patients with generalized lipodystrophy. Endocrine. 2018;60(3):479-89.

65. Imel EA, Glorieux FH, Whyte MP, Munns CF, Ward LM, Nilsson O, et al. Burosumab versus conventional therapy in children with X-linked hypophosphataemia: a randomised, active-controlled, open-label, phase 3 trial. Lancet. 2019;393(10189):2416-27.

66. Padidela R, Whyte MP, Glorieux FH, Munns CF, Ward LM, Nilsson O, et al. Patient-Reported Outcomes from a Randomized, Active-Controlled, Open-Label, Phase 3 Trial of Burosumab Versus Conventional Therapy in Children with X-Linked Hypophosphatemia. Calcif Tissue Int. 2021;108(5):622-33.

67. Imel EA, Glorieux FH, Whyte MP, Portale AA, Munns CF, Nilsson O, et al. In Pediatric X-linked Hypophosphatemia (XLH), Burosumab Improved Clinical Outcomes Versus Higher and Lower Doses of Oral Phosphate and/or Active Vitamin D. Journal of Bone and Mineral Research. 2020;35:150.

68. Whyte MP, Carpenter TO, Gottesman GS, Mao M, Skrinar A, San Martin J, et al. Efficacy and safety of burosumab in children aged 1-4 years with X-linked hypophosphataemia: a multicentre, open-label, phase 2 trial. Lancet Diabetes Endocrinol. 2019;7(3):189-99.

69. Thacher TD, Pettifor JM, Tebben PJ, Creo AL, Skrinar A, Mao M, et al. Rickets severity predicts clinical outcomes in children with X-linked hypophosphatemia: Utility of the radiographic Rickets Severity Score. Bone. 2019;122:76-81.

70. European Medicines Agency Committee for Medicinal Products for Human Use (CHMP). CHMP assessment report: Crysvita (EMEA/H/C/004275/0000). European Medicines Agency. 2020. <https://www.ema.europa.eu/en/medicines/human/EPAR/crysvita>. Accessed December 2020

71. Carpenter TO, Högler W, Imel EA, Portale AA, Boot AM, Linglart A, et al. Continued improvement in clinical outcomes with long-term burosumab, a fully human anti-FGF23 monoclonal antibody: results from a 3-year, phase 2, clinical trial in children with X-linked hypophosphatemia (XLH). J Bone Miner Res. 2019;34:Abstract 1037.

72. Brener A, Lebenthal Y, Cleper R, Kapusta L, Zeitlin L. Body composition and cardiometabolic health of pediatric patients with X-linked hypophosphatemia (XLH) under burosumab therapy. Ther Adv Endocrinol Metab. 2021;12:20420188211001150-.

73. Imel E, Carpenter T, Gottesman G, Chen A, Skrinar A, Roberts MS, et al. Three-Year Safety and Efficacy Results of Burosumab for Children Aged 1 to 4 years with X-linked Hypophosphatemia (XLH). Journal of Bone and Mineral Research. 2020;35:16.

74. Chen CX, Kroenke K, Stump TE, Kean J, Carpenter JS, Krebs EE, et al. Estimating minimally important differences for the PROMIS pain interference scales: results from 3 randomized clinical trials. Pain. 2018;159(4):775-82.

75. Harmatz P, Whitley CB, Wang RY, Bauer M, Song W, Haller C, et al. A novel Blind Start study design to investigate vestronidase alfa for mucopolysaccharidosis VII, an ultra-rare genetic disease. Mol Genet Metab. 2018;123(4):488-94.

76. European Medicines Agency Committee for Medicinal Products for Human Use (CHMP). CHMP assessment report: Mepsevii (EMEA/H/C/004438/0000). European Medicines Agency. 2018. <https://www.ema.europa.eu/en/medicines/human/EPAR/mepsevii>. Accessed December 2020

77. Lau HA, Viskochil D, Tanpaiboon P, Gonzalez-Meneses Lopez A, Martins E, Taylor J, et al. Long-term efficacy and safety of vestronidase alfa enzyme replacement therapy in pediatric subjects with mucopolysaccharidosis VII < 5 years old. Molecular Genetics and Metabolism. 2020;129:S95.

78. Wang RY, da Silva Franco JF, López-Valdez J, Martins E, Sutton VR, Whitley CB, et al. The long-term safety and efficacy of vestronidase alfa, rhGUS enzyme replacement therapy, in subjects with mucopolysaccharidosis VII. Mol Genet Metab. 2020;129(3):219-27.

79. Gonzalez-Meneses Lopez AGL, Beuno MB, Lau HL, Viskochil DV, Tanpaiboon PT, Martins EM, et al. P-371 Vestronidase alfa stabilizes or improves disease manifestations in subjects with MPS VII. J Inherit Metab Dis. 2018;41:S187-S8.

80. Borgwardt L, Guffon N, Amraoui Y, Dali CI, De Meirleir L, Gil-Campos M, et al. Efficacy and safety of Velmanase alfa in the treatment of patients with alpha-mannosidosis: results from the core and extension phase analysis of a phase III multicentre, double-blind, randomised, placebo-controlled trial. J Inherit Metab Dis. 2018;41(6):1215-23.

81. European Medicines Agency Committee for Medicinal Products for Human Use (CHMP). CHMP assessment report: Lamzede (EMEA/H/C/003922/0000). European Medicines Agency. 2018. <https://www.ema.europa.eu/en/medicines/human/EPAR/lamzede>. Accessed December 2020

82. Dempster H, Porepa M, Young N, Feldman BM. The clinical meaning of functional outcome scores in children with juvenile arthritis. Arthritis Rheum. 2001;44(8):1768-74.

83. Borgwardt L, Guffon N, Amraoui Y, Jones SA, De Meirleir L, Lund AM, et al. Health related quality of life, disability, and pain in alpha mannosidosis: Long-term data of enzyme replacement therapy with velmanase alfa (human recombinant alpha mannosidase). J Inborn Errors Metab Screen. 2018;6.

84. Dhanani S, Quenneville J, Perron M, Abdolell M, Feldman BM. Minimal difference in pain associated with change in quality of life in children with rheumatic disease. Arthritis Rheum. 2002;47(5):501-5.

85. Kohn CG, Sidovar MF, Kaur K, Zhu Y, Coleman CI. Estimating a minimal clinically important difference for the EuroQol 5-Dimension health status index in persons with multiple sclerosis. Health Qual Life Outcomes. 2014;12:66.

86. Pearson ER, Flechtner I, Njølstad PR, Malecki MT, Flanagan SE, Larkin B, et al. Switching from insulin to oral sulfonylureas in patients with diabetes due to Kir6.2 mutations. N Engl J Med. 2006;355(5):467-77.

87. Beltrand J, Elie C, Busiah K, Fournier E, Boddaert N, Bahi-Buisson N, et al. Sulfonylurea Therapy Benefits Neurological and Psychomotor Functions in Patients With Neonatal Diabetes Owing to Potassium Channel Mutations. Diabetes Care. 2015;38(11):2033-41.

88. Bowman P, Sulen Å, Barbetti F, Beltrand J, Svalastoga P, Codner E, et al. Effectiveness and safety of long-term treatment with sulfonylureas in patients with neonatal diabetes due to KCNJ11 mutations: an international cohort study. Lancet Diabetes Endocrinol. 2018;6(8):637-46.

89. European Medicines Agency Committee for Medicinal Products for Human Use (CHMP). CHMP assessment report: Amglidia (EMEA/H/C/004379/0000). European Medicines Agency. 2018. <https://www.ema.europa.eu/en/medicines/human/EPAR/amglidia>. Accessed December 2020

90. Duell PB, Salen G, Eichler FS, DeBarber AE, Connor SL, Casaday L, et al. Diagnosis, treatment, and clinical outcomes in 43 cases with cerebrotendinous xanthomatosis. J Clin Lipidol. 2018;12(5):1169-78.

91. Salen G, Berginer V, Shore V, Horak I, Horak E, Tint GS, et al. Increased concentrations of cholestanol and apolipoprotein B in the cerebrospinal fluid of patients with cerebrotendinous xanthomatosis. Effect of chenodeoxycholic acid. N Engl J Med. 1987;316(20):1233-8.

92. Amador MDM, Masingue M, Debs R, Lamari F, Perlbarg V, Roze E, et al. Treatment with chenodeoxycholic acid in cerebrotendinous xanthomatosis: clinical, neurophysiological, and quantitative brain structural outcomes. J Inherit Metab Dis. 2018;41(5):799-807.

93. Verrips A, Dotti MT, Mignarri A, Stelten BML, Verma S, Federico A. The safety and effectiveness of chenodeoxycholic acid treatment in patients with cerebrotendinous xanthomatosis: two retrospective cohort studies. Neurol Sci. 2020;41(4):943-9.

94. DeBarber AE, Luo J, Giugliani R, Souza CFM, Chiang JP-W, Merkens LS, et al. A useful multi-analyte blood test for cerebrotendinous xanthomatosis. Clin Biochem. 2014;47(9):860-3.

95. Costelloe L, O'Rourke K, Kearney H, McGuigan C, Gribbin L, Duggan M, et al. The patient knows best: significant change in the physical component of the Multiple Sclerosis Impact Scale (MSIS-29 physical). J Neurol Neurosurg Psychiatry. 2007;78(8):841-4.

96. Canadian Agency for Drugs and Technologies in Health. Appendix 4, Validity of Outcome Measures Clinical Review Report: Ocrelizumab (Ocrevus): (Hoffmann-La Roche Limited): Indication: Management of adult patients with early primary progressive multiple sclerosis as defined by disease duration and level of disability, in conjunction with imaging features characteristic of inflammatory activity [Internet]. Ottawa, ON: Canadian Agency for Drugs and Technologies in Health; 2018.

97. Stelten BML, Huidekoper HH, van de Warrenburg BPC, Brilstra EH, Hollak CEM, Haak HR, et al. Long-term treatment effect in cerebrotendinous xanthomatosis depends on age at treatment start. Neurology. 2019;92(2):e83-e95.

98. Broderick JP, Adeoye O, Elm J. Evolution of the Modified Rankin Scale and Its Use in Future Stroke Trials. Stroke. 2017;48(7):2007-12.

99. Maas RPPWM, van de Warrenburg BPC. Exploring the clinical meaningfulness of the Scale for the Assessment and Rating of Ataxia: A comparison of patient and physician perspectives at the item level. Parkinsonism & Related Disorders. 2021;91:37-41.

100. Schulz A, Specchio N, Gissen P, de los Reyes E, Slasor P, Jacoby D. Persistent treatment effect of cerliponase alfa in children with CLN2 disease: A > 4 year update from an ongoing multicenter extension study. Molecular Genetics and Metabolism. 2020;129(2):S145.

101. Canadian Agency for Drugs and Technologies in Health. Clinical Review Report: Cerliponase Alfa (Brineura): (Biomarin Pharmaceutical (Canada) Inc.): Indication: For the treatment of neuronal ceroid lipofuscinosis type 2 (CLN2) disease, also known as tripeptidyl peptidase 1 (TPP1) deficiency [Internet]. Canadian Agency for Drugs and Technologies in Health. 2019. <https://www.ncbi.nlm.nih.gov/books/NBK544809/>. Accessed

102. Wibbeler E, Wang R, Reyes EdL, Specchio N, Gissen P, Guelbert N, et al. Cerliponase Alfa for the Treatment of Atypical Phenotypes of CLN2 Disease: A Retrospective Case Series. J Child Neurol. 2021;36(6):468-74.

103. Wyrwich KW, Schulz A, Nickel M, Slasor P, Ajayi T, Jacoby DR, et al. An Adapted Clinical Measurement Tool for the Key Symptoms of CLN2 Disease. Journal of Inborn Errors of Metabolism and Screening. 2018;6:2326409818788382.
